# Harnessing robotic automation and web-based technologies to modernize scientific outreach

**DOI:** 10.1371/journal.pbio.3000348

**Published:** 2019-06-26

**Authors:** Orna Dahan, Bat-Shahar Dorfman, Serkan Sayin, Brittany Rosener, Tiffany Hua, Anat Yarden, Amir Mitchell

**Affiliations:** 1 Department of Molecular Genetics, Weizmann Institute of Science, Rehovot, Israel; 2 Department of Science Teaching, Weizmann Institute of Science, Rehovot, Israel; 3 Program in Systems Biology, University of Massachusetts Medical School, Worcester, Massachusetts, United States of America; 4 Program in Molecular Medicine, University of Massachusetts Medical School, Worcester, Massachusetts, United States of America

## Abstract

Technological breakthroughs in the past two decades have ushered in a new era of biomedical research, turning it into an information-rich and technology-driven science. This scientific revolution, though evident to the research community, remains opaque to nonacademic audiences. Such knowledge gaps are likely to persist without revised strategies for science education and public outreach. To address this challenge, we developed a unique outreach program to actively engage over 100 high-school students in the investigation of multidrug-resistant bacteria. Our program uses robotic automation and interactive web-based tools to bridge geographical distances, scale up the number of participants, and reduce overall cost. Students and teachers demonstrated high engagement and interest throughout the project and valued its unique approach. This educational model can be leveraged to advance the massive open online courses movement that is already transforming science education.

## Abstract 2

This Community Page article describes the use of robotic automation and web-based tools to allow high-school students around the world to run empirical experiments remotely and to collaboratively investigate the emergence of multidrug resistance during bacterial evolution.

## Challenges in science education

Technological breakthroughs, such as genomic sequencing and robotic automation, are radically transforming life-sciences research and clinical medicine. Future scientific discoveries and technological breakthroughs will greatly benefit from an educational system that emphasizes inquiry-based scientific thinking and exposes students to recent advances in relevant fields. Inquiry and engagement in scientific practices—in which students are expected to learn from firsthand experience [1]—are central elements in many science curricula worldwide [1–3]. Indeed, many studies have shown that inquiry-based learning has positive effects on students’ engagement, conceptual understanding, critical thinking skills, and more (reviewed in [4,5]).

Methodologies commonly used in schools, however, are considerably different from authentic research. Although in recent years several inquiry tasks were developed to capture features of authentic research, many classroom inquiry tasks still focus on simple inquiry, with a step-by-step approach to reach predetermined outcomes [6]. Even in hands-on experimentation in schools, these “cookbook” procedures rarely produce unexpected results requiring real-time decision-making or the opportunity to investigate further. Moreover, in some cases when students do encounter unexpected results, they learn to interpret them as evidence of a failure in executing the experiment. As a result, students often acquire misleading ideas about how scientific inquiry works that may hinder scientific literacy and affect students' attitudes toward science [7].

Yet science educators who want to implement authentic scientific inquiry face many challenges, including limited resources and safety concerns, a lack of real-world experience with scientific inquiry in the classroom [6,7], inaccessibility to modern scientific facilities [6], and difficulties in scaling up and replicating successful outreach initiatives [8]. These barriers dramatically narrow the student’s access to modern research practices that are routinely used by scientists today. Bridging these persistent gaps will not only transform how science is taught but also change how it is perceived by the public. Here, we present a new approach to science outreach that can overcome many of these limitations to help achieve these goals.

The ability of organisms to evolve and adapt to new environments is a key biological process taught in numerous science curricula around the world [9]. One related phenomenon that has attracted much attention from the public and biomedical community alike is the emergence of antibiotic-resistant bacteria. A powerful approach to study this phenomenon in lab settings is experimental evolution [10]. In such experiments, bacterial cultures are propagated from an ancestral strain for many generations in controlled environments that continually select for drug resistance. Excitingly, such experiments uncover rigorous connections between environmental pressure, genetic changes, and phenotypic outcomes. Moreover, the results of these experiments can be readily communicated with nonacademic audiences [11,12]. We therefore decided to utilize this methodology to develop an inquiry-based outreach project that will teach high-school students about the emergence of antibiotic resistance by allowing them to explore how alternative drug regimens channel evolutionary adaptation toward multidrug resistance.

## Project overview

We set out to investigate whether a new approach can be implemented in order to modernize science education for high-school students while tackling the key challenges we perceive in current approaches (S1 Text). Toward this aim, we developed an online robotic platform that allows over 100 participants to carry out multiday empirical experiments remotely in our lab through the use of a standard internet connection and freely available online tools. We teamed up with seven high schools from Israel and the United States to carry out a collaborative lab evolution experiment with 150 students in the spring of 2018. The project was repeated during the spring of 2019 with 88 students from six high-school classes across the US (the reported results are for the 2018 iteration). The overall aim was to examine how antibiotic resistance emerges within short evolutionary timescales in the model microorganism *Escherichia coli* and to investigate its molecular underpinnings. The project consisted of three individual modules (preparation, experimentation, and bioinformatics analysis) and relied on real-time mentoring by class teachers and two scientists. Lastly, we used anonymous student and teacher questionnaires to monitor students’ cognitive and behavioral engagement and overall views on the methodology in order to evaluate the potential contribution of our educational approach.

## Project preparation

The 150 project participants came from different age groups (15–17 years of age), educational systems (7 schools in Israel and the US), and socioeconomic backgrounds. We therefore developed teaching aids that allowed teachers to front-load students with adequate academic background before participation (S1 Presentation). We also formed a closed discussion forum to allow scientists and teachers to converse and prepare before engaging students with the project. At the beginning of the project, students conducted simple preliminary experiments at their schools’ laboratories in order to familiarize them with basic concepts in bacterial growth and antibiotic resistance. These included monitoring how bacterial growth (*E*. *coli* strain MG-1655) is affected by antibiotics (types and concentration) as measured by changes in optical density of liquid cultures (S2 Text). Toward this aim, we developed, fabricated, and distributed an open-source spectrophotometer (Box 1).

**Box 1. Technology box**

**Open-source spectrophotometer**

Teachers and students in Israel conducted preliminary experiments to explore bacterial growth phases and the inhibitory effects of antibiotics. We provided classes with an open-source spectrophotometer we designed ourselves (S3 Fig). We used an Arduino microcontroller and standard electrical components (light-emitting diode, light sensor, digit display) to build an electric circuit that can measure optical density of bacterial cultures in standard 2-ml tubes (S7 Text, S2 Code). The circuit was housed in a small laser-cut acrylic box and a 3D-printed plastic holder we designed (S1 Design, S2 Design). Importantly, the low cost of all components allowed us to fabricate a spectrophotometer for less than $15 and to distribute spectrophotometers freely to participating classes with a simple instructions manual (S8 Text).

**Online robotic platform**

The online robotic platform was based on a commercial liquid handler (epMotion/Eppendorf) and a set of computer programs we developed to automate its operation with minimal human intervention (S4 Text). On a daily basis, students updated the drug regimen of bacterial cultures (antibiotic type and concentration) through Google sheets that were separately shared with each class (S5 Text). We developed a computer program (Matlab/Mathworks) that operated daily to download the drug regimen choices and to convert these into instructions readable by the liquid handler (S1 Code). We used YouTube to broadcast the liquid handler operation as a livestream using two onboard web cameras. We spiked drug stocks with inert food dyes to allow online viewers to observe and validate that the robotic liquid handler indeed implemented the drug regimen they chose (color and intensity reflected antibiotic type and concentration). Once a new 96-well plate was prepared, the liquid handler diluted bacterial cultures from the previous day at a 1:100 dilution. We used a microplate absorbance reader (TECAN) to monitor changes in optical density over 6 hours of growth. We used a computer script to infer the generation time of all cultures (Matlab/Mathworks) and generate a graphical representation of the results (S1 Code). An image with inferred doubling time was posted online at the project’s website to allow students to make informed decisions for the next day of the experiment. All Google sheets and results were integrated into the project’s website to allow live sharing of data and results.

## Online evolution experiment

**Fig 1:**
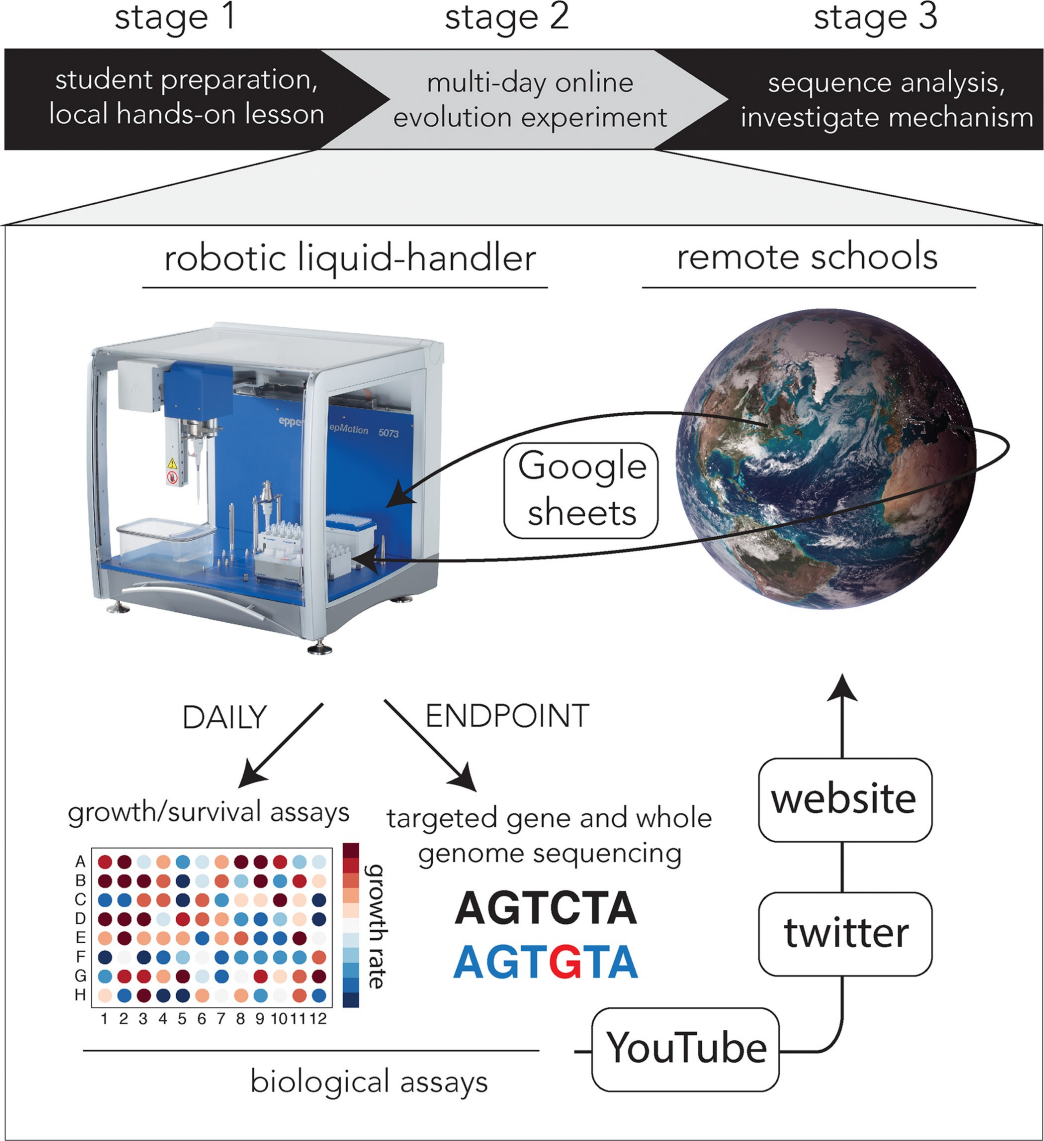
Overview of information flow during the online evolution experiment. The interactive evolution experiment was the second stage in the outreach project and took place over a period of 10 days. On a daily basis, students used online Google sheets to inform a robotic liquid handler on a drug regimen they wanted to implement (drug type and concentration). Liquid handling operation (plate preparation and culture dilution) was streamed as a live broadcast on YouTube by two onboard cameras. We shared systematic reports on culture growth rate by posting them on the project’s website and also shared photos showing bacterial confluences in the discussion forum and on Twitter. The daily results were used by students to make informed decisions on changes in the drug regimen for the following day. At the end of the experiment, sequences and reports from targeted and whole-genome sequencing were shared with students to provide clues on the potential molecular mechanisms underlying evolved drug resistance. *Earth photograph (GSFC_20171208_Archive_e002131)*: *NASA image and video library*.

**Fig 2:**
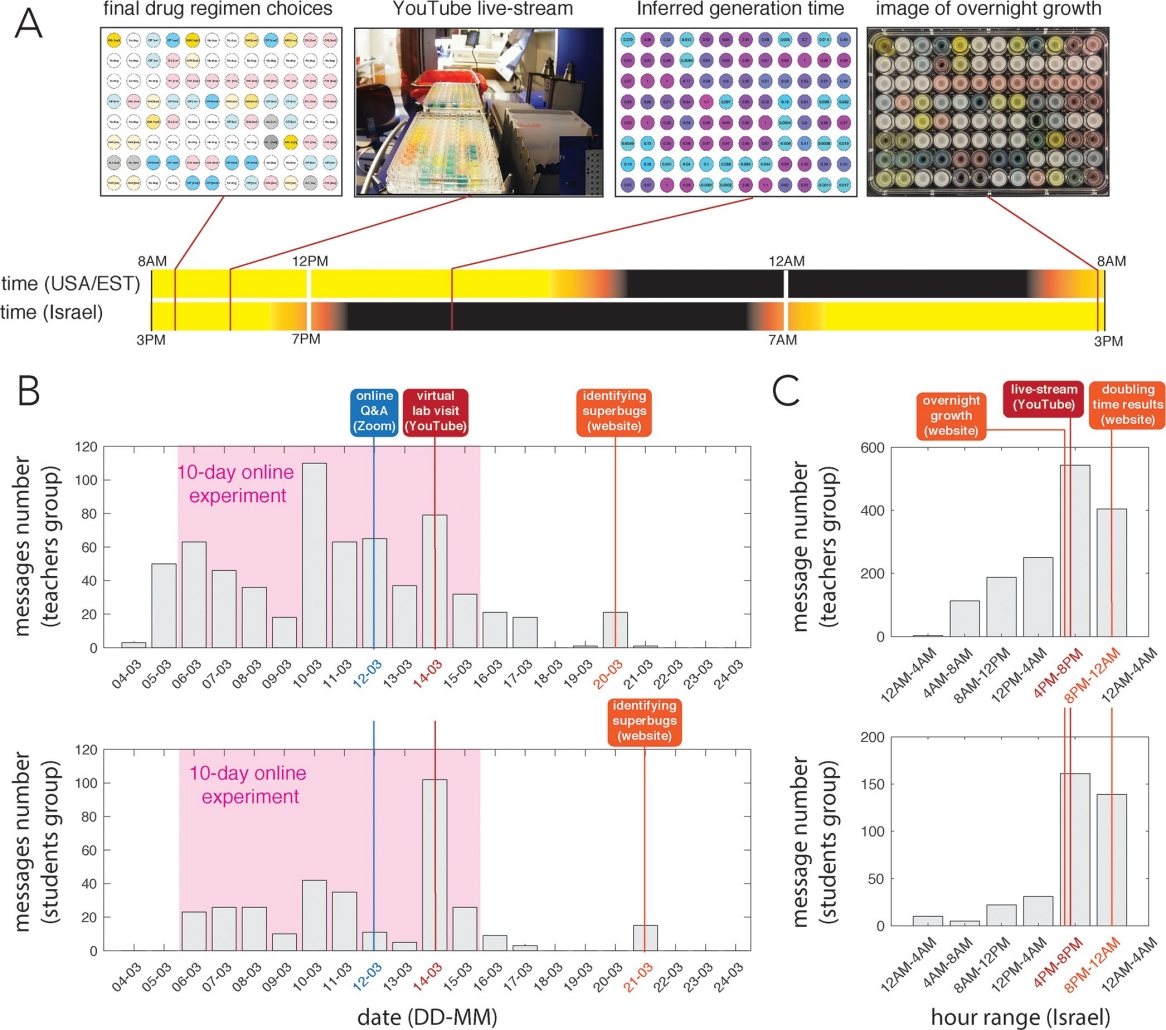
Daily interactions and information exchange during the evolution experiment. (A) The daily schedule and information sharing scheme between the research lab (USA) and the remote high schools (Israel). Drug regimen choices were made daily, and plate preparation and culture inoculation were streamed live over YouTube (the experiment continued over 10 consecutive days). Different antibiotics were spiked by inert color dyes to allow students to visually confirm the robotic liquid handler implemented the correct drug regimen. The research lab reported back the observed generation time and also posted a photograph of culture confluence to allow students to make informed decisions on the next day’s drug regimen. (B) Analysis of the social interactions in the discussion forum during the experiment showing overall increase in the number of messages during the 10-day experiment. Online events, such as a virtual lab visit, had notable effects on the number of interactions. The “identifying superbugs” results were shared with teachers a day before the students. (C) Analysis of social interactions timing. The analysis reveals many of the interactions occurred after school hours and correlated with important daily events. Q&A, question-and-answer session.

The main part of the project comprised a 10-day lab evolution experiment that was fully managed by the participating students through a host of online interactive technologies (Fig 1). The overall aim proposed to the students was to investigate which selection regimens culminate in multidrug resistance (S3 Text). In this phase, multiple *E*. *coli* cultures (MG-1655) were diluted and passaged daily in liquid media supplemented with antibiotics (S4 Text). Each group of students (typically 2–4) was asked to design an experimental protocol that will test alternative selection strategies and will investigate different routes toward antibiotic resistance (using 2–3 biological repeats). Each participating class was allocated with 12 individual wells within the 96-well microplate and was asked to determine the desired drug regimen on a daily basis (type and concentration from 13 predefined alternatives). These daily decisions were communicated to the robotic liquid handler directly through online shared Google sheets (S4 and S5 Text). Students could then view both the operation of the robotic platform and reports of growth dynamics over the internet (available on the project’s website https://mitchell-lab.umassmed.edu/evolution). These daily reports allowed the students to make informed decisions on changes in drug regimens on a daily basis as evolutionary adaptation took place and to update their instructions accordingly (Fig 1). For example, if students observed that bacteria are no longer inhibited by a drug, they could choose to increase the drug concentration or to treat the culture with an alternative antibiotic on the consecutive day. Moreover, since all results were shared in real time, students also used information from other groups to inform their daily decisions. Fig 2A shows the daily routine during the evolution experiment and the data sharing schedule.

To increase students’ engagement and invoke lively scientific discussions, we set up joint discussion groups and also held several virtual video meetings (Skype/Zoom). We found that informal text discussions were the most engaging tool for scientific communication. A discussion forum with Israeli teachers and scientists had 1,361 messages, and a separate anonymous discussion forum with Israeli students had 328 messages. Fig 2B depicts the texting trends during the experiment days and reveals an overall increase in texting frequency in both texting groups. The effects of additional interactions during this period is also observed (e.g., decrease after an online questions-and-answers session and a considerable increase associated with an online lab visit). Fig 2C depicts the texting trends at different hours of the days. These results reveal that students were highly engaged with the project after school hours during the live broadcast and when results were posted online. Between 12 and 45 viewers watched the daily YouTube broadcasts. Analysis of the daily decisions the students made throughout the 2019 experiment suggests that logical reasoning improved as the experiment progressed (S1 Fig).

In the end of the evolution experiment, we provided students with systematic measurements of evolved drug resistance across a panel of antibiotics (https://mitchell-lab.umassmed.edu/selection-outcome). These results allowed students to evaluate whether the drug regimen they devised ultimately selected for multidrug resistance. Remarkably, these measurements and critical discussions triggered a discussion on related but more complex phenomena, such as evolved cross-sensitivity and its potential applications in the clinic. Students’ choices, experiment results, and YouTube clips can be found on the project’s website (https://mitchell-lab.umassmed.edu/evolution). Details on guidelines for the teachers (S3 Text), experimental protocol (S4 Text), Google sheets and YouTube setup (S5 Text), equipment list (S6 Text), and computer code (S1 Code) are provided as Supporting Information.

## Analysis of genomic sequencing

In order to provide the students with some insight into the molecular mechanisms driving resistance, we developed a bioinformatics component to conclude the project. We also provided teachers with an introductory presentation on sequence analysis (S2 Presentation). At this stage, all schools analyzed the same dataset. First, we used targeted Sanger sequencing to detect mutations in two genes previously associated with resistance to the used antibiotics [13]. We performed 40 targeted sequencing reactions on cultures from 20 different wells and identified loss-of-function mutations in two of the cultures. We shared the two mutated sequences, along with the ancestral sequences, with the students through the project’s website. Classes used these sequences for an introduction lesson on bioinformatics and preformed pairwise sequence alignments using BLAST at the NCBI website [14] to identify the mutations. The observation that mutations were observed in only a small fraction of sequenced genes was used to prompt a discussion about the potential mechanisms underlying drug resistance in the vast majority the other resistant strains. Furthermore, it was used to rationalize the necessity of sequencing the entire genome. Finally, we sequenced the entire genome of the ancestor strain and a single evolved strain that was identified as a multidrug resistor. As analysis of this dataset was too demanding for students, we performed the analysis ourselves using breseq [15]. We identified and reported back to teachers about inactivating mutations in three different genes. Teachers used this information to lead a discussion with their students on the likely driver mutation and on the possibility of neutral hitchhiking mutations.

## Project impact

**Fig 3:**
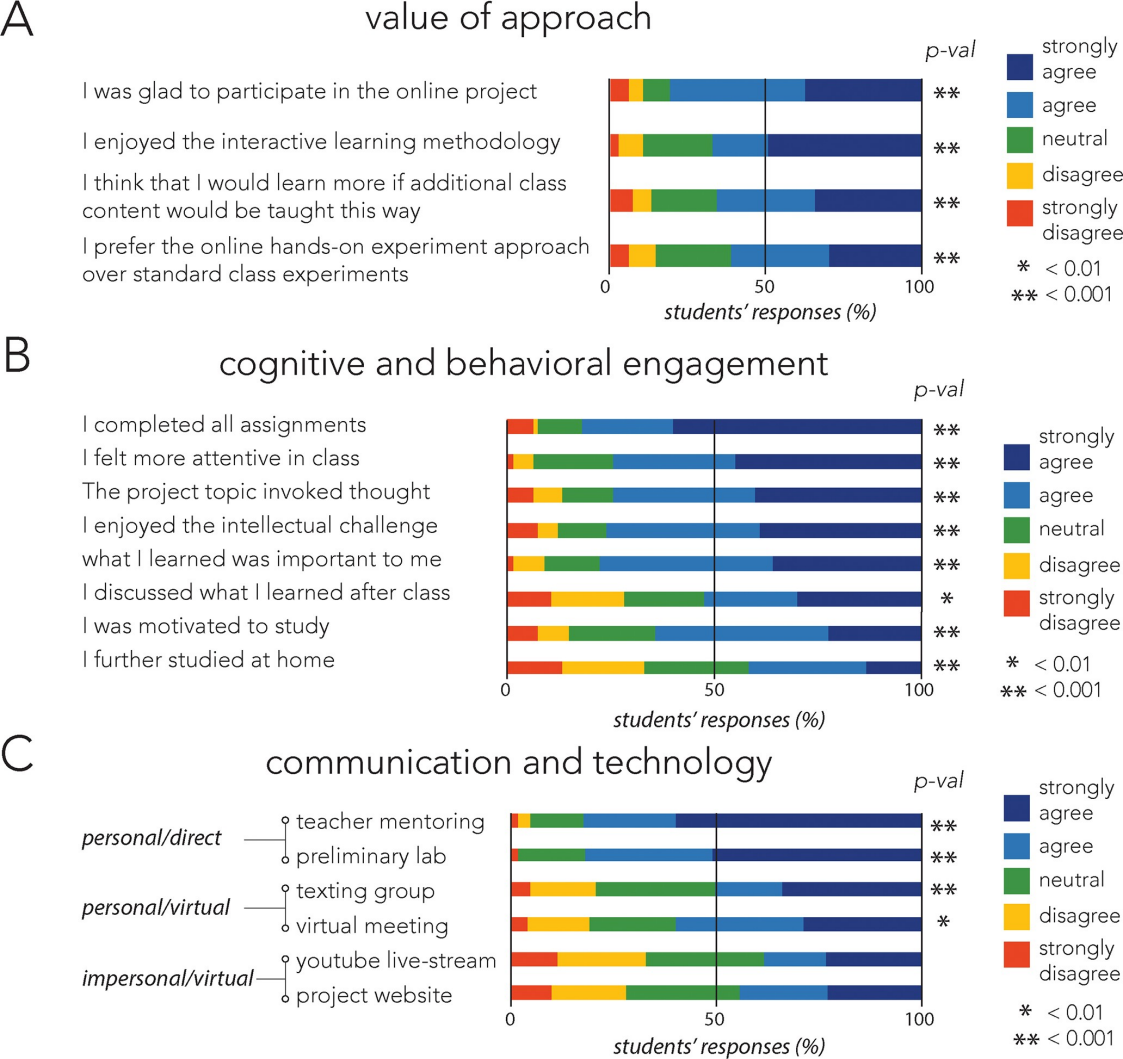
Results from student questionnaires and statistical significance for deviation from neutral (Wilcoxon test). (A) Students’ self-reporting on the value of interactive approach (*N* = 67). (B) Students’ self-reporting on cognitive and behavioral engagement (*N* = 67). (C) Students’ self-reporting on the value of different teaching and communication technologies (*N* = 42–69). The questionnaires were filled out by Israeli students, and results reflect the relative fraction of each response category collected from all responding students. Results from teacher questionnaires are provided as S2 Fig.

In order to evaluate the effectiveness of the project, Israeli students and teachers were asked to voluntarily complete anonymous feedback questionnaires upon the completion of the project. The questionnaires included self-reported items that assessed students' cognitive and behavioral engagement as well as overall views on the approach. Most items were adapted from previously reported questionnaires [16–18], and some were newly developed for our assessment. Overall, students highly valued the approach (Fig 3A). Over 80% of responding students were happy they participated in this project, and over 65% enjoyed the interactive features and felt they would have learned more if more biology lessons would have been taught this way. When asked to explain why, most of them wrote it was a unique, interesting, and challenging experience for them. Some added that they enjoyed being a part of a "real" scientific experiment and see things such as mutations and evolution they would likely never observe in the school settings.

Most students reported high cognitive engagement and that they were motivated to study and discuss the project beyond class hours (Fig 3B). Over 74% reported that what they learned was important to them, that it inspired them to think beyond class, and that they enjoyed the intellectual challenge. Behavioral engagement was also high, as over 65% of responding students reported that they were attentive in class and completed the assignments. One of our aims was to evaluate which of the technological tools we offered were the most impactful for students (Fig 3C). The questionnaires revealed that students most valued the direct interactions with teachers and the preliminary experiments, followed by the online interactions through video call and text messaging. Although passive online technologies that did not involve interaction with a teacher or scientist, such as the project website and daily broadcasts, were still valued by students, they ranked lowest in students’ questionnaires.

Teachers also completed feedback questionnaires, and their impressions highly overlapped with those of their students (S2A Fig and S2B Fig). In addition, leveraging teachers’ past experiences in teaching these topics, we asked them to evaluate the project’s contribution to students’ understanding and academic achievements relative to the standard curriculum and teaching approach. The teachers’ evaluations revealed that the project considerably contributed to the classes’ academic achievements and understanding (S2C Fig). However, teachers varied in their estimations of the fraction of the students that benefited from it. This variation may be explained by a difference in the fraction of students that actively engaged with the daily decisions in the different classes or due to other unaccounted differences between the classes (S2C Fig). All teachers were very glad they participated, despite the divergence it required from the standard curriculum, and were eager to participate in future iterations of the project. They found that the online communication tools were very helpful. Particularly, they valued the communication channels that allowed them to consult directly with the scientists and with each other in closed forums. The unanimous appreciation by teachers is important, since local teachers’ participation is critical to our educational approach, as they typically are not trained in this kind of experimentation.

## Project pitfalls and potential improvements

Over the past 2 years, we ran the outreach project twice and have evaluated not only its educational potential but also its inherent pitfalls from the classroom perspective. The central challenges we encountered concern the project’s timeline and integration with existing high-school curriculum. For example, we addressed the challenge of integrating the multistage project into an existing, typically crowded, high-school curriculum by restricting this project participation to senior high-school classes with sufficient academic background (however, this may not be possible in countries, such as Israel, that have crowded curricula and final exams in the senior year). A related challenge, keeping students engaged with the project after the intensive stage of the evolution experiment concludes, is almost unavoidable, since some of the follow-up experiments are time consuming. In the 2019 project, we found that this challenge can be partly mitigated by efficient planning and very quick execution of the follow-up experiments by the research lab (systematic measurements of drug sensitivity and targeted gene sequencing). Whereas minimization of the “waiting” time is less critical in the academic setting, it emerged as crucial for keeping students excited about the discoveries they can make on the molecular mechanisms driving evolved drug resistance.

Lastly, it is important to mention that a few schools had difficulties executing the preparation lab lesson because they lacked access to basic lab equipment, such as pipettes and tube holders. We predict that similar challenges will be common to many countries. We believe this gap can be addressed by providing such schools with a kit that includes all required consumables and additional open-source fabricated equipment. Moreover, the lab protocol accompanying this stage can be adjusted to use baking yeast and store-bought chemicals, instead of bacteria and antibiotics. Since all other project stages are executed online, this adjustment to the preparation stage will allow even remote and completely unequipped schools to participate in the outreach project.

## Conclusions

Science education and outreach have greatly benefited from advances in online interactive technologies and the low costs of internet connectivity. The emergence of massive online open courses (MOOCs), which bridge geographical distances and scale up the number of participants, is among the most profound changes in education. However, as MOOC programs became popular, their problems have also become more pronounced; these include the low student retention, their impersonal nature, and their inability to encompass hands-on experimentation [19–21]. Specifically, for life-science education, we and many others have previously developed websites that allow students to gain experiences in virtual labs and perform bioinformatics analysis [22–25]. Some have even developed online microscopy and cloud experimentation platforms that allow users to directly interact with microorganisms in real time [26,27]. However, despite their success, these virtual approaches are inadequate to capture key aspects of authentic scientific research, including their unpredictability and the requirement to make real-time decisions as experiments unfold. These gaps hamper the excitement that stems from discovering a truly new piece of biological insight. To address these gaps, we developed an educational approach that leverages robotic automation and virtual tools to enable authentic scientific research and to facilitate direct interactions between students and scientists on a massive scale.

Our own experience in this project and the personal interviews we conducted with students and teachers helped us better elucidate the unique contribution our approach can make to science education and outreach. Unlike many academic outreach initiatives, our project was developed to become an integral part of classroom education and to be led by classroom teachers that did not receive prior training. From the student’s perspective, the project is viewed as a technology-driven extension of academic subjects that are covered in the classroom (e.g., evolution, drug resistance, genomics). Yet, beyond exposure to new and exciting technologies, the students deeply valued the inquiry-based investigation strategy and were excited about the ownership they felt over their own experiments, results, and discoveries. This excitement was amplified by the open-access environment (real-time exposure to choices and results of all participants), which led to a ground-up competitive yet highly collaborative atmosphere. From the teacher’s perspective, the project offers a powerful strategy to actively engage students in cutting-edge scientific research without undermining their own competency. Lastly, from the academic scientist’s perspective, the project opens a completely new venue for deep and meaningful scientific interaction with remote communities while minimizing time and funding burdens.

Taken together, the benefits to all stakeholders underscore the potential of this approach to modernize and “democratize” science education by making geographical distance immaterial for participation. The project capitalizes on online tools that are freely available and utilizes open-source software that can be replicated and modified by any lab. Moreover, numerous laboratories are already equipped with robotic liquid handlers, and many research groups are incentivized by funding agencies to engage in scientific outreach. Thus, we hope that the proposed model for outreach can be widely replicated and encourage other scientific groups to adopt this model in order to reach numerous classrooms nationwide and worldwide. Moreover, our paradigm of experimentation by remote automation can be adopted to foster multiple similar-minded programs that actively engage numerous different audiences in cutting-edge scientific research.

## Supporting information

S1 Fig Analysis of the quality of student decision during the 2019 experiment by monitoring decision trees. (A) An example of a decision tree for a single well throughout the experiment. During the 2019 project, we reported to students the relative optical density (0–1 range) after 16 hours of growth in order to inform their decisions. Students used this daily reported information to decide on the drug regimen for the next day (choices were made out of 13 alternative treatments). The path shown by bold segments marks a set of decisions made over 9 days. In order to quantify the quality of students’ decisions, we defined a set of rules to determine whether a decision was scientifically “sensible” or “insensible” given the information the students had when they made the decision (optical density values and treatments up until that day). We categorized the following decisions as “insensible”: ramping up drug concentrations too quickly (e.g., exposing cells to high chloramphenicol concentrations without first exposing them to lower concentrations), increasing or maintaining the drug concertation despite observing only a slow growth (relative optical density was below 0.5), changing the drug type to an untested drug type after observing slow growth (relative optical density was below 0.5). Examples for “sensible” and “insensible” decisions are marked by green and red segments, respectively. (B) Changes in decision quality during the multiday experiment. We analyzed 92 decision trees and enumerated the numbers of “insensible” decision made every day. The analysis suggests that the number of “insensible” decisions overall decreased during the experiment.
(PNG)

S2 Fig Comparison between students’ and teachers’ questionnaires. (A) Students (*N* = 67) and teachers (*N* = 6) reporting on students’ cognitive and behavioral engagement. Bars mark mean score, and black segments mark the standard deviation (discrepancies between teachers and students were not statistically significant in a Kruskal-Wallis Test). (B) Students (*N* = 42–69) and teachers (*N* = 6) reporting on the value of different teaching and communication technologies. Bars mark mean score, and black segments mark the standard deviation (discrepancies between teachers and students were not statistically significant). (C) Teachers’ self-reported evaluation (*N* = 6) of their students’ achievements and project involvement. Each teacher was requested to evaluate the percentage of students that the different statements apply to (as compared to a standard curriculum taught over the years). The size of the pie slices indicates how many of the teachers had similar evaluations of their respective students.
(PNG)

S3 Fig The developed low-cost spectrophotometer. (A) We used an Arduino microcontroller and standard electrical components to build a circuit that can measure optical density in standard 2-ml tubes. The circuit was housed in small laser-cut acrylic box and 3D-printed plastic holder. The unit cost was less than $15 to fabricate. (B) A comparison between optical density measurements made by our low-cost spectrophotometer (using a red-light source, approximately 650-nm wavelength) and a commercial spectrophotometer (600-nm wavelength). Red segments are standard deviations. The comparison reveals a very high degree of correlation between the two spectrophotometers across the entire range of optical densities that is relevant for liquid bacterial cultures (0.05–2).
(PNG)

S1 Text Learning objectives. (DOCX)

S2 Text Lab lesson. (DOCX)

S3 Text Teacher guidelines. (DOCX)

S4 Text Daily protocol. (DOCX)

S5 Text Online setup. (DOCX)

S6 Text Equipment, time requirements, and scalability. (DOCX)

S7 Text Spectrophotometer parts and circuit. (DOCX)

S8 Text Spectrophotometer instructions. (DOCX)

S1 Code Matlab code. (DOCX)

S2 Code Spectrophotometer Arduino code. (DOCX)

S1 Design Acrylic box design. (PDF)

S2 Design Spectrophotometer tube holder. (STL)

S1 Presentation Lab evolution. (PPTX)

S2 Presentation Sequence alignment. (PPTX)
