# Supplementary figures and images for: Harnessing robotic automation and web-based technologies to modernize scientific outreach
Source: PLoS Biol. 2019 Jun 26;17(6):e3000348. doi: 10.1371/journal.pbio.3000348 (PMC6615640; doi:10.1371/journal.pbio.3000348)

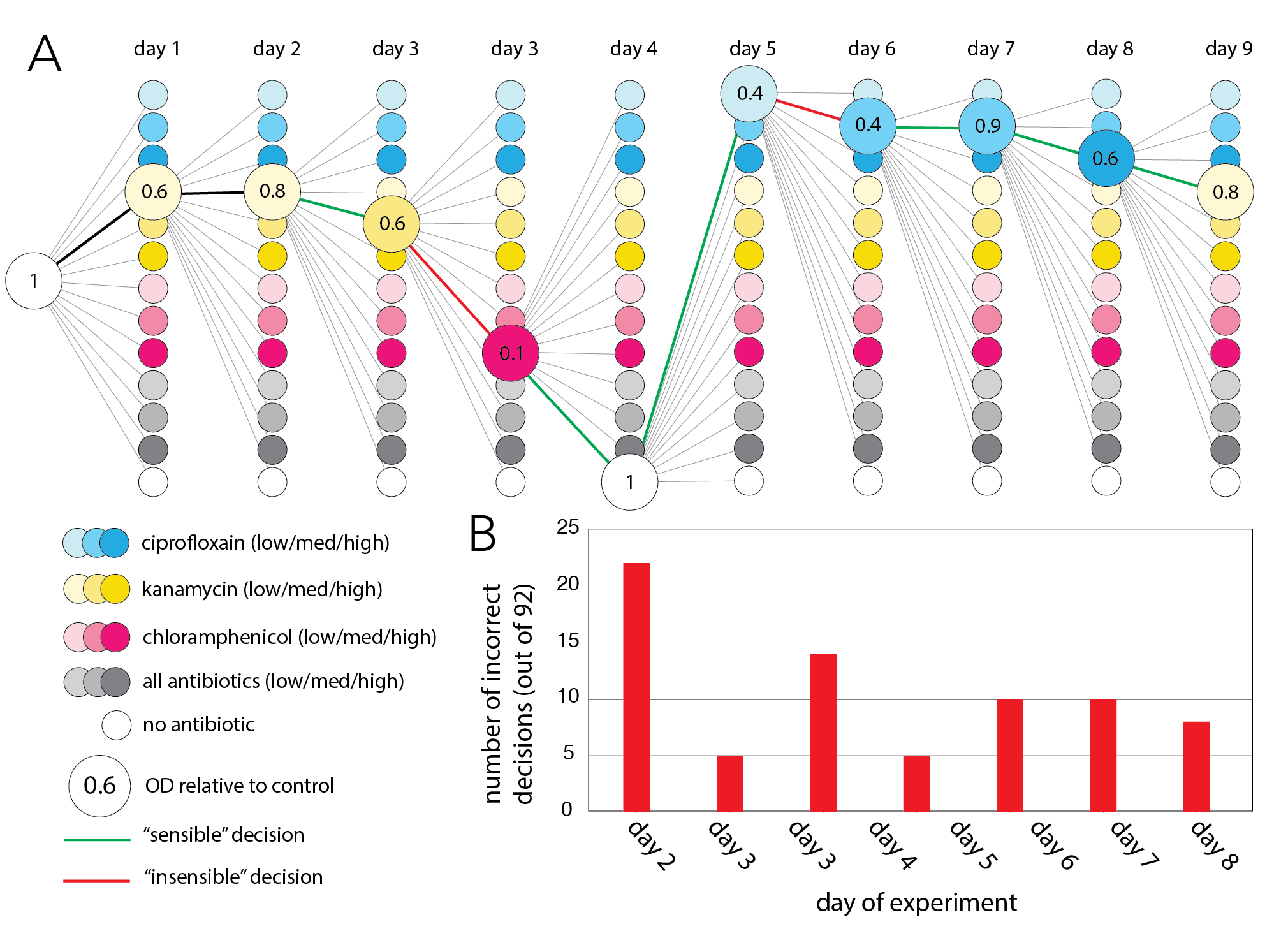

Supplement: S1 Fig — (A) An example of a decision tree for a single well throughout the experiment. During the 2019 project, we reported to students the relative optical density (0–1 range) after 16 hours of growth in order to inform their decisions. Students used this daily reported information to decide on the drug regimen for the next day (choices were made out of 13 alternative treatments). The path shown by bold segments marks a set of decisions made over 9 days. In order to quantify the quality of students’ decisions, we defined a set of rules to determine whether a decision was scientifically “sensible” or “insensible” given the information the students had when they made the decision (optical density values and treatments up until that day). We categorized the following decisions as “insensible”: ramping up drug concentrations too quickly (e.g., exposing cells to high chloramphenicol concentrations without first exposing them to lower concentrations), increasing or maintaining the drug concertation despite observing only a slow growth (relative optical density was below 0.5), changing the drug type to an untested drug type after observing slow growth (relative optical density was below 0.5). Examples for “sensible” and “insensible” decisions are marked by green and red segments, respectively. (B) Changes in decision quality during the multiday experiment. We analyzed 92 decision trees and enumerated the numbers of “insensible” decision made every day. The analysis suggests that the number of “insensible” decisions overall decreased during the experiment. (PNG) [file pbio.3000348.s001.png]

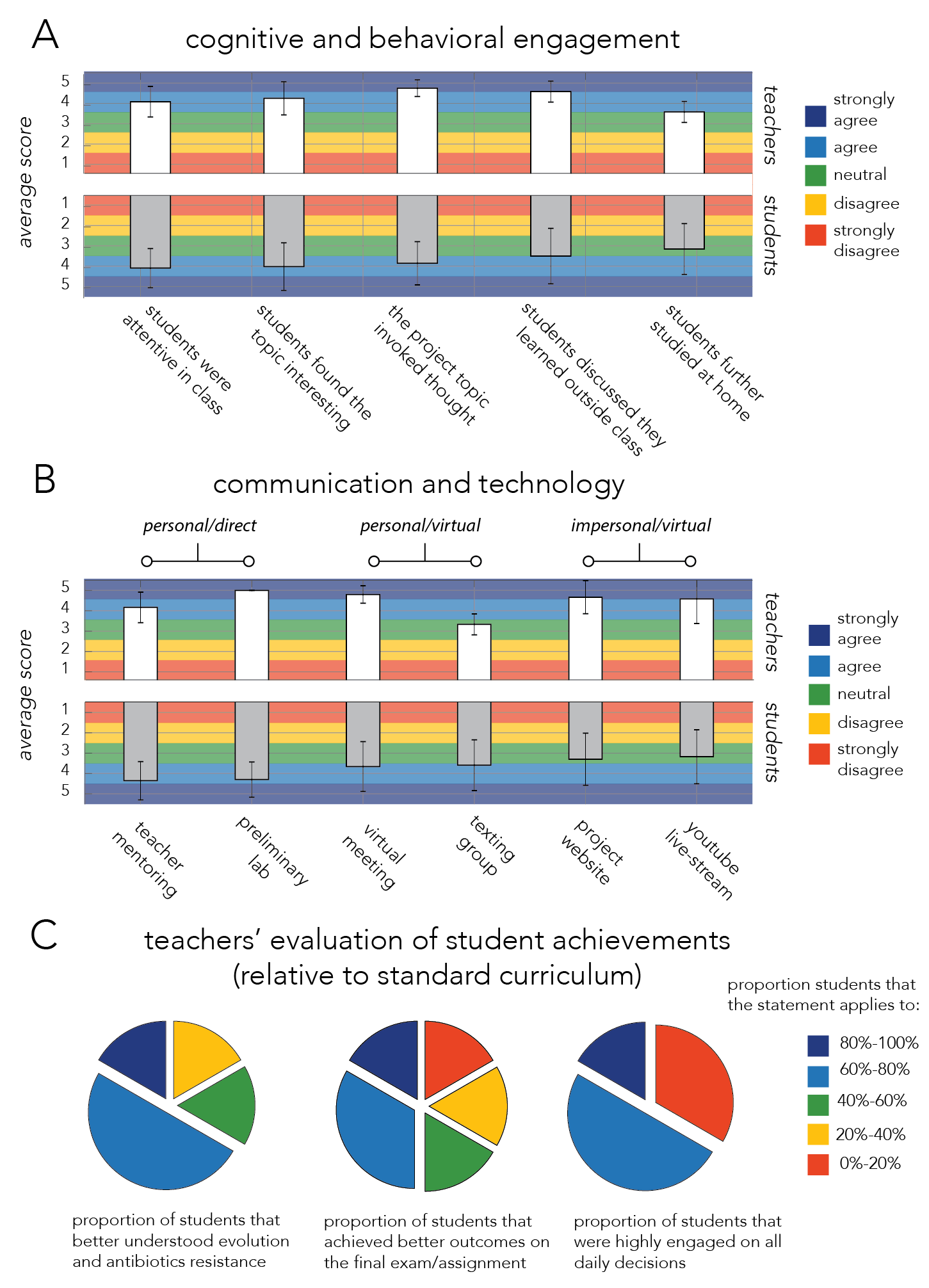

Supplement: S2 Fig — (A) Students (N = 67) and teachers (N = 6) reporting on students’ cognitive and behavioral engagement. Bars mark mean score, and black segments mark the standard deviation (discrepancies between teachers and students were not statistically significant in a Kruskal-Wallis Test). (B) Students (N = 42–69) and teachers (N = 6) reporting on the value of different teaching and communication technologies. Bars mark mean score, and black segments mark the standard deviation (discrepancies between teachers and students were not statistically significant). (C) Teachers’ self-reported evaluation (N = 6) of their students’ achievements and project involvement. Each teacher was requested to evaluate the percentage of students that the different statements apply to (as compared to a standard curriculum taught over the years). The size of the pie slices indicates how many of the teachers had similar evaluations of their respective students. (PNG) [file pbio.3000348.s002.png]

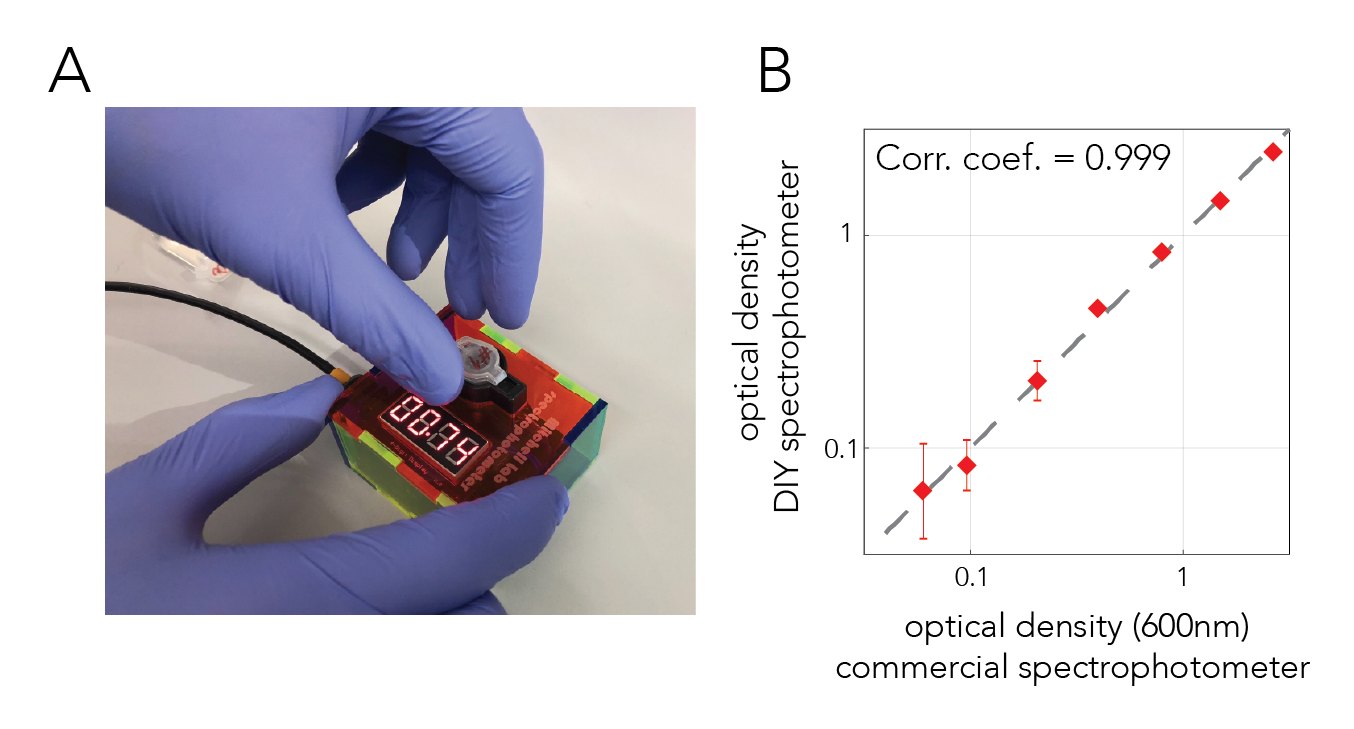

Supplement: S3 Fig — (A) We used an Arduino microcontroller and standard electrical components to build a circuit that can measure optical density in standard 2-ml tubes. The circuit was housed in small laser-cut acrylic box and 3D-printed plastic holder. The unit cost was less than $15 to fabricate. (B) A comparison between optical density measurements made by our low-cost spectrophotometer (using a red-light source, approximately 650-nm wavelength) and a commercial spectrophotometer (600-nm wavelength). Red segments are standard deviations. The comparison reveals a very high degree of correlation between the two spectrophotometers across the entire range of optical densities that is relevant for liquid bacterial cultures (0.05–2). (PNG) [file pbio.3000348.s003.png]

Cut parts from 1/8"  
thick acrylic sheet

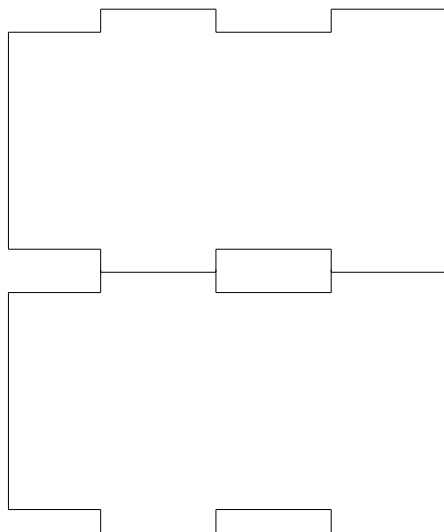

1 inch

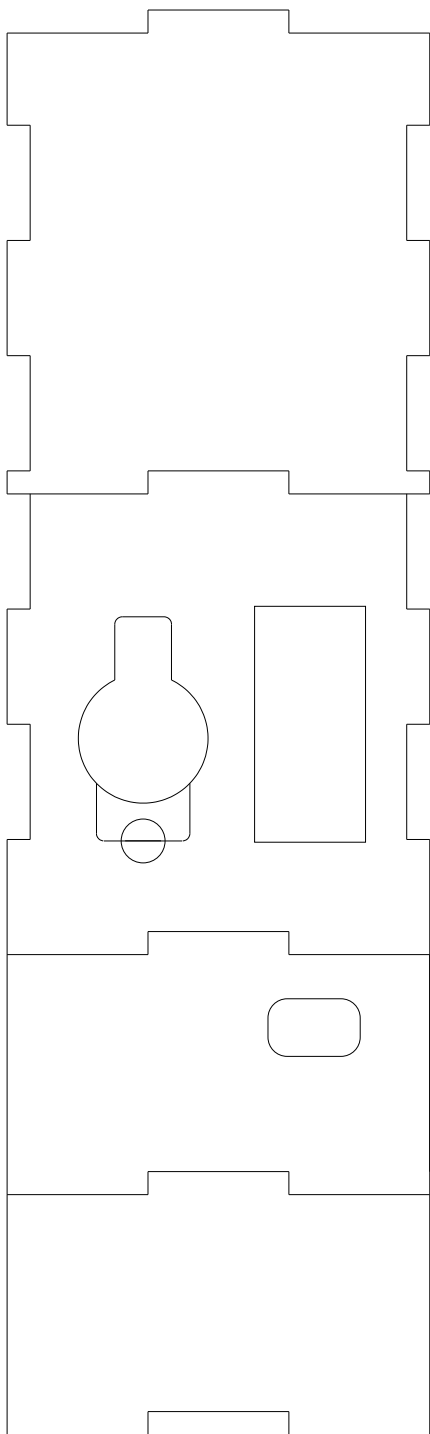

Supplement: S1 Design — (PDF) [file pbio.3000348.s014.pdf]
