# Supplementary material for: Harnessing robotic automation and web-based technologies to modernize scientific outreach
Source: PLoS Biol. 2019 Jun 26;17(6):e3000348. doi: 10.1371/journal.pbio.3000348 (PMC6615640; doi:10.1371/journal.pbio.3000348)
Supplement: S1 Text — (DOCX) [file pbio.3000348.s004.docx]

**Overall goals**

The overall goal is to provide students with an opportunity to engage in a cutting-edge research, in a highly relevant societal context:

- To instruct high-school students in performing a multi-day evolution experiment with the model bacteria *E. coli* in order to investigate the molecular mechanisms underlying evolved resistance against antibiotics over short evolutionary time-scales. Teach students
- To increase students’ enthusiasm and motivation for science learning, their awareness to the technologies used in research and their relevance to “real life”.

The project consists of three stages with individual specific goals:

1. The goal of stage 1 is to familiarize the students with the basics of bacterial growth and optical density, and with the effects of antibiotics of bacterial growth.
2. The goal of stage 2 is to engage students in a lab-evolution experiment in *E.coli*, to help them acquire relevant knowledge of evolution and current research practices.
3. The goal of stage 3 is to engage students in identifying mutations underlying evolved drug resistance, to help them understand the connection between mutations, evolution and emergence of multi-drug resistant pathogens.

**Learning objectives**

**Stage 1 – student preparation**

By the end of this stage students will be able to:

1. Measure optical density of liquid bacterial cultures with a spectrophotometer.
2. Plot and compare the growth curves of bacteria cultured in different antibiotics concentrations.
3. Infer the minimum inhibitory concentration of different antibiotics by growth curves.

**Stage 2 – evolution experiment**

By the end of this stage students will be able to:

1. Interpret how daily measurements of optical density can be used to infer on changes in drug resistance.
2. Propose selective drug regimens for a multi-day lab serial transfer experiment that lead to the evolutionary emergence of drug resistance.
3. Interpret systematic reports on drug resistance against a panel of multiple drugs.
4. Rationalize why drug resistance changes during a multi-day serial transfer experiment.

**Stage 3 – bioinformatics analysis of potential resistance mechanisms**

By the end of this stage students will be able to:

1. Discuss the rational for using targeted gene sequencing and whole-genome sequencing for identifying mutations underlying evolved drug resistance.
2. Use the online BLAST tool to execute a pairwise sequence alignment and interpret the generated report (identify mutations and infer their effect on the protein coding sequence).
3. Rationalize which of the identified mutations are likely to underlie antibiotic resistance by investigating the cellular mechanisms the mutations target.
