# Supplementary material for: Harnessing robotic automation and web-based technologies to modernize scientific outreach
Source: PLoS Biol. 2019 Jun 26;17(6):e3000348. doi: 10.1371/journal.pbio.3000348 (PMC6615640; doi:10.1371/journal.pbio.3000348)
Supplement: S2 Text — (DOCX) [file pbio.3000348.s005.docx]

**Lab lesson - Examining bacterial growth in the presence of antibiotic**

The aim of this experiment is to evaluate the effect of different antibiotic concentrations on the growth of *E. coli* cells.

**Reagent preparation (by the teacher)**

**a. Preparing the bacterial cultures**

1. Transfer a single colony of *E. coli* from an agar plate into 3 ml of sterile rich growth media (Luria Broth).
2. Grow the bacterial culture overnight in a 37^o^C incubator (in either a tube shaker or a tube roller).
3. Half an hour before the lab lesson begins, dilute the bacterial culture to Luria Broth in a ratio of 1:25 and return to incubator.

**b. Diluting the antibiotics**The antibiotics are provided as high concentration stocks and need to be diluted to working concentrations before adding them to the bacterial cultures. The recommended concentration range for different antibiotics (Kanamycin, Chloramphenicol and Ciprofloxacin) are provided on the project’s website.

1. Choose 5 different concentrations you wish to examine within the recommended range. An initial antibiotic solution should be made for each of the 5 chosen concentrations.
2. Prepare initial antibiotic solutions:

* These solutions will be further diluted in class. The antibiotics concentrations of these initial solutions should be ten-fold higher than the concentrations you chose to work with in the experiment itself.

* Make serial dilutions from stock in sterilized water to reach the desired antibiotics (X10) concentration. The final volume of each initial solution should be 1 ml.

1. Prepare the tubes for the students:

i. Transfer 200 ul of each antibiotic’s initial solution into a separate 2 ml tube using a pipette. Mark the antibiotic type and concentration on the cap of each tube.

ii. Prepare a control tube with 200 ul of sterile water using a pipette. Mark it as "control".

iii. Prepare a blank control tube with 200 ul of sterile water and 1.8 ml of rich growth media. Mark it as "blank control".

1. Keep the tubes in 4^o^C and pre-warm in a 37^o^C incubator before the experiment.

**Lab-lesson (suggested protocol for students)^[[1]](#footnote-1)^**

**a. Spectrophotometer preparation:**

1. Insert the "blank control" tube to the Spectrophotometer.
2. Connect the Spectrophotometer to the power-source and wait until the calibration step is finished (the LED display will flash multiple times).
3. Remove the blank control tube from the Spectrophotometer and re-insert it. Make sure the reading of optical density of the blank control tube is zero (if not, turn the Spectrophotometer off and repeat from step 1).

**b. Experiment protocol:**

1. Get all the bacterial cultures and the diluted antibiotics tubes from the incubator.
2. Add 1.8 ml of the bacterial cultures into each one of the tubes (except the "blank control" tube).
3. Close the tubes and mix by inverting them a few times.
4. Start a timer to keep track of the growth period.
5. Measure the optical density of each tube with the Spectrophotometer. Repeat measurements every 20 min over a period of two hours.
6. For each tube, write down in a table the measured optical density at each time point (minutes from the beginning of the experiment).

**Analysis of the results:**

1. Using Excel, produce a graphical representation showing the time on the x-axis and the measured optical density on the y-axis. The 5 concentrations should be represented as 5 curves on the same chart. If you tested several types of antibiotics, produce a separate chart for each type.
2. Review the results and determine the relationship between changes in optical densities and drug concentration.
3. Review the results and suggest the minimal inhibitory concentration (MIC) for each antibiotic.
4. Compare your results to other students' and to the results on the project website.

1. This is a suggested protocol with guidelines for the lesson. Teachers should adjust it to their class's level, previous lab experience and routines. [↑](#footnote-ref-1)
