# Supplementary material for: Harnessing robotic automation and web-based technologies to modernize scientific outreach
Source: PLoS Biol. 2019 Jun 26;17(6):e3000348. doi: 10.1371/journal.pbio.3000348 (PMC6615640; doi:10.1371/journal.pbio.3000348)

**Teacher guidelines**

The evolution experiment consists of 10 days of serial dilutions of bacterial cultures. Our robotic platform will dilute the culture daily into new media and we will monitor the growth after dilution. At the end of the experiment we will systematically measure the level of antibiotics resistance of all strains and will sequence specific genes by Sanger sequencing. In addition, we will identify super-bugs (strains that evolved the highest level of multi-drug resistance) and will sequence their **entire genome**.

Active participation in the multi-day evolution experiment requires participants to make informed decisions on a daily basis that are based on real-time observations made during the experiment. The participants can choose from 13 alternative treatments (3 alternative antibiotics, provided at three different concentrations, a combination of all three or no drug at all). Each class is designated 12 independent wells. We recommend dividing the students into smaller groups that will be responsible for 2-3 biological repeats of the same dosing regimen. When considering the daily decision strategy, we recommend considering the evolutionary question the group is addressing and the interim results from the previous day of the experiment. Moreover, as all results are shared in real time with all classes and schools, we recommend using the class time to review the interim results from other groups and discuss future decisions jointly. Following the discussion in class, it is recommended to allocate time for each group to determine their next step. They may consult with the teacher and/or the scientists (via the discussion forum), however, they should be given complete freedom to make their decisions. The discussions regarding the daily decision typically require only a few minutes with each group.

Please be aware of the following points when planning the experiments with your students:

- **Daily dilutions**: The media plates will be prepared an hour in advance according to the dosing regimen defined by the participating classes (teachers will update their shared google sheets). The drug regimens of **all** classes will appear on the daily results webpage. Our robotic platform will dilute the culture 1:100 at 10am (USA, EST).
- **Controls**: The first row is designated for joint controls that will be executed in three repeats. We will designate 3 control cultures without any drug and 9 control cultures on low concentrations of a single antibiotic for the entire evolution period.
- **Daily growth**: We will monitor the growth of the diluted cultures by measuring the optical density and calculating the generation time after 5h of growth. We will report the OD around 4pm (USA, EST). Use these results to decide in the drug regimen for the next day.
  ***Note****: if the OD is below 10% of the no-drug control, the bacterial culture might not recover enough. We recommend growing it without any antibiotics on the next day. We also recommend not increasing the antibiotics concentration if the OD is above 50% relative to the no-drug control.*
- **Live stream and social media**: Live-stream video from the robotic platform will appear on the daily feed webpage. We will also post regular updates on the text group.
- **Final results**: At the end of the experiment will systematically test if the evolved strains become drug resistant by measuring their MIC (minimal inhibitory concentration) on all antibiotics. Improved drug resistance is defined by a higher MIC compared to the ancestor strain. Multi-drug resistance is defined by higher MIC on all antibiotics.
- **Sanger sequencing**: In order to gain insight in the molecular mechanism of drug resistance we will sequence two genes by Sanger sequencing. Each class will choose a few evolved strains for monitoring mutations in [gryA](http://www.uniprot.org/uniprot/P0AES4) (associated with resistance to ciprofloxacin) and in [fusA](http://www.uniprot.org/uniprot/P0A6M8) (associated with resistance to kanamycin).
- **Full genome sequencing**: We will fully sequence the genome of most resistant super-bug (the strain with highest MIC on all drugs).

**Evolutionary questions to explore**

Each participating class will decide on the scientific question it is interested in exploring. A good practice is to use 2-3 repeats of each of the growth conditions. Here are a few suggestions for possible questions:

1. **What is the fastest route to drug resistance?** compare the resistance of bacteria evolving on a single antibiotics concentration to that of bacteria evolving on gradually increasing concentrations.
2. **Do drug "holidays" matter?** compare the resistance of bacteria evolving on constant antibiotics to that of bacteria single with a day with antibiotics and a day without antibiotics.
3. **How does multi-drug resistance emerge?** compare the resistance of bacteria evolving when all drugs administered simultaneously every day to that of bacteria that evolved on alternating antibiotics (a cycle of singe antibiotic every day).
4. **Does cross resistance evolve naturally? Does cross sensitivity evolve naturally?** compare the MIC pattern of strains evolving only on a single antibiotics and test if evolved strains become more or less resistant to antibiotics they never encountered.


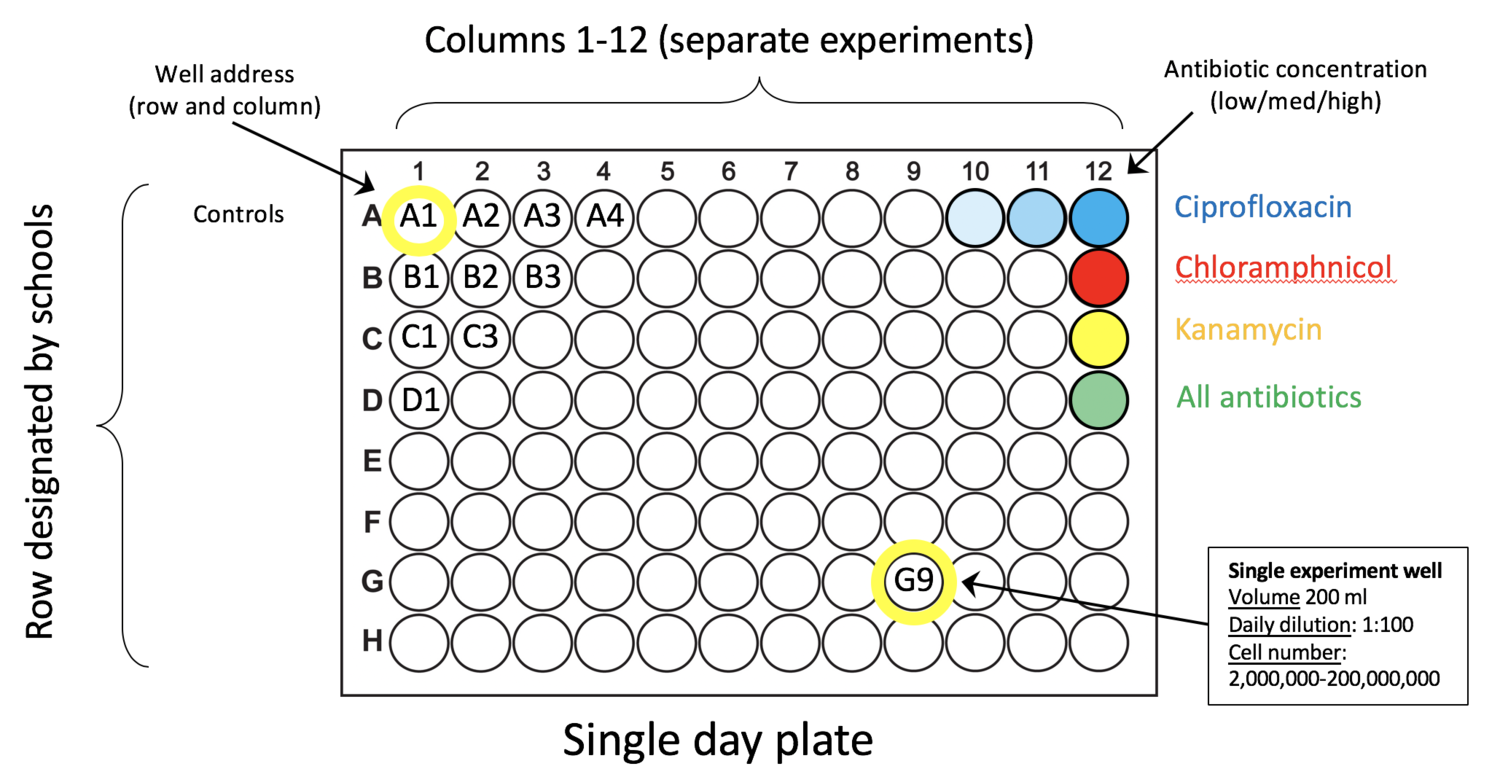

Supplement: S3 Text — (DOCX) [file pbio.3000348.s006.docx]
