# Supplementary material for: Harnessing robotic automation and web-based technologies to modernize scientific outreach
Source: PLoS Biol. 2019 Jun 26;17(6):e3000348. doi: 10.1371/journal.pbio.3000348 (PMC6615640; doi:10.1371/journal.pbio.3000348)
Supplement: S4 Text — (DOCX) [file pbio.3000348.s007.docx]

**Media and strains**

We used *Escherichia coli* MG1655 for all experiments. M9 minimal medium supplemented with 0.4% glucose and 0.2% amicase was used to prepare media with antibiotics. We prepared stock solutions of the following antibiotics and kept at -20 C: Kanamycin Monosulfate (Alfa Aesar, Reference: J6066803) as 50 mg/ml in sterile pure water, Ciprofloxacin (Alfa Aesar, Reference: J6131706) as 25 mg/ml in 0.1N HCl and Chloramhenicol (Acros Organics, Reference:227920250) as 50 mg/ml in Ethanol. We used McCormick Assorted food color & egg dyes (Reference: 52100071077) to prepare a master stock by diluting the dye 1:180 into M9 medium. For evolution experiments, we diluted the master stocks into the media already supplemented with antibiotics as follows (Blue: 1:6000, 1:2000, 1:1000, Red: 1:3000, 1:1500, 1:375, Yellow: 1:3000, 1:1500, 1:750). We validated in control experiments that the dyes do not affect bacterial growth at the concentration used.

**Daily protocol**

The information provided here is for an epMotion (Eppendorf) liquid handler. The computer scripts, provided as open source, can be readily modified to match the syntax of other robotic liquid handlers. Alternatively, the same operations can be done manually.

1. Media preparation: Dilute antibiotics (ciprofloxacin, chloramphenicol, and kanamycin) into 30 ml M9 media at their final concentrations. Supplement the media with diluted food dyes to allow visual identification of antibiotic type and concentration. Keep the antibiotic solutions at 4C when not in use.
2. Generate the liquid handler instructions using the google2csv.m Matlab script. The script uses information provided in online Google sheets to generate a CSV instruction file (for operation of the epMotion robot). The script also generates images that show how the media will be allocated in the 96-well plate (plate map) and additional images that shows the media rack organization (Daily Protocol Figure A). Share the plate map with remote users.
3. Prepare the liquid handler for operation. Place media racks according to images generated by the google2csv.m Matlab script.
4. Start the YouTube live stream (information on live-streaming can be found on the YouTube help: <https://support.google.com/youtube/topic/9257891?hl=en&ref_topic=9257610>). Share the live-stream link with remote users.
5. Turn on the the epMotion liquid handler. Start its operation with a blank program and upload the CSV file generated on (2). The liquid handler will prepare the plate according to remote user specifications.
6. Place a new 96-well plate on the position C1 and remove the lid (Daily Protocol Figure B, Stage I). Run an epMotion program that transfers 198 ul of media with various antibiotics according to the students’ choices.
7. Next, place the 96-well plate from the previous day on the epMotion stage position C2 and remove the lid (Daily Protocol Figure B, Stage II). Also place the necessary equipment on the epMotion stage according to the Stage II (Figure 2B right). Run the epMotion program that transfers 2ul from the previous day plate to the newly prepared plate. (Note that this program washes the tips at position B2 and returns them to empty tip box in position B1)
8. Stop the YouTube live stream. YouTube automatically saves this movie in your Youtube account. The movie can be edited and annotated for future viewers.
9. Place the newly inoculated plate in the microplate spectrophotometer (Spark/TECAN). Use a kinetic cycle for shaking and incubation (37^0^C) to measure optical density (600nm) every 10 min for 6 hours (or longer). Export the optical density reading to an excel file.
10. Use the Matlab script inferDoublingTime.m to infer the generation time during exponential growth. The script generates an image of the generation time in each well. Share this result with remote users. Alternatively, you can use determineDailyOD.m script to plot the growth curves and final optical density for the same data set.
11. Visually inspect the plate well before the next culture dilution in order to identify contamination. Hallmarks of contamination include changes culture color or appearance (e.g., sedimentation on bacterial clumps). It is also possible to include a well without any bacteria in each experiment as an additional control. If potential contamination is detected, evaluate it by microscopy imaging and by streaking isolated colonies on agar plates. If contamination is detected, repeat the last day experiment with previous-day plates stored in the fridge.
12. After daily plate inoculation (step 6), store the previous day plate in fridge (4^o^C).


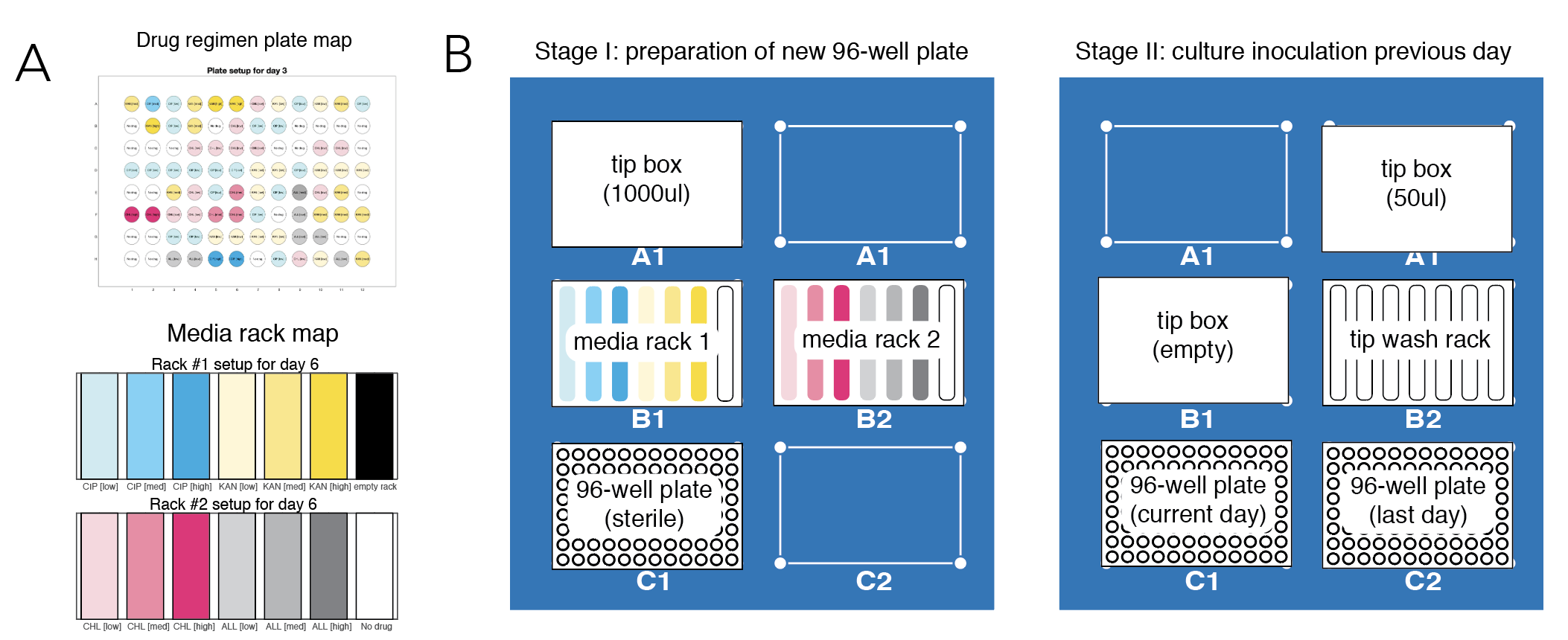


**Daily protocol**. (A) Sample output files from the google2csv.m script showing the desired drug regimen requested by remote users and the organization of the media racks. (B) The files we provide require the following organization of labware on the liquid handler stage.
