# Supplementary material for: Harnessing robotic automation and web-based technologies to modernize scientific outreach
Source: PLoS Biol. 2019 Jun 26;17(6):e3000348. doi: 10.1371/journal.pbio.3000348 (PMC6615640; doi:10.1371/journal.pbio.3000348)
Supplement: S5 Text — (DOCX) [file pbio.3000348.s008.docx]

**Google sheets setup**

Multiple Google sheets can be used to pool together requests from remote users into a central sheet. The setup is meant to provide remote users full editing privileges for their own sheet and only viewing privileges for the pooled sheet. The google template we provide bellow allows remote users to choose the drug regimen (type/concentration) from a pull-down menu. User sheets are organized by row (A-H) in a 96-well plate. Google sheets can be embedded into any website and they are automatically updated by Google once a user edits them through Google drive (thus allowing to monitor remote user choices in real-time). More information on editing and sharing google sheet can be found here:

<https://gsuite.google.com/learning-center/products/sheets/get-started/#!/>

Templates of the google forms that we created can be found here:

- Remote user google Form:
  <https://docs.google.com/spreadsheets/d/1I6AgdqpyN6Q6rhKV6QE9OcbTp4J9qeIgw9oCNTghOyA/edit?usp=sharing>
- Multi-tab Pooled google Form (tabs correspond rows A-H):
  <https://docs.google.com/spreadsheets/d/1QnEExjGoNgpk0QGvslnvGfT5aXgPKDzaBnTohogaRUA/edit?usp=sharing>


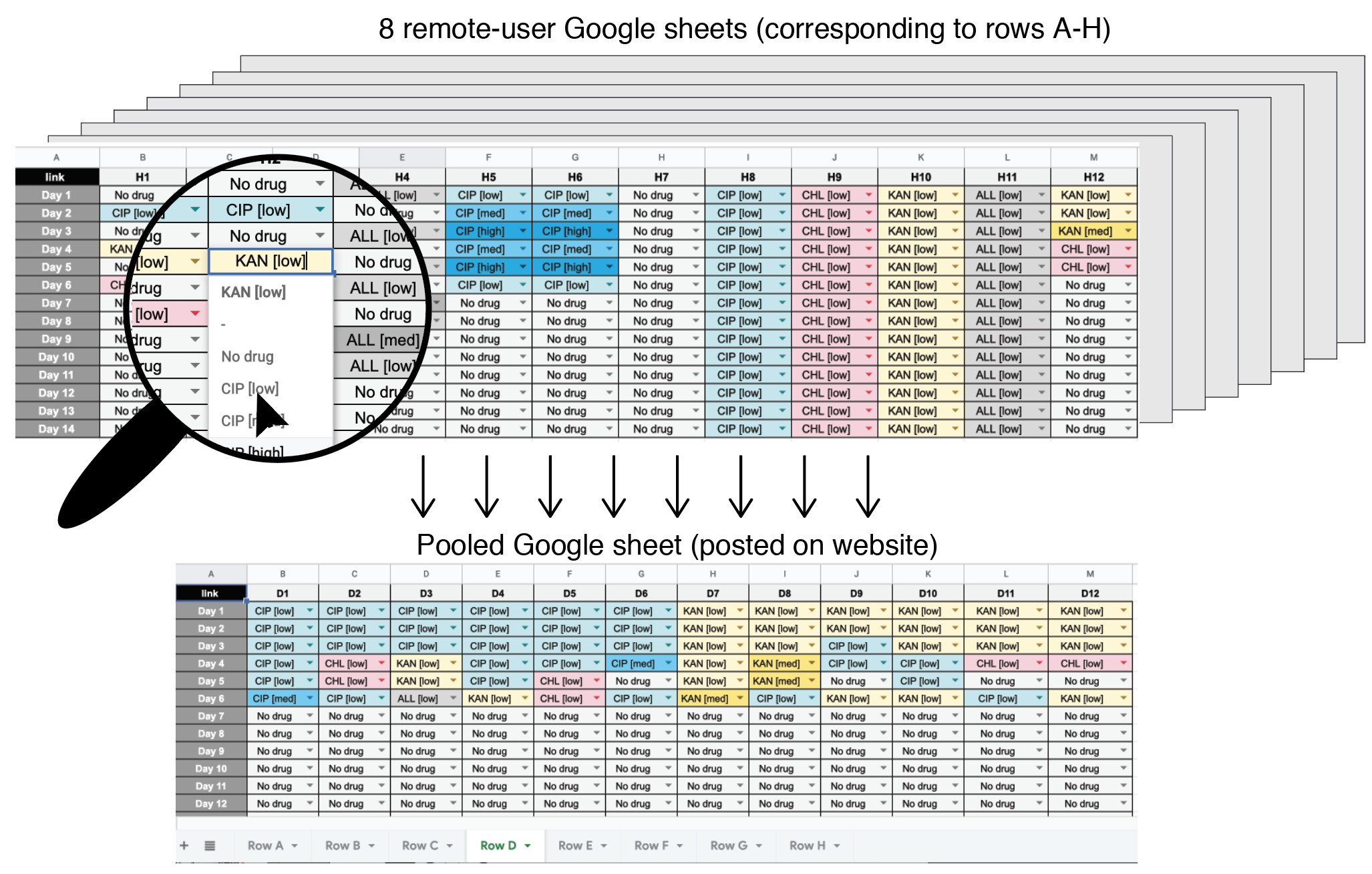


**Figure 1 –Information flow diagram for Google sheets.** The lab sets up Google sheets that mediate the drug regimen choices. Each sheet corresponds to a single row of wells on the 96-well plate. The Sheets are shared with remote classes with full editing privileges. The information from the 8 sheets is pooled together automatically and is used to populate a multi-tab pooled google sheet that can be embedded into any website. This design allows remote users to edit and modify only the wells designated to them while also allowing to view the choices made by all other participants. The remote user sheets allow choosing the drug regimen from a drop-down menu in every table cell (shown by magnified area)

**YouTube setup**

YouTube allows any user to live-stream video content. We used live-stream to broadcast the daily operation of the liquid handler. In order to simultaneously stream from two cameras and add additional texts, we used the *Open Broadcaster Software,* a free and open source software for video recording and live streaming (<https://obsproject.com>). Once live-stream has ended YouTube automatically saved the captured media. The saved movie can be edited and uploaded to YouTube for public viewing. The movies we created during the 2018 project can be found in this YouTube playlist:
<https://www.youtube.com/playlist?list=PLKuPiM_e383pkHbg5PwBdMrJNPzUu0zEt>

Additional information on live-streaming can be found on the YouTube help: <https://support.google.com/youtube/topic/9257891?hl=en&ref_topic=9257610>).
