# Supplementary material for: Harnessing robotic automation and web-based technologies to modernize scientific outreach
Source: PLoS Biol. 2019 Jun 26;17(6):e3000348. doi: 10.1371/journal.pbio.3000348 (PMC6615640; doi:10.1371/journal.pbio.3000348)
Supplement: S6 Text — (DOCX) [file pbio.3000348.s009.docx]

**Equipment and consumables - Research lab**

Equipment

Robotic liquid handler (Eppendorf/epMotion M5073c or any alternative)

Micro-plate spectrophotometer (Tecan/Spark or any alternative)

Standard desktop computer (Apple, iMac or any alternative)

Plate shaker/incubator (Heidolph/Inkubator 1000, Titramax 1000 or any alternative)

Standard web-camera

Standard micropipettes (P1000, P200, P10)

Consumables and reagents:

96-well plates (Eppendorf/Cat #0030730119) – 36 units

Liquid handler 50uL tips (Eppendorf/Cat # 0030014430) – 13 tip boxes

Liquid handler 1000uL tips (Eppendorf/Cat # 0030014430) – 1 tip box

Liquid handler 30 ml reservoirs (Eppendorf/Cat # 960051009)

Liquid media for bacterial growth (M9 with 0.4% glucose and 0.2% amicase) – 1 liter

Antibiotics (Ciprofloxacin, Kanamycin, Chloramhenicol)

Taq DNA polymerase (New England BioLabs/Cat #M0273)

Food Dyes (McCormick Assorted Food & Egg Dye, Reference: 52100071077 or any alternative)

70% Ethanol

**Equipment list – High-school classrooms**

Equipment

Incubator and shaker

Spectrophotometer (Mitchell lab, DIY spectrophotometer)

Standard micropipettes (P1000, P200)

Consumables and reagents:

Liquid media for bacterial growth (Luria broth) – 1 liter

Standard serological pipettes for handling media.

Standard 50mL tubes for culture growth and dilution (50mL tubes)

Standard 2mL transparent tubes for optical density measurement – 50 tubes

Antibiotics

**Estimation of time requirements for the online evolution experiment – Research lab**

Estimations do not include time required for initial setup of project website, Google sheets, and YouTube broadcast.

Initial preparation (20 man-hours)

Initial Reagent preparation (antibiotics stocks, food-dyes, media) – 2 man-hours

Quantifying drug sensitivity of ancestor strain - 6 man-hours

Teacher preparation (online demo experiment, online Q&A session) – 6 man-hours

Resetting of online tools (Google sheets, project website, discussion forum) – 6 man-hours

Daily time requirements (2 man-hours)

Preparing the Liquid handler (reagent preparation, stage setting) – 0.25 man-hours

Downloading and processing requests of remote users – 0.25 man-hours

Liquid handler operation and cleanup – 0.75 man-hours

Processing and uploading daily results – 0.25 man-hours

Daily interactions in discussion forums - 0.5 man-hours

Online events (3 man-hours)

Virtual Q&A sessions (3 sessions) – 1.5 man-hours

Virtual lab visits (3 visits) – 1.5 man-hours

Concluding experiments (15 man-hours)

Quantifying drug sensitivity of evolved strains on three antibiotics – 8 man-hours

Targeted sequencing of selected genes (40 reactions) – 4 man-hours

Whole genome sequencing by external service (DNA extraction, Bioinformatics) – 3 man-hours

**Scaling-up the online evolution experiment**

We ran the outreach project twice. Once with 162 students (7 classes) and once with 88 students (6 classes). We found the project works well with medium sized classes (12-18 students) that are subdivided into smaller working groups of 2-3 students. Each group is then put in charge of 2-3 repeated experiments (in 2-3 individual wells). The scaling-up strategies proposed below will likely work best for engaging additional classes in the online evolution experiment. Our experiences led us to believe that scaling up the number of students per class is suboptimal since the students-to-teacher ratio would be too high for a proper evaluation of the daily interim results. We also do not advise scaling up the number of wells designated to a single class since monitoring more than 12 experiments on a daily basis becomes overwhelming for both students and teachers.

Scaling-up of the described system, with minimal modifications, can be achieved by using a 384-well plate or by running multiple 96-well plates sequentially for the evolution experiment:

1. **Scaling up by reducing working volume** – typical robotic liquid handlers and microplate spectrophotometers are compatible with 384-well plates. Thus, a very similar experiment can be run in a 384-well plate format by reducing the working volumes to 40 uL.
2. **Scaling up by staggering experiments over time** – daily operation of the liquid handler which aliquots drugs and inoculates the bacteria requires half an hour. Thus, multiple independent plates can be run sequentially over multiple hours. Most micro-plate spectrophotometers allow incubating and monitoring the optical density of only one plate at a time. This creates a measurement bottleneck which can be circumvented by incubating all plates in a standard shaker and measuring the optical density only once (e.g., after over-night growth). Such single time-point information is sufficient for inferring degree of drug resistance and making informed decisions on the next-day drug regimen.
