# Supplementary material for: Harnessing robotic automation and web-based technologies to modernize scientific outreach
Source: PLoS Biol. 2019 Jun 26;17(6):e3000348. doi: 10.1371/journal.pbio.3000348 (PMC6615640; doi:10.1371/journal.pbio.3000348)
Supplement: S7 Text — (DOCX) [file pbio.3000348.s010.docx]

**DIY Spectrophotometer / Mitchell-Lab- Spectrophotometer (MLS)**

**Part List:**

Arduino Nano board with pin header (Part #: A000005)

A male to mini-B USB cable

4 Digits LED Display with pin header (Part #: TM1637)

Analog Light Sensor with pin header (Part #: GA1A12S202)

5mm Standard Diffused Red LED
Light Sensor (Part #: GA1A12S202)
Resistor (4.7k ohm)

12+ - Female-female jumper cables and 22AWG Solid Core wires

2 ml clear tubes for sample reading

3D-printed tube holder

Laser cut acrylic case (1/8” acrylic sheet)

Water thin solvent acrylic cement (Part #: CECOMINOD039446)

Lead-free soldering Iron and solder for pin heads

**Circuit diagram:**

**
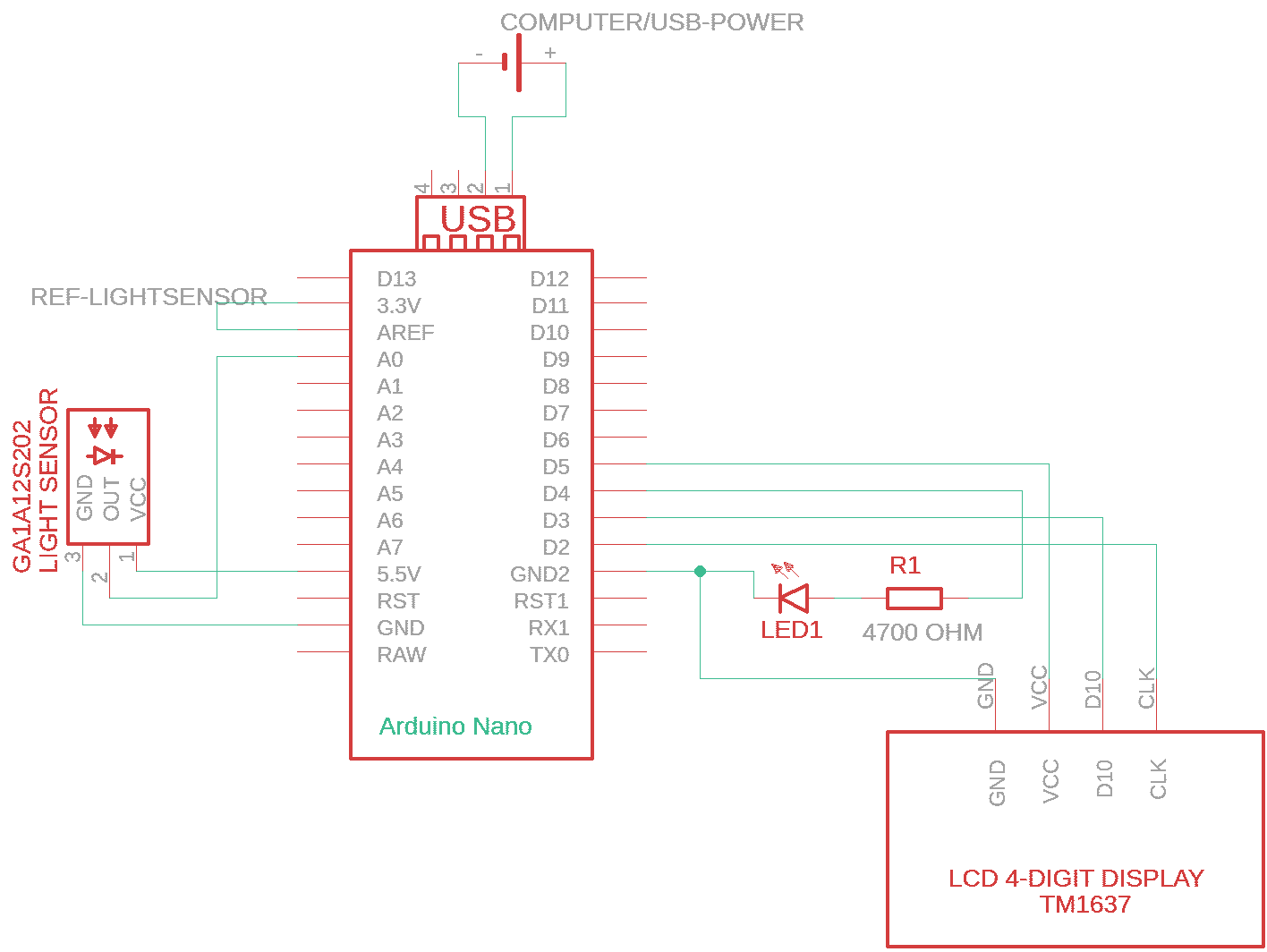
**

**3-D printed holder**

**Alternative designs for open-source spectrophotometers**<https://github.com/Hackuarium/simple-spectro>
<https://publiclab.org/wiki/spectrometry>
