# Supplementary material for: Harnessing robotic automation and web-based technologies to modernize scientific outreach
Source: PLoS Biol. 2019 Jun 26;17(6):e3000348. doi: 10.1371/journal.pbio.3000348 (PMC6615640; doi:10.1371/journal.pbio.3000348)
Supplement: S8 Text — (DOCX) [file pbio.3000348.s011.docx]

**Mitchell lab spectrophotometer (MLS) – user instructions**

**Briefly: the MLS evaluates the optical density of cell cultures by sensing the changes in light intensity as a red-light beam passes through a standard 2mL plastic tube. The best accuracy (±5%) is in the OD range of 1-0.0.5.**

***Caution*** *- The MLS is designed for use only in high-school classrooms under the supervision of a trained teacher. Small parts may break off and are a choking hazard for small children.*

1. **Blank calibration** – OD measurements are relative to a blank measurement (a tube that contain only media). The blank calibration is preformed immediately upon startup.
2. **Before** powering on, place a blank tube (tube with 2mL media) in the tube holder.
3. Power the MLS by connecting the USB cable to a computer or a power source.
4. The calibration is done once the display blinks. Keep power ON during the whole experiment (over multiple hours).
5. **OD measurement** – place a 2mL tube containing the cell-culture (containing at least 1.5mL) in the tube holder in the correct orientation. The OD measurement is done within a few seconds.

**Tips**

1. Use 2mL tubes with at least 1.5mL liquid media (the light beam crosses the tube just below the 1.5mL line).
2. The MLS has no buttons and turns ON when connected to a USB. It measures OD continuously.
3. Make sure the tube is clean and inserted in the correct orientation (any writing or dirt on the side of the tube will interfere with the light path and reduce measurement accuracy)
4. The MLS is extremely fragile and can easily break. Avoid dropping it or moving its electrical components.

| 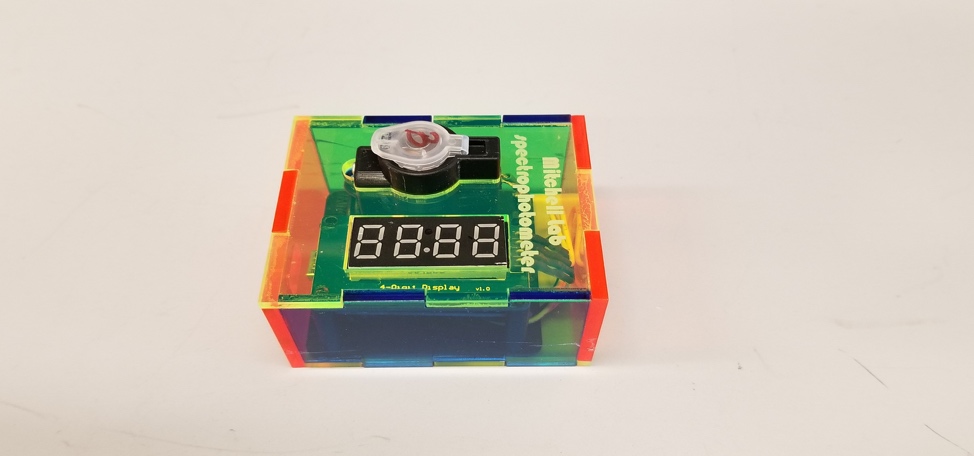 | 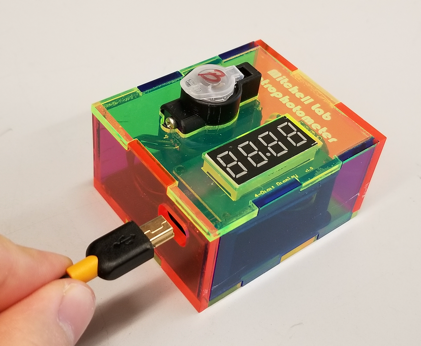 | 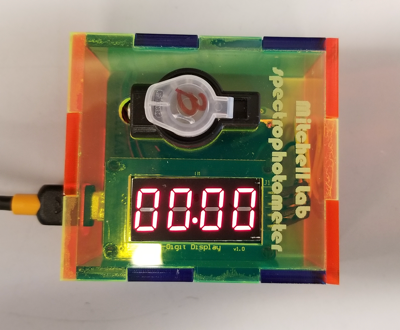 | 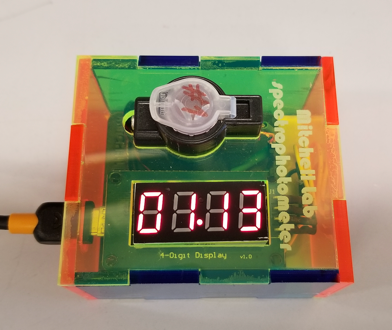 |
| --- | --- | --- | --- |
| Insert the blank tube  (in **correct orientation**) | Connect to usb cable to supply power | Wait for calibration  (display will flash) | Insert culture tube. keep the reader on throughout |
