# Supplementary material for: Harnessing robotic automation and web-based technologies to modernize scientific outreach
Source: PLoS Biol. 2019 Jun 26;17(6):e3000348. doi: 10.1371/journal.pbio.3000348 (PMC6615640; doi:10.1371/journal.pbio.3000348)
Supplement: S1 Code — (DOCX) [file pbio.3000348.s012.docx]

# README.txt

The following matlab scripts were used for the outreach project:

inferDoublingTime.m - a script for inferring the generation time from time-lapse measurements of a microplate spectrophotometer (used for the 2018 project)

determineDailyOD.m - a script for plotting the growth curve and final OD reads time from time-lapse measurements of a microplate spectrophotometer (used for the 2019 project)

loadLookupTable.m - a matlab script the constructs a lookup table (cell array) that maps drug to labware/source/color code

google2csv.m - a matlab script for downloading data from a shared google sheet and generating csv file

getLabwareSource.m - utility function to find a value in the lookup table

myGetGoogleSpreadsheet.m - a modified version of GetGoogleSpreadsheet.m script (available at the matlab file exchange). The modification allows accessing multiple tabs (sheets) within the same document. The modifications are:

1. Request a second input argument. Change the function header

from: function result = myGetGoogleSpreadsheet(DOCID)

to: function result = myGetGoogleSpreadsheet(DOCID,gid)

2. The URL request line was modified to allow accessing a specific tab from

a multi tab google sheet. Make the following change in the function code

from : csvURL = ['https://docs.google.com/spreadsheet/ccc?key=' DOCID '&output=csv&pref=2'];

to csvURL = ['https://docs.google.com/spreadsheet/ccc?key=' DOCID '&output=csv&pref=2&gid=' gid];

%% inferDoublingTime.m

%% this calculate the OD reading from the first 5h to calculate the doubling time

%% DEFINITIONS

strFileName = 'myFile.xlsx'; strRange = 'D52:CU87';

timeInterval = 10/60; % time interval between measurements (in hours).

rowNumber = 8; colNumber = 12;

%% extract the OD the data from excel files

rawData=xlsread(strFileName, strRange);

n = size(rawData,1);

time=[0:timeInterval:timeInterval*(n-1)]; % calculate time array by interval

%% substract the blank reading (& dye background)

OD0 = median(rawData([1:3],:),1); % treat the first 3 time points as blanks

normOD = rawData-OD0;

%% convert dataset to a 8x12 cell array

raw8x12 = cell(rowNumber,colNumber);

norm8x12 = cell(rowNumber,colNumber);

i = 1;

for r=1:rowNumber;

for c=1:colNumber;

curRawOD = rawData(:,i);

curNormOD = normOD(:,i);

raw8x12{r,c} = curRawOD;

norm8x12{r,c} = curNormOD;

i = i+1;

end

end

%% plot the raw and normalized OD graphs

figure('color','w');

i = 1;

maxOD = max(max(rawData));

minOD = min(min(rawData));

for r=1:rowNumber;

for c=1:colNumber;

subplot(rowNumber,colNumber,i); hold on;

plot(time,raw8x12{r,c},'k');

plot(time,norm8x12{r,c},'r');

set(gca,'ylim',[0 maxOD],'xlim',[min(time) max(time)]); box on;

i = i+1;

end

end

set(gcf,'position',[395 186 1132 762]);

suptitle('Growth curves');

savefig(['Growth curves for day - x' '.fig']);

%% claculate the growth rate and doubling times (using linear fit to log(OD))

grArray = nan(n,1); gr8x12 = nan(rowNumber,colNumber);

taoArray = nan(n,1); tao8x12 = nan(rowNumber,colNumber);

annotationPlate = cell(8,12);

rmse = [];

i = 1;

x = time';

for r=1:rowNumber;

for c=1:colNumber

y = norm8x12{r,c};

inx = find([1<x & y>0.005 & y<0.25]); % use OD measurements only from the log range

if(length(inx)>3)

[f gof] = fit(x(inx),log(y(inx)),'poly1');

gr = f.p1;

rmse = gof.rmse;

else

gr = 0;

rmse = inf;

end

%figure; hold on; plot(x,log(y),'.'); plot(x,f(x));

curTao = log(2)/gr*60; % generation time in minutes

maxTao = 240; % max tao to report

if(curTao<maxTao & gof.rmse < 0.5 & curTao>0)

taoArray(i) = curTao;

tao8x12(r,c) = curTao;

grArray(i) = gr;

gr8x12(r,c) = gr;

annotationPlate{r,c}=num2str(round(curTao));

else

taoArray(i) = nan;

tao8x12(r,c) = nan;

grArray(i) = 0;

gr8x12(r,c) = 0;

annotationPlate{r,c}='No';

end

i = i+1;

end

end

%% plot the generation times

figure; hold on;

microplateplot(tao8x12,'MissingValueColor',[1.0000 0 1.0000],'TextLabels',annotationPlate,'textFontSize',14);

colormap(cool);

title('Generation time (min)','fontsize',24);

set(gcf,'position',[395 186 1132 762]);

savefig(['Generation time for day - x' '.fig']);

%% determineDailyOD.m

%% This code calculates and plots the final OD and the growth curve after 16h of growth

%% DEFINITIONS

strFileName = 'myFile.xlsx'; strRange = 'D29:CU149';

timeInterval = 10/60; % time interval between measurements (in min)

isByCol = true; % orientation of wells in measurements (read by column or rows)

rowNumber = 8; colNumber = 12;

%% request user input (which day to process)

prompt = {[ 'Day of experiment for ' strFileName]};

dlg_title = 'Plot OD at 16h ';

num_lines = 1;

defaultans = {'0'};

userInput = inputdlg(prompt,dlg_title,num_lines,defaultans);

iDay = str2num(userInput{1});

%% extract the OD data from excel file

rawData=xlsread(strFileName, strRange);

n = size(rawData,1);

time=[0:timeInterval:timeInterval*(n-1)]; % calculate time array by interval

if(~isByCol) % need to re-orient the dataset (in case OD are reported by rows)

junk = rawData;

rawData=[junk(:,1:8:end) junk(:,2:8:end) junk(:,3:8:end) junk(:,4:8:end) junk(:,5:8:end) junk(:,6:8:end) junk(:,7:8:end) junk(:,8:8:end)];

end

%% subtract the t0 reading (dye and media background)

OD0 = median(rawData([1:3],:),1); % treat the first 3 time points as blanks

normOD = rawData-OD0;

%% convert dataset to an 8x12 cell array

raw8x12 = cell(rowNumber,colNumber);

norm8x12 = cell(rowNumber,colNumber);

finalOD8x12 = nan(rowNumber,colNumber);

annotationPlate = cell(rowNumber,colNumber);

i = 1;

for r=1:rowNumber;

for c=1:colNumber;

curRawOD = rawData(:,i);

curNormOD = normOD(:,i);

raw8x12{r,c} = curRawOD;

norm8x12{r,c} = curNormOD;

finalOD8x12(r,c) = norm8x12{r,c}(end);

annotationPlate{r,c} = num2str(finalOD8x12(r,c),2);

i = i+1;

end

end

%% plot the raw and norm OD graphs

h = figure('color','w');

i = 1;

maxOD = max(max(rawData));

minOD = min(min(rawData));

for r=1:rowNumber;

for c=1:colNumber;

subplot(rowNumber,colNumber,i); hold on;

%plot(time,raw8x12{r,c},'k');

plot(time,smooth(norm8x12{r,c}),'r'); grid on;

set(gca,'ylim',[0 maxOD],'xlim',[min(time) max(time)],'xtick',[0:5:15]); box on;

i = i+1;

end

end

set(gcf,'position',[395 186 1132 762]);

suptitle(['Growth curves - Day ' num2str(iDay)]);

savefig(['Growth curves (16hr) - Day ' num2str(iDay) '.fig']);

saveas(h,['Growth curves (16hr) - Day ' num2str(iDay) '.jpg']);

%% plot the final OD

figure; hold on;

microplateplot(finalOD8x12,'MissingValueColor',[1.0000 0 1.0000],'TextLabels',annotationPlate,'textFontSize',12);

colormap(cool);

title(['Optical density after 16hr - Day ' num2str(iDay)],'fontsize',24);

set(gcf,'position',[395 186 1132 762]);

savefig(['Optical density after 16hr - Day ' num2str(iDay) '.fig']);

saveas(gcf,['Optical density after 16hr - Day ' num2str(iDay) '.jpg']);

%% loadLookupTable.m

%% This script constructs a lookup table (simple cell array) that

%% relates the drug choice with its positions in liquid handler (labware and

%% rack position). The table also details the color code we use to mark the

%% drug and its concentration

% Drug(str) labware(str) source(str) rgb code (int int int)

lookupTable = { ...

'CIP [low]' '1' '1' hex2rgb('#c6f9fa'); ...

'CIP [med]' '1' '2' hex2rgb('#70d5fb'); ...

'CIP [high]' '1' '3' hex2rgb('#00adee'); ...

'KAN [low]' '1' '4' hex2rgb('#fff5d3'); ...

'KAN [med]' '1' '5' hex2rgb('#fce682'); ...

'KAN [high]' '1' '6' hex2rgb('#fadb02'); ...

'CHL [low]' '2' '1' hex2rgb('#fad5dc'); ...

'CHL [med]' '2' '2' hex2rgb('#f288a5'); ...

'CHL [high]' '2' '3' hex2rgb('#ec1c79'); ...

'ALL [low]' '2' '4' hex2rgb('#CCCCCC'); ...

'ALL [med]' '2' '5' hex2rgb('#AAAAAA'); ...

'ALL [high]' '2' '6' hex2rgb('#666666'); ...

'No drug' '2' '7' hex2rgb('#FFFFFF'); ...

'-' '2' '7' hex2rgb('#FFFFFF'); ...

};

%% google2csv.m

%% This script downloads instructions from a shared google spreadsheet

%% and converts it to CSV instructions readable by liquid handler (epMotion)

%% DEFINITIONS

% google sheet id and tabs (internal sheets) ids

DOCID = '1QsK89YX_sZy1JEnt7dMTj4gtkXpiez28y_hTlGUIDWg'; % ID taken from google doc link

gidArray = {'0' '1283593308' '1959478006' '704228922' '524138626' '1367285667' '1025729788' '7817454'}; % the gid allow to access a multi-tab google sheet

% A user defined lookup table (maps drug to labware/source/color code)

loadLookupTable;

%% download data from shared Google sheet

% download the raw data as cell array

fullDataset = {};

for i=1:length(gidArray)

curDataset = myGetGoogleSpreadsheet(DOCID,gidArray{i}); % a modified version of GetGoogleSpreadsheet

fullDataset{i} = curDataset;

end

%% request user input (which day of experiment to process)

prompt = {'Day of experiment to process'};

dlg_title = 'Extract data from Google doc ';

num_lines = 1;

defaultans = {'0'};

userInput = inputdlg(prompt,dlg_title,num_lines,defaultans);

iDay = str2num(userInput{1})

%% extract dataset for the specific day only

drugArray = cell(96,1); wellArray = cell(96,1); iPos = 1;

annotationPlate = cell(8,12);

for iRow = 1:8

for jCol = 1:12

curWell = fullDataset{iRow}{jCol+1,1};

curDrug = fullDataset{iRow}{jCol+1,iDay+1}; % positions need to be adjusted by +1 due to header entries in the table

wellArray{iPos,1} = curWell;

drugArray{iPos,1} = curDrug;

annotationPlate{iRow,jCol}=curDrug;

iPos = iPos+1;

end

end

%% save the anotationPlate variable

curFileStr = ['annotationPlate -' num2str(iDay)];

save(curFileStr,'annotationPlate');

%% convert to CSV file (use fprintf)

% definitions

vol = 200; % volume in uL

tool = 'TS_1000'; % pipette to use

colorfulPlate = nan(12,8);

% open a text file for writing

filename = ['epMotion_Day-' num2str(iDay, '%02.0f') '.csv'];

fileID = fopen(filename,'w');

% print text header (by epMotion convention)

fprintf(fileID,'Labware,Src.Barcode,Src.List Name,Dest.Barcode,Dest.List Name,,,\n');

fprintf(fileID,'1,,ResRack_1,,mtp96_1,,,\n');

fprintf(fileID,'2,,ResRack_2,,mtp96_1,,,\n');

fprintf(fileID,',,,,,,,\n');

fprintf(fileID,',,,,,,,\n');

fprintf(fileID,',,,,,,,\n');

fprintf(fileID,',,,,,,,\n');

fprintf(fileID,'Barcode ID,Labware,Source,Labware,Destination,Volume ,Tool,Name\n');

% print instructions per well

for iWell=1:length(wellArray)

[labware source,serial] = getLabwareSource(drugArray(iWell),lookupTable);

dest = wellArray{iWell};

str = [',' labware ',' source ',1,' dest ',' num2str(vol) ',' tool ','];

colorfulPlate(iWell) = serial;

fprintf(fileID,'%s\n',str);

end

fclose(fileID);

%% Prepare some sanity plots (representation of 96-well plate and organization of racks)

% plot the racks

h1 = figure('color','w');

subplot(2,1,1); hold on;

for i=1:6

bar(i,1,'facecolor',lookupTable{i,4});

end

bar(i+1,1,'facecolor','k');

set(gca,'xtick',[1:7],'xticklabel',{lookupTable{1:6,1} 'empty rack'},'xlim',[0.5 7.5],'ylim',[0 1],'ytick',[-1 1.1]);

box on;

title(['Rack #1 setup for day ' num2str(iDay)]);

subplot(2,1,2); hold on;

for i=7:13

bar(i,1,'facecolor',lookupTable{i,4});

end

set(gca,'xtick',[7:13],'xticklabel',{lookupTable{7:13,1}},'xlim',[6.5 13.5],'ylim',[0 1],'ytick',[-1 1.1]);

box on;

title(['Rack #2 setup for day ' num2str(iDay)]);

savefig(['Racks setup for day - ' num2str(iDay) '.fig']);

% plot the 96-well plate

figure; hold on;

myCmap = cat(1,lookupTable{[1:13],4});

microplateplot(colorfulPlate','TextLabels',annotationPlate,'textFontSize',8);

colormap(myCmap);

title(['Plate setup for day ' num2str(iDay)],'fontsize',24);

set(gcf,'position',[395 186 1132 762]);

savefig(['Plate setup for day - ' num2str(iDay) '.fig']);

saveas(gcf,['Plate setup for day - ' num2str(iDay) '.jpg']);

function [labware source, serial] = getLabwareSource(drugStr,lookupTable)

%function [labware source, serial] = getLabwareSource(drugStr,lookupTable)

% this function convert a string (describe a drug and concentration) to a

% positions (labware and source on the epMotion)

inx = find(strcmp(lookupTable(:,1),drugStr));

labware = lookupTable{inx,2};

source = lookupTable{inx,3};

serial = inx; % serial interger corresponding to drug 1-14
