# Supplementary material for: Harnessing robotic automation and web-based technologies to modernize scientific outreach
Source: PLoS Biol. 2019 Jun 26;17(6):e3000348. doi: 10.1371/journal.pbio.3000348 (PMC6615640; doi:10.1371/journal.pbio.3000348)
Supplement: S2 Code — (DOCX) [file pbio.3000348.s013.docx]

/*********************LED Display Code Set Up*******************************/

// Code is from TM1637Display Package, This is the chip code

#include <Arduino.h>

#include <TM1637Display.h>

// Module connection pins (Digital Pins)

#define CLK 2

#define DIO 3

// Time period (in milliseconds) between tests

#define TEST_DELAY  3000

TM1637Display display(CLK, DIO);

/********************* LED Display Code Set Up *******************************/

int sensorPin = A0;    // select the input data pin for the sensor

float rawRange = 1024;

float logRange = 5.0;

int smoothFactor = 100; // number of sensor measurements to average before reporting an OD  value

long blankOD = 0.0;

long rawOD = 0.0;

long curOD = 0.0;

long avgRawValue = 0.0;

void setup()

{

 delay(1000);

 analogReference(EXTERNAL);

**Serial**.begin(9600);

**Serial**.println("Adafruit Analog Light Sensor Test");

 pinMode(6, OUTPUT); //RED LED POWER

 pinMode(5, OUTPUT); //LED DISPLAY POWER

 //analogWrite(6,10);

 digitalWrite(4, HIGH); // RED LED

 digitalWrite(5, HIGH); // LED DISPLAY POWER

 display.setBrightness(0x0f);

}

void loop()

{

 int k;

 uint8_t data[] = { 0xff, 0xff, 0xff, 0xff };

 //Serial.print("   Lux = ");

 //Serial.println(RawToLux(rawValue));

 // for first measurement (blank)

 if(blankOD == 0.0){ // delay for 5 sec to stabilize current

    for(int i=5;i>0;i--) {

     display.showNumberDecEx(i,(0x80 >> 1),true);

     digitalWrite(4, HIGH); // RED LED

     delay(500);

     digitalWrite(4, LOW); // RED LED

     delay(500);

    }

   blankOD = getOD(); // get blank measurement

   for(int i=0;i<5;i++) { // blink display to indicate black measurement is done

     display.setBrightness(0);

     display.showNumberDecEx(0,(0x80 >> 1),true);

     delay(100);

     display.setBrightness(0x0f);

     display.showNumberDecEx(0,(0x80 >> 1),true);

     delay(50);

     }

 }

 else{ // standard OD measurements

   rawOD = getOD();

   curOD = rawOD-blankOD;

   if(curOD<0) { // correct negative OD to zero

     curOD = 0;

   }

   display.showNumberDecEx(curOD,(0x80 >> 1),true);

   //Serial.print(" Blank OD =");

   //Serial.print(blankOD);

   //Serial.print(" raw OD =");

**Serial**.println(rawOD);

   //Serial.print(" current OD =");

   //Serial.println(curOD);

 }

}

/*********************LED Display Lux Reading*******************************/

float getOD()

{

 digitalWrite(4, LOW); // RED LED

 delay(500);

 digitalWrite(4, HIGH); // RED LED

 delay(500);

 long curRawValue = 0.0;

 long sumRawValue = 0.0;

 for(int i=0;i<smoothFactor;i++) {

 curRawValue = analogRead(sensorPin);

 sumRawValue = sumRawValue+curRawValue;

 delay(10);

 //Serial.println(i);

 }

 avgRawValue = sumRawValue/smoothFactor;

 long curOD = RawToOD(avgRawValue);

 //Serial.print("Raw= ");

 //Serial.print(avgRawValue);

 return curOD;

}

/*********************Light Sensor Code*******************************/

float RawToLux(int raw)

{

 float logLux = raw * logRange / rawRange;

 return pow(10, logLux);

}

/*********************Light Sensor Code*******************************/

float RawToOD(float rawValue)

// Uses a linear function for coversion. This requires fitting the coefficents with an calibrated

// spectrophotometer (could be a commercial one). This needs to be done only once.

{

 float OD2 = ((-0.0083) * rawValue + 4.63);

 int OD1=OD2*100;

 return OD1;

}
