# Supplementary material for: Harnessing robotic automation and web-based technologies to modernize scientific outreach
Source: PLoS Biol. 2019 Jun 26;17(6):e3000348. doi: 10.1371/journal.pbio.3000348 (PMC6615640; doi:10.1371/journal.pbio.3000348)
Supplement: S1 Presentation — (PPTX) [file pbio.3000348.s016.pptx]

## Slide 1
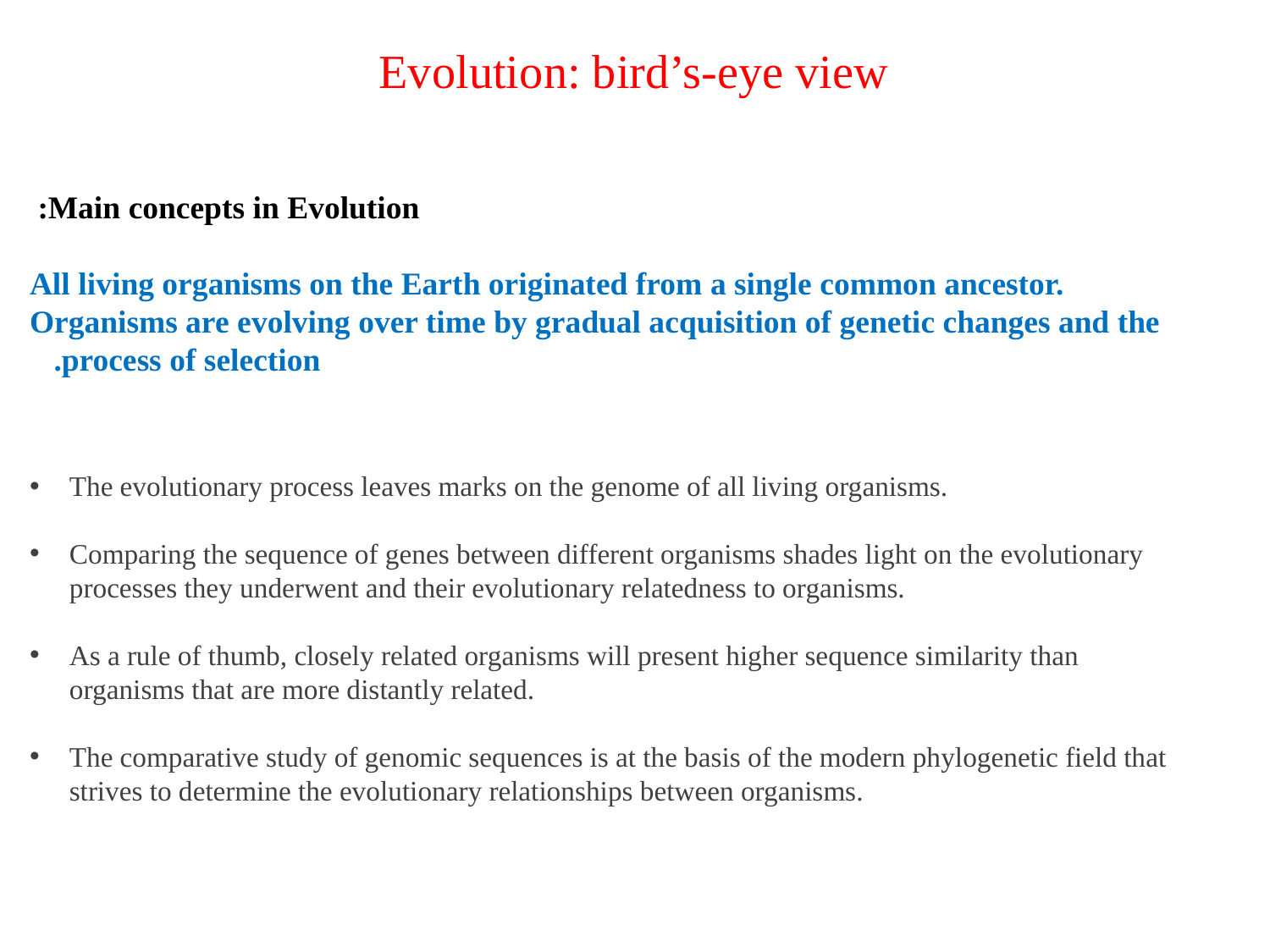

Evolution: bird’s-eye view
Main concepts in Evolution:
All living organisms on the Earth originated from a single common ancestor. Organisms are evolving over time by gradual acquisition of genetic changes and the process of selection.
The evolutionary process leaves marks on the genome of all living organisms.
Comparing the sequence of genes between different organisms shades light on the evolutionary processes they underwent and their evolutionary relatedness to organisms.
As a rule of thumb, closely related organisms will present higher sequence similarity than organisms that are more distantly related.
The comparative study of genomic sequences is at the basis of the modern phylogenetic field that strives to determine the evolutionary relationships between organisms.

## Slide 2
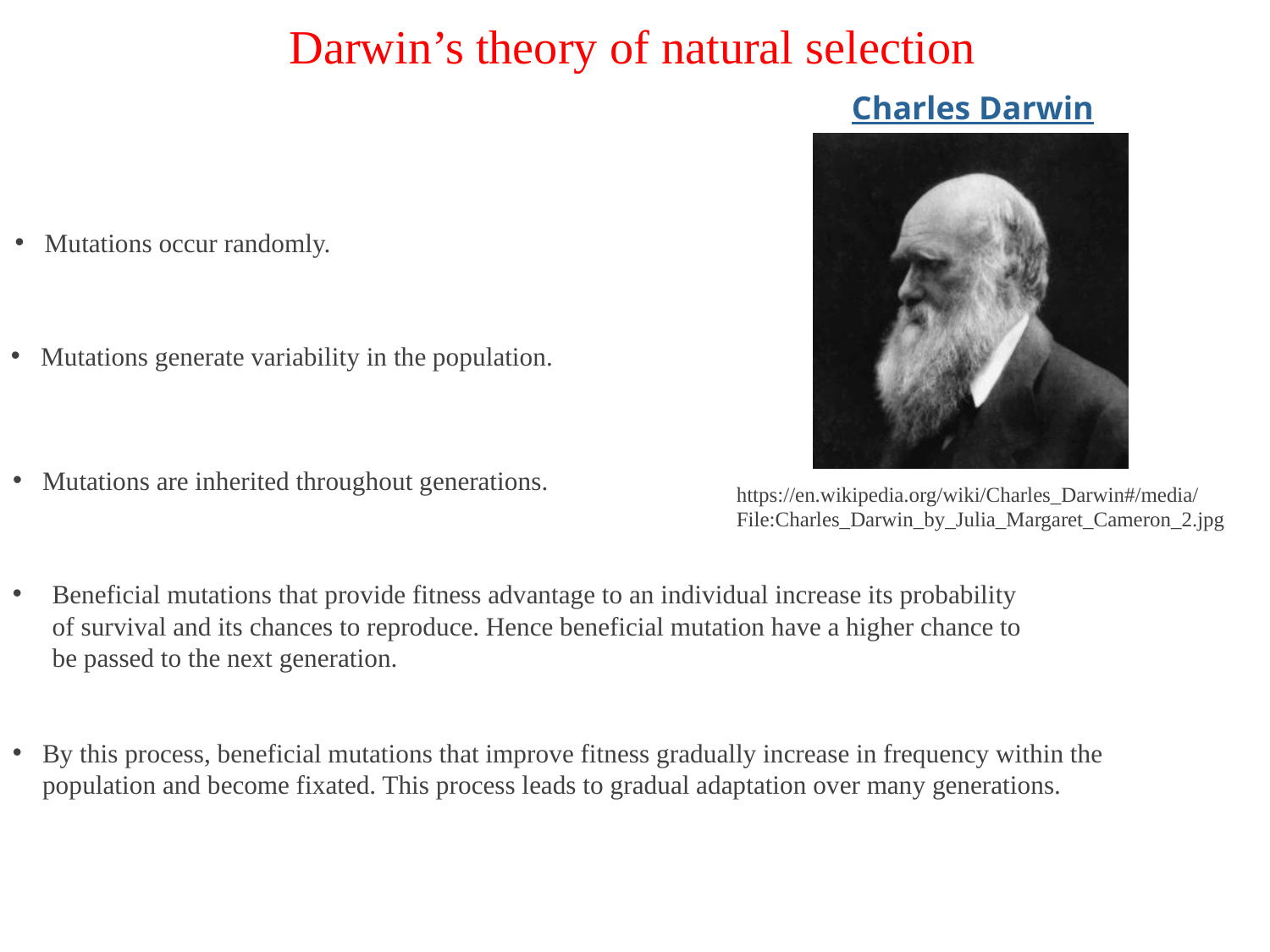

Darwin’s theory of natural selection
Charles Darwin
Mutations occur randomly.
Mutations generate variability in the population.
Mutations are inherited throughout generations.
https://en.wikipedia.org/wiki/Charles_Darwin#/media/
File:Charles_Darwin_by_Julia_Margaret_Cameron_2.jpg
Beneficial mutations that provide fitness advantage to an individual increase its probability of survival and its chances to reproduce. Hence beneficial mutation have a higher chance to be passed to the next generation.
By this process, beneficial mutations that improve fitness gradually increase in frequency within the population and become fixated. This process leads to gradual adaptation over many generations.

## Slide 3
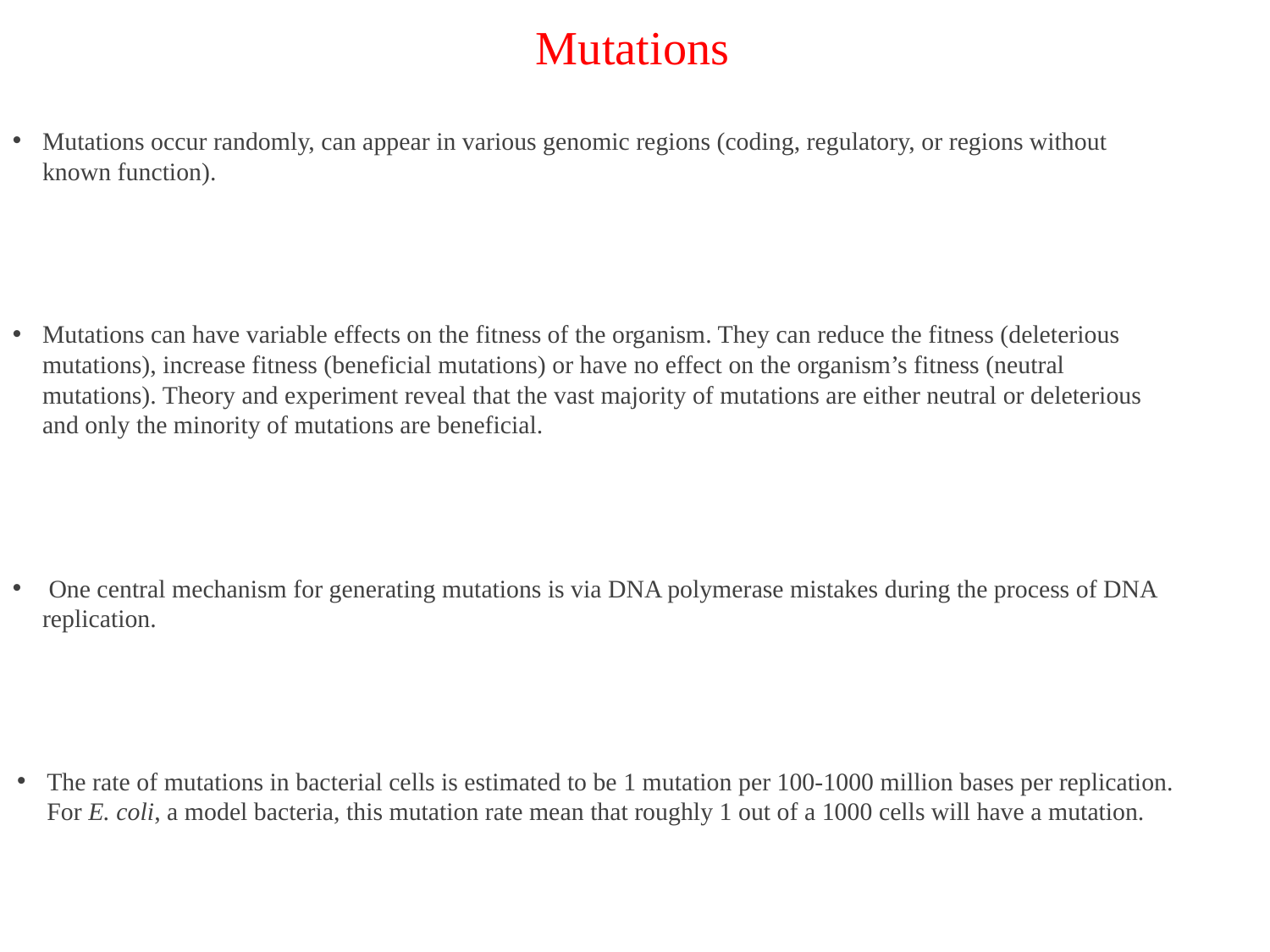

Mutations
Mutations occur randomly, can appear in various genomic regions (coding, regulatory, or regions without known function).
Mutations can have variable effects on the fitness of the organism. They can reduce the fitness (deleterious mutations), increase fitness (beneficial mutations) or have no effect on the organism’s fitness (neutral mutations). Theory and experiment reveal that the vast majority of mutations are either neutral or deleterious and only the minority of mutations are beneficial.
 One central mechanism for generating mutations is via DNA polymerase mistakes during the process of DNA replication.
The rate of mutations in bacterial cells is estimated to be 1 mutation per 100-1000 million bases per replication. For E. coli, a model bacteria, this mutation rate mean that roughly 1 out of a 1000 cells will have a mutation.

## Slide 4
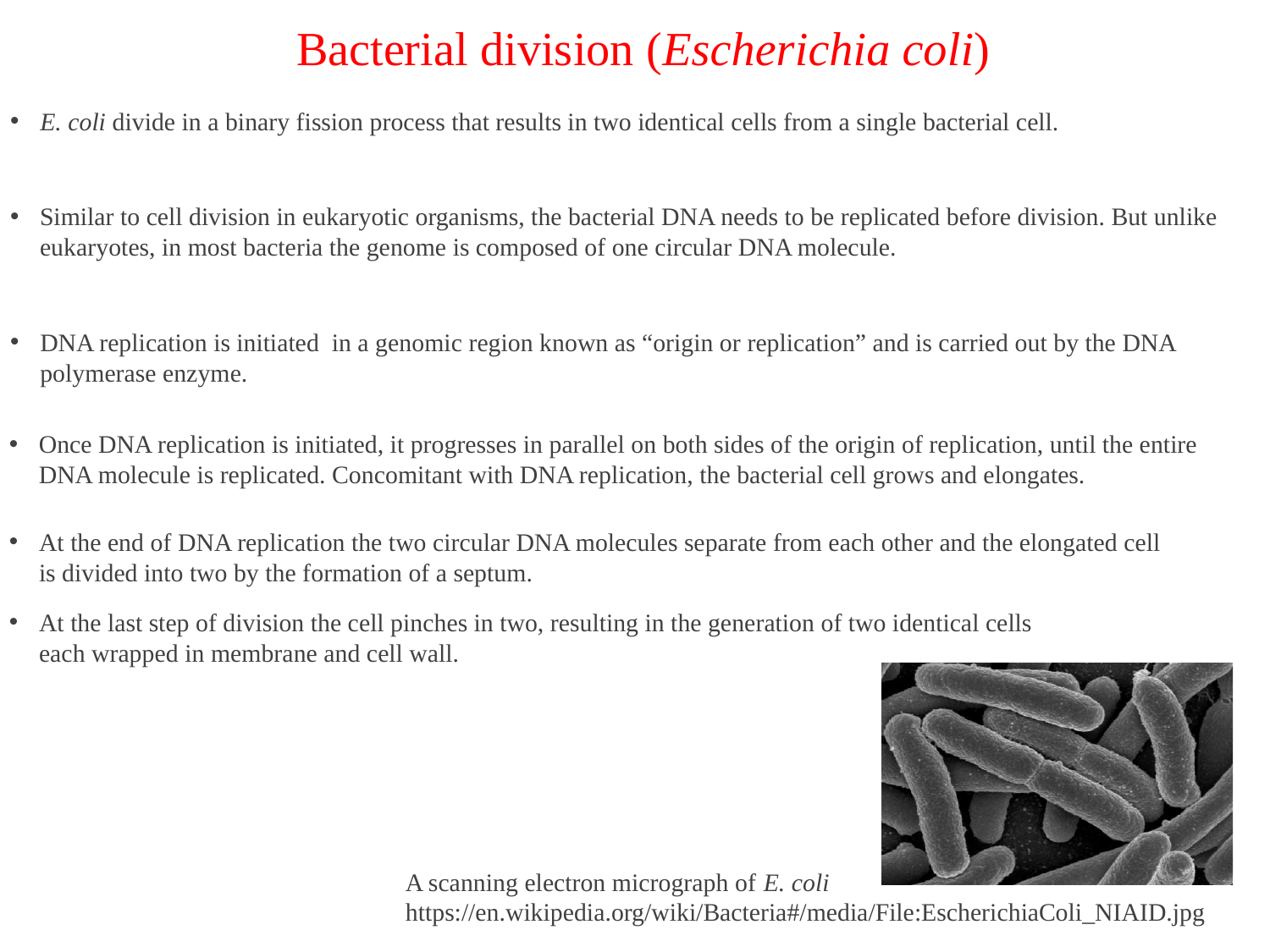

# Bacterial division (Escherichia coli)
E. coli divide in a binary fission process that results in two identical cells from a single bacterial cell.
Similar to cell division in eukaryotic organisms, the bacterial DNA needs to be replicated before division. But unlike eukaryotes, in most bacteria the genome is composed of one circular DNA molecule.
DNA replication is initiated in a genomic region known as “origin or replication” and is carried out by the DNA polymerase enzyme.
Once DNA replication is initiated, it progresses in parallel on both sides of the origin of replication, until the entire DNA molecule is replicated. Concomitant with DNA replication, the bacterial cell grows and elongates.
At the end of DNA replication the two circular DNA molecules separate from each other and the elongated cell is divided into two by the formation of a septum.
At the last step of division the cell pinches in two, resulting in the generation of two identical cells each wrapped in membrane and cell wall.
A scanning electron micrograph of E. coli
https://en.wikipedia.org/wiki/Bacteria#/media/File:EscherichiaColi_NIAID.jpg

## Slide 5
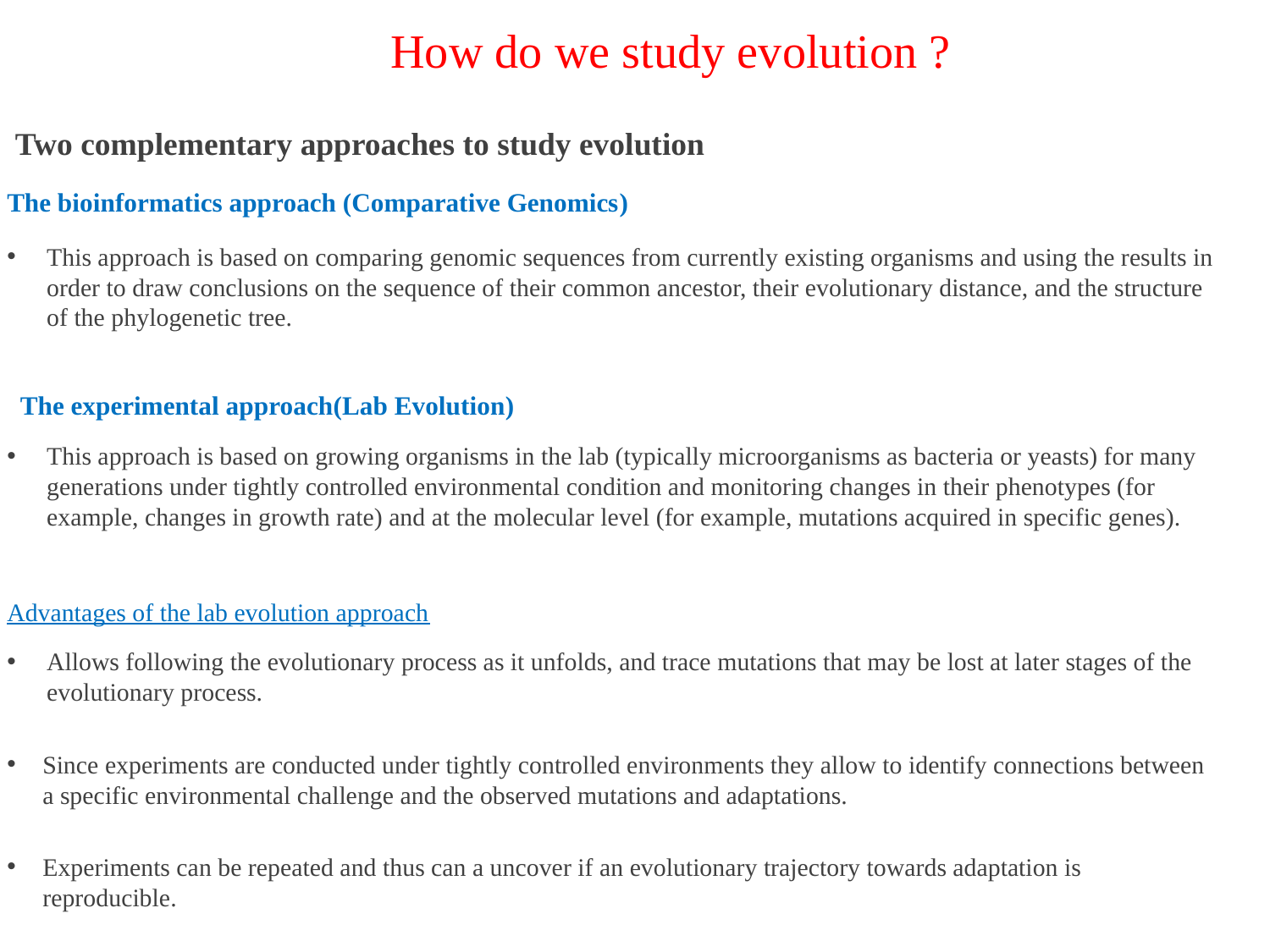

How do we study evolution ?
Two complementary approaches to study evolution
(The bioinformatics approach (Comparative Genomics
This approach is based on comparing genomic sequences from currently existing organisms and using the results in order to draw conclusions on the sequence of their common ancestor, their evolutionary distance, and the structure of the phylogenetic tree.
(Lab Evolution)The experimental approach
This approach is based on growing organisms in the lab (typically microorganisms as bacteria or yeasts) for many generations under tightly controlled environmental condition and monitoring changes in their phenotypes (for example, changes in growth rate) and at the molecular level (for example, mutations acquired in specific genes).
Advantages of the lab evolution approach
Allows following the evolutionary process as it unfolds, and trace mutations that may be lost at later stages of the evolutionary process.
Since experiments are conducted under tightly controlled environments they allow to identify connections between a specific environmental challenge and the observed mutations and adaptations.
Experiments can be repeated and thus can a uncover if an evolutionary trajectory towards adaptation is reproducible.

## Slide 6
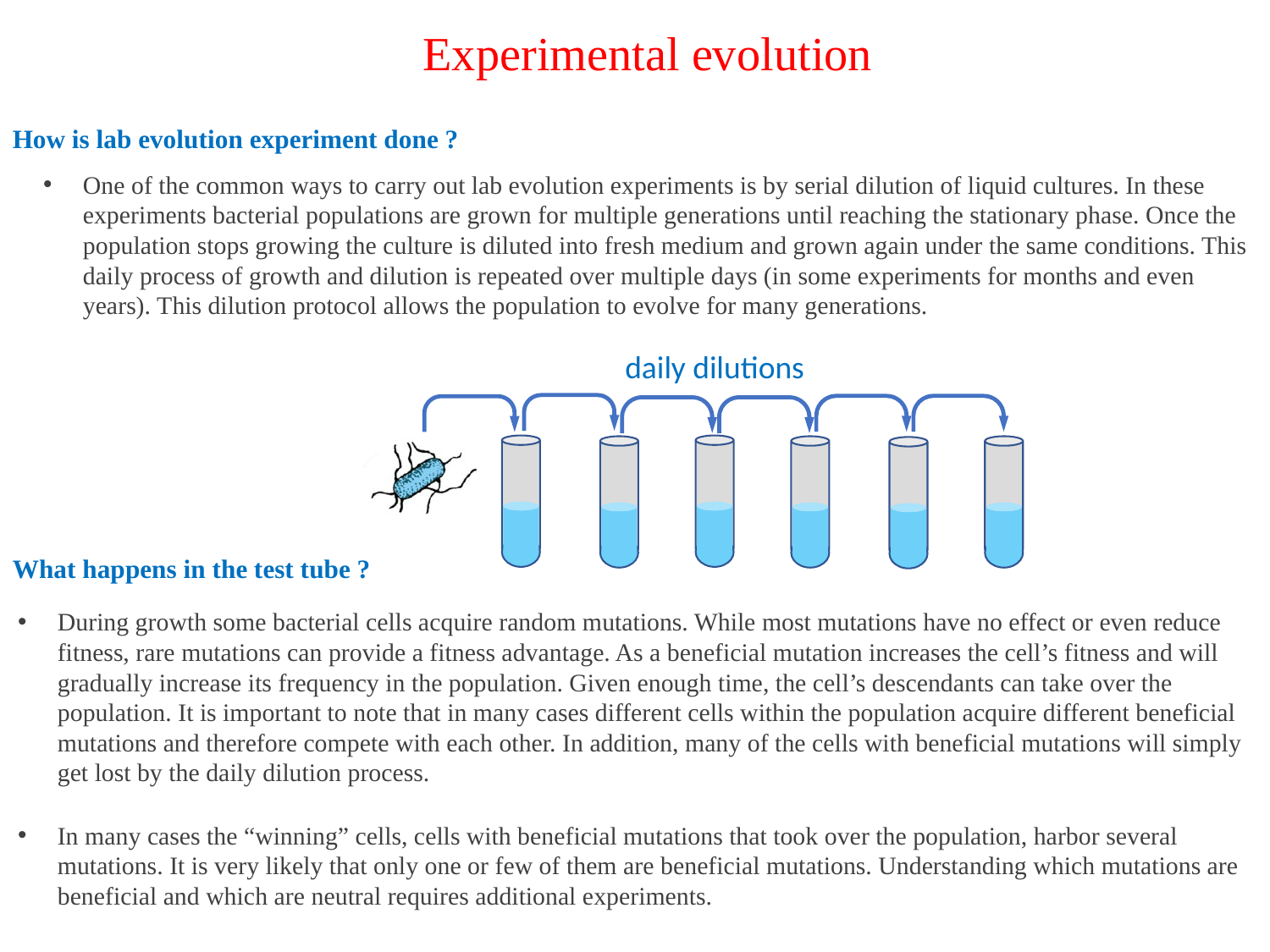

Experimental evolution
How is lab evolution experiment done ?
One of the common ways to carry out lab evolution experiments is by serial dilution of liquid cultures. In these experiments bacterial populations are grown for multiple generations until reaching the stationary phase. Once the population stops growing the culture is diluted into fresh medium and grown again under the same conditions. This daily process of growth and dilution is repeated over multiple days (in some experiments for months and even years). This dilution protocol allows the population to evolve for many generations.
daily dilutions
What happens in the test tube ?
During growth some bacterial cells acquire random mutations. While most mutations have no effect or even reduce fitness, rare mutations can provide a fitness advantage. As a beneficial mutation increases the cell’s fitness and will gradually increase its frequency in the population. Given enough time, the cell’s descendants can take over the population. It is important to note that in many cases different cells within the population acquire different beneficial mutations and therefore compete with each other. In addition, many of the cells with beneficial mutations will simply get lost by the daily dilution process.
In many cases the “winning” cells, cells with beneficial mutations that took over the population, harbor several mutations. It is very likely that only one or few of them are beneficial mutations. Understanding which mutations are beneficial and which are neutral requires additional experiments.

## Slide 7
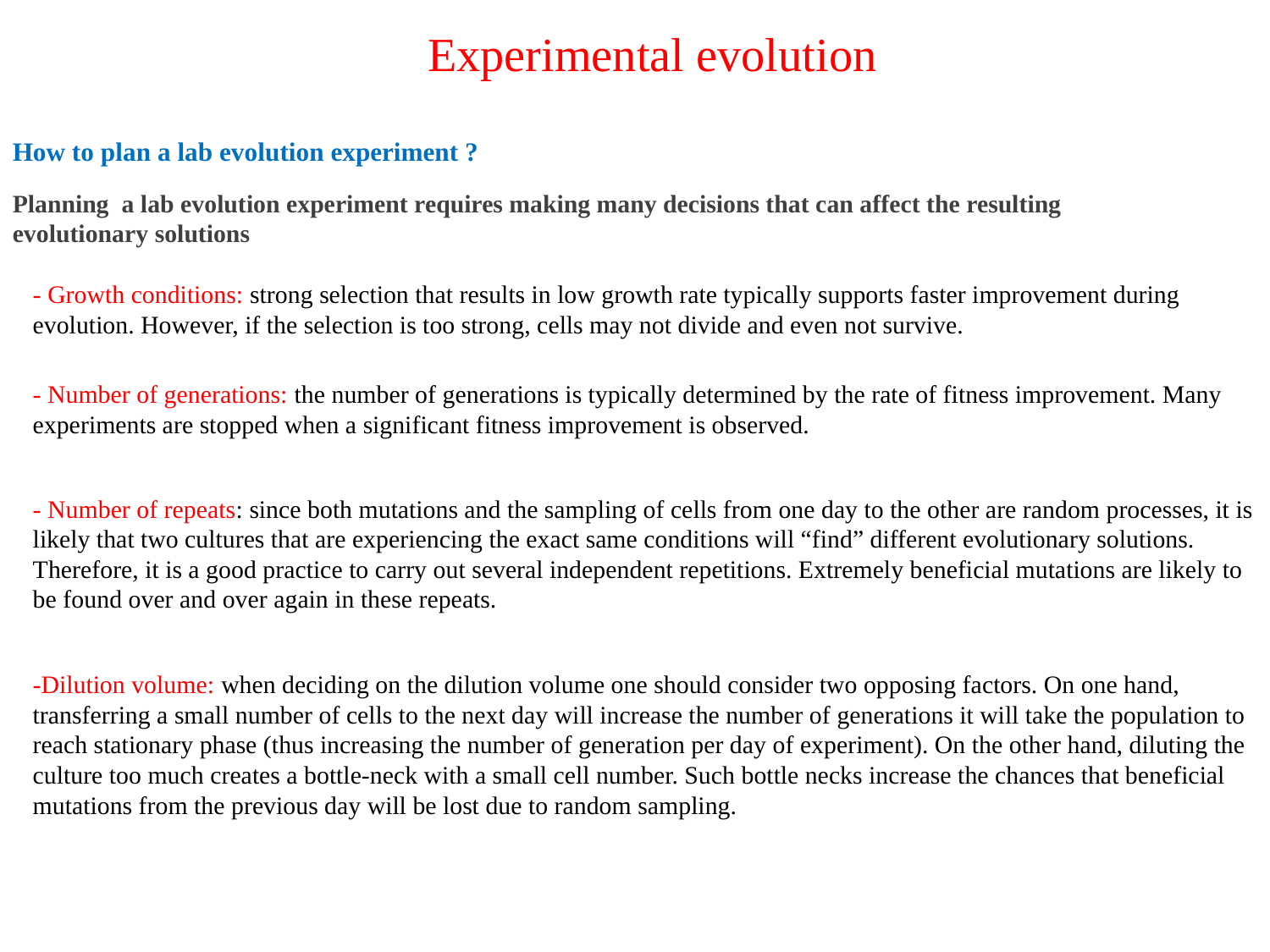

Experimental evolution
How to plan a lab evolution experiment ?
Planning a lab evolution experiment requires making many decisions that can affect the resulting evolutionary solutions
- Growth conditions: strong selection that results in low growth rate typically supports faster improvement during evolution. However, if the selection is too strong, cells may not divide and even not survive.
- Number of generations: the number of generations is typically determined by the rate of fitness improvement. Many experiments are stopped when a significant fitness improvement is observed.
- Number of repeats: since both mutations and the sampling of cells from one day to the other are random processes, it is likely that two cultures that are experiencing the exact same conditions will “find” different evolutionary solutions. Therefore, it is a good practice to carry out several independent repetitions. Extremely beneficial mutations are likely to be found over and over again in these repeats.
-Dilution volume: when deciding on the dilution volume one should consider two opposing factors. On one hand, transferring a small number of cells to the next day will increase the number of generations it will take the population to reach stationary phase (thus increasing the number of generation per day of experiment). On the other hand, diluting the culture too much creates a bottle-neck with a small cell number. Such bottle necks increase the chances that beneficial mutations from the previous day will be lost due to random sampling.

## Slide 8
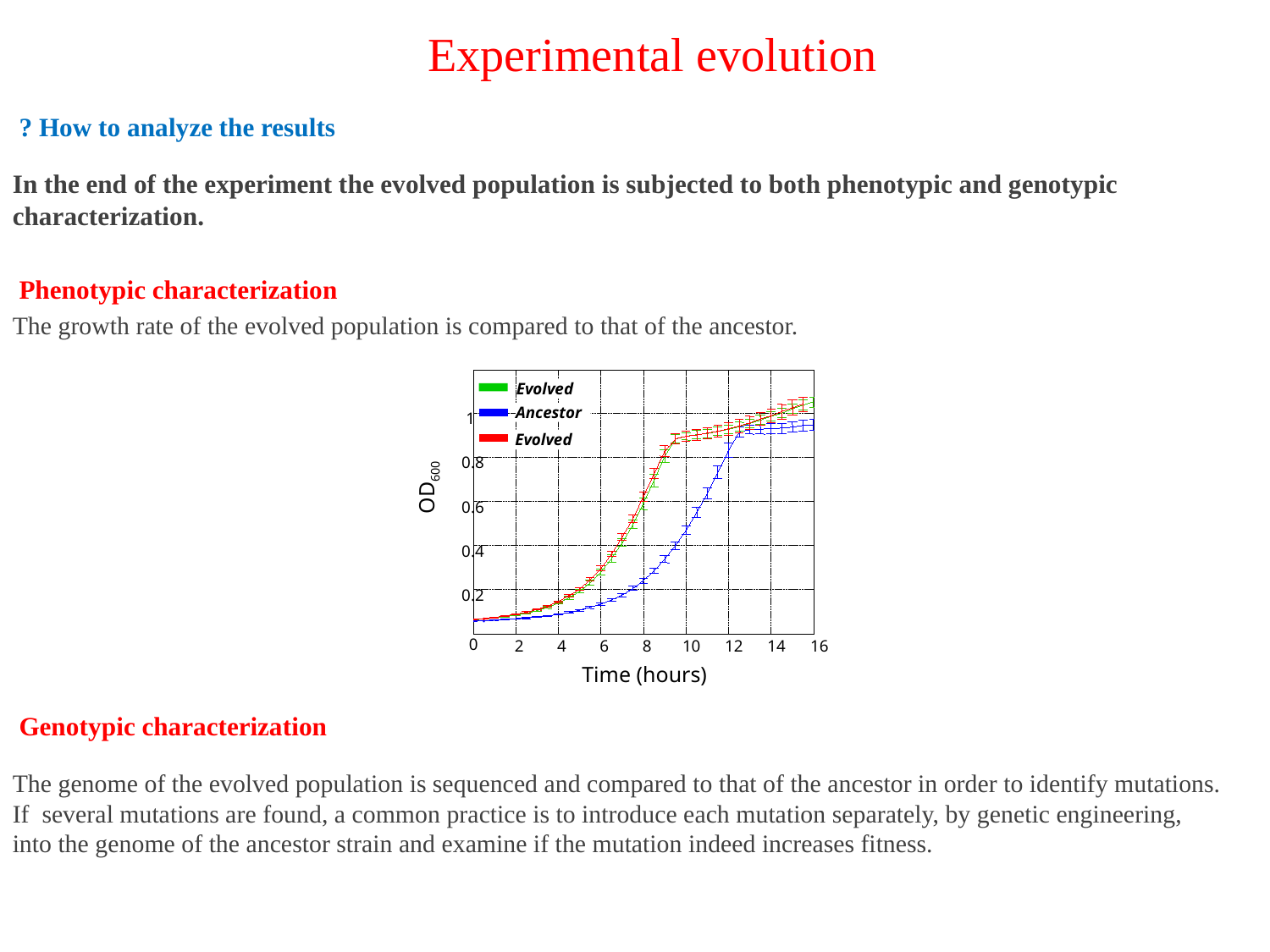

Experimental evolution
How to analyze the results ?
In the end of the experiment the evolved population is subjected to both phenotypic and genotypic characterization.
Phenotypic characterization
The growth rate of the evolved population is compared to that of the ancestor.
1
0.8
0.6
0.4
0.2
0
2
4
6
8
10
12
14
16
Evolved
Ancestor
Evolved
OD600
Time (hours)
Genotypic characterization
The genome of the evolved population is sequenced and compared to that of the ancestor in order to identify mutations. If several mutations are found, a common practice is to introduce each mutation separately, by genetic engineering, into the genome of the ancestor strain and examine if the mutation indeed increases fitness.

## Slide 9
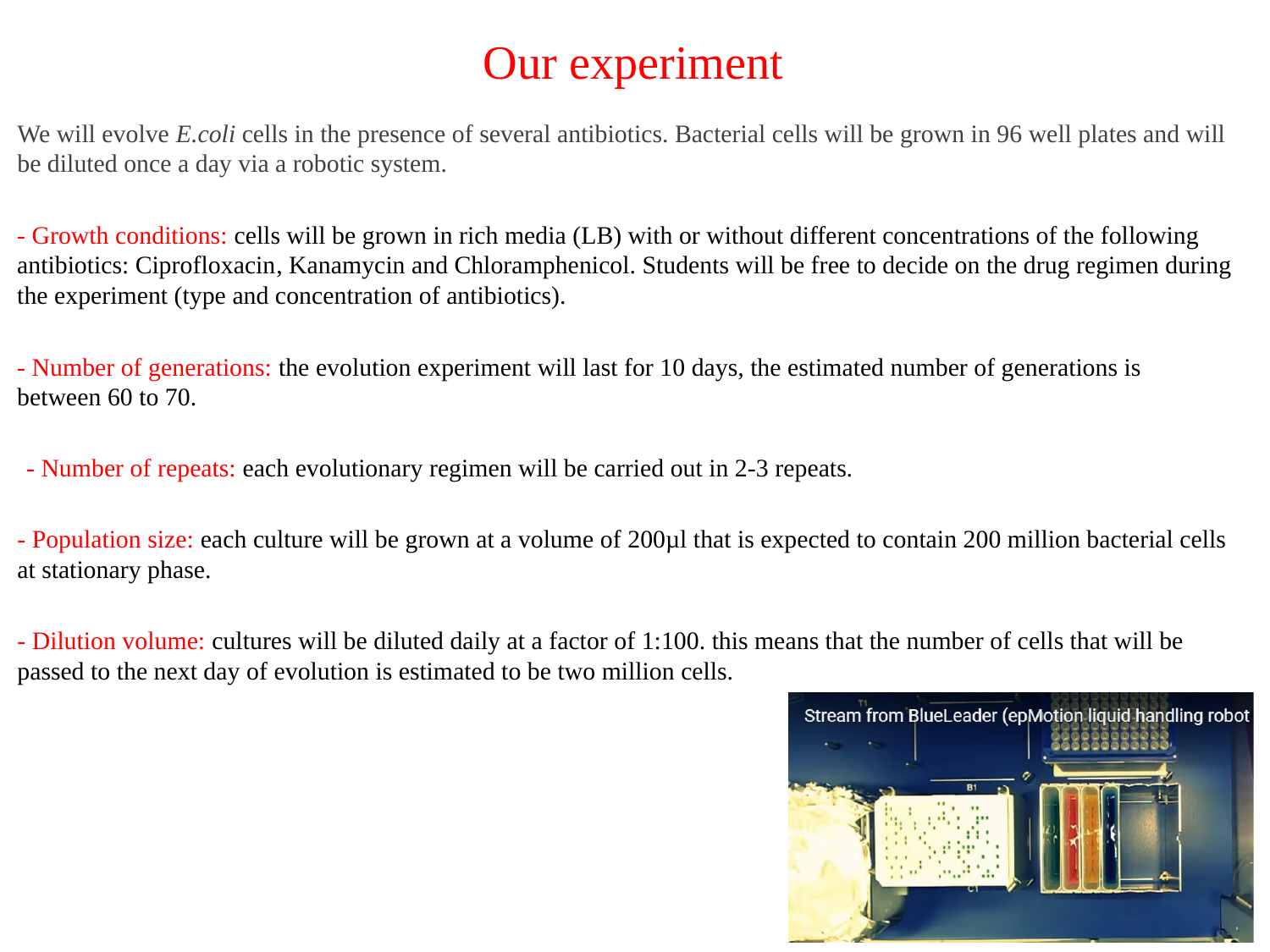

Our experiment
We will evolve E.coli cells in the presence of several antibiotics. Bacterial cells will be grown in 96 well plates and will be diluted once a day via a robotic system.
- Growth conditions: cells will be grown in rich media (LB) with or without different concentrations of the following antibiotics: Ciprofloxacin, Kanamycin and Chloramphenicol. Students will be free to decide on the drug regimen during the experiment (type and concentration of antibiotics).
- Number of generations: the evolution experiment will last for 10 days, the estimated number of generations is between 60 to 70.
 - Number of repeats: each evolutionary regimen will be carried out in 2-3 repeats.
- Population size: each culture will be grown at a volume of 200µl that is expected to contain 200 million bacterial cells at stationary phase.
- Dilution volume: cultures will be diluted daily at a factor of 1:100. this means that the number of cells that will be passed to the next day of evolution is estimated to be two million cells.

## Slide 10
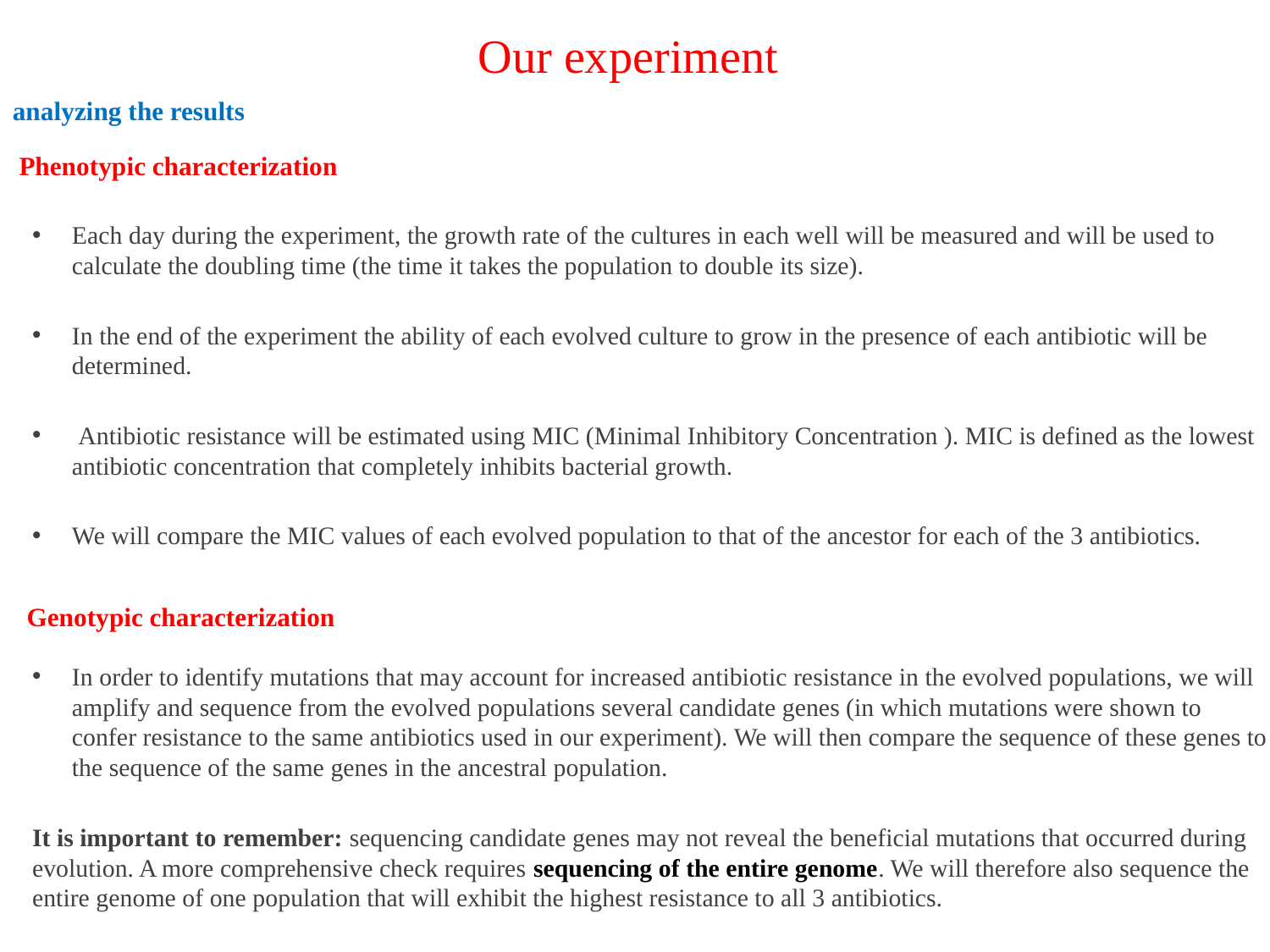

Our experiment
analyzing the results
Phenotypic characterization
Each day during the experiment, the growth rate of the cultures in each well will be measured and will be used to calculate the doubling time (the time it takes the population to double its size).
In the end of the experiment the ability of each evolved culture to grow in the presence of each antibiotic will be determined.
 Antibiotic resistance will be estimated using MIC (Minimal Inhibitory Concentration ). MIC is defined as the lowest antibiotic concentration that completely inhibits bacterial growth.
We will compare the MIC values of each evolved population to that of the ancestor for each of the 3 antibiotics.
Genotypic characterization
In order to identify mutations that may account for increased antibiotic resistance in the evolved populations, we will amplify and sequence from the evolved populations several candidate genes (in which mutations were shown to confer resistance to the same antibiotics used in our experiment). We will then compare the sequence of these genes to the sequence of the same genes in the ancestral population.
It is important to remember: sequencing candidate genes may not reveal the beneficial mutations that occurred during evolution. A more comprehensive check requires sequencing of the entire genome. We will therefore also sequence the entire genome of one population that will exhibit the highest resistance to all 3 antibiotics.
