# Supplementary material for: Harnessing robotic automation and web-based technologies to modernize scientific outreach
Source: PLoS Biol. 2019 Jun 26;17(6):e3000348. doi: 10.1371/journal.pbio.3000348 (PMC6615640; doi:10.1371/journal.pbio.3000348)
Supplement: S2 Presentation — (PPTX) [file pbio.3000348.s017.pptx]

## Slide 1
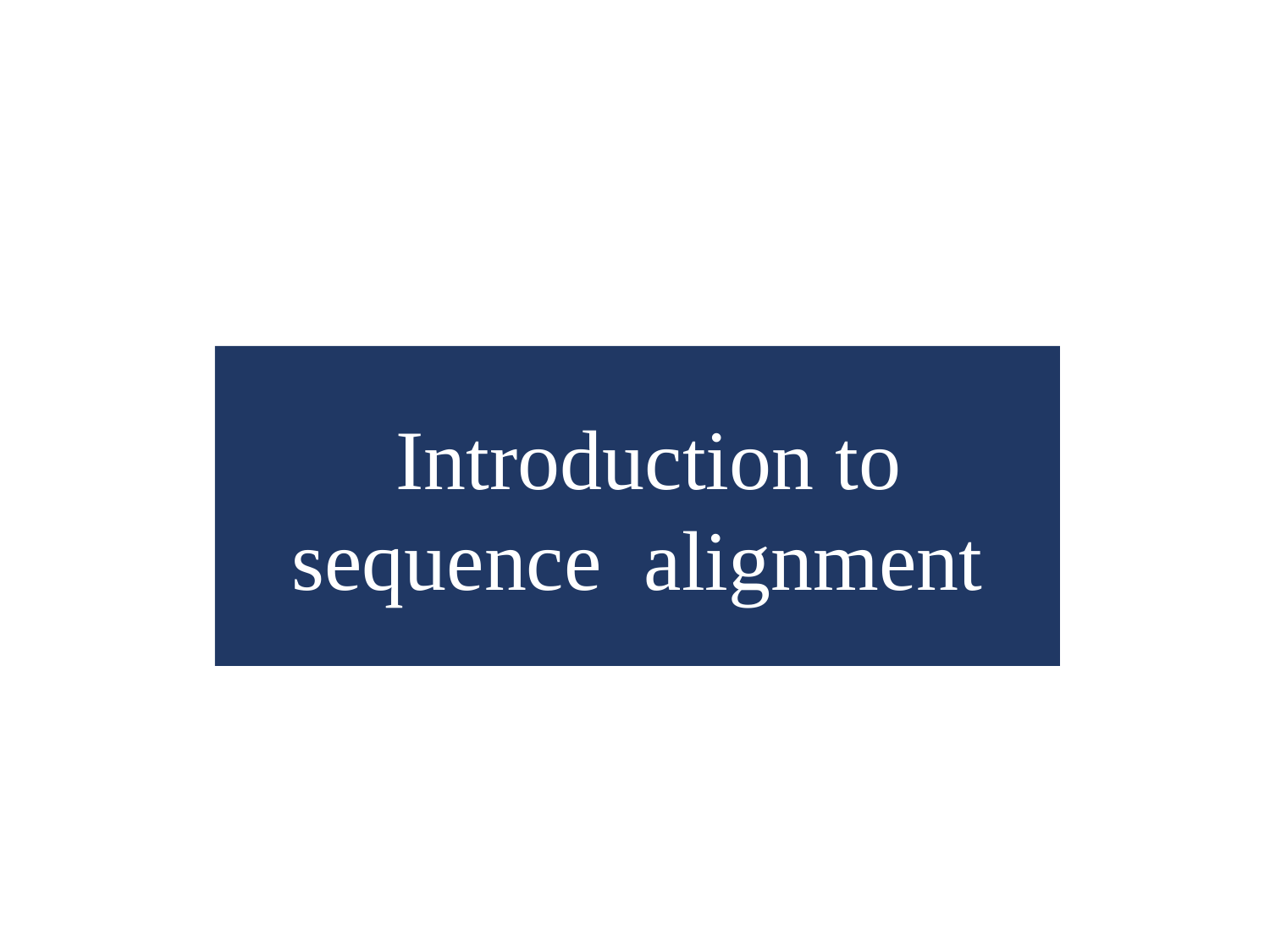

Introduction to
sequence alignment

## Slide 2
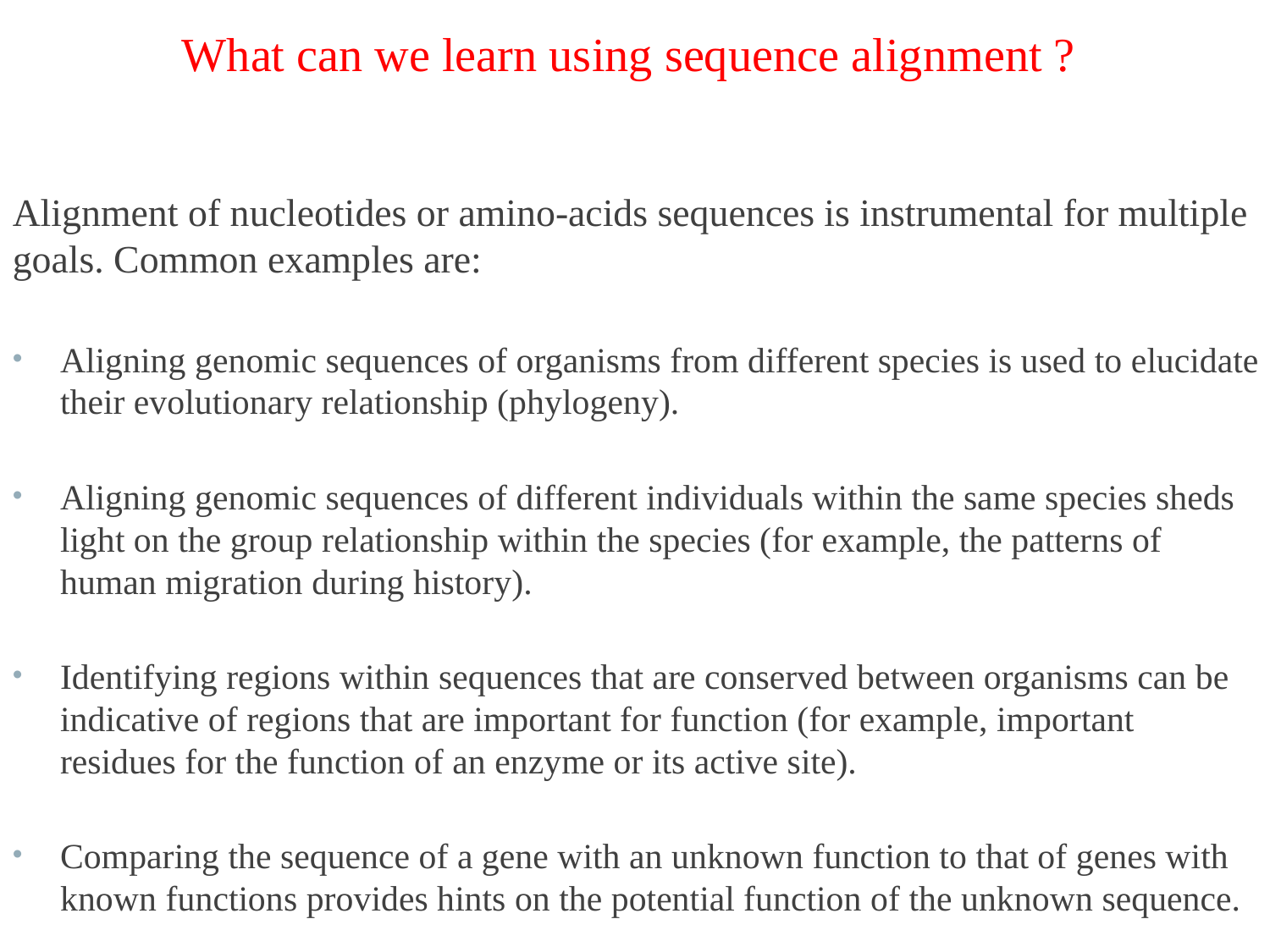

What can we learn using sequence alignment ?
Alignment of nucleotides or amino-acids sequences is instrumental for multiple goals. Common examples are:
Aligning genomic sequences of organisms from different species is used to elucidate their evolutionary relationship (phylogeny).
Aligning genomic sequences of different individuals within the same species sheds light on the group relationship within the species (for example, the patterns of human migration during history).
Identifying regions within sequences that are conserved between organisms can be indicative of regions that are important for function (for example, important residues for the function of an enzyme or its active site).
Comparing the sequence of a gene with an unknown function to that of genes with known functions provides hints on the potential function of the unknown sequence.
.

## Slide 3
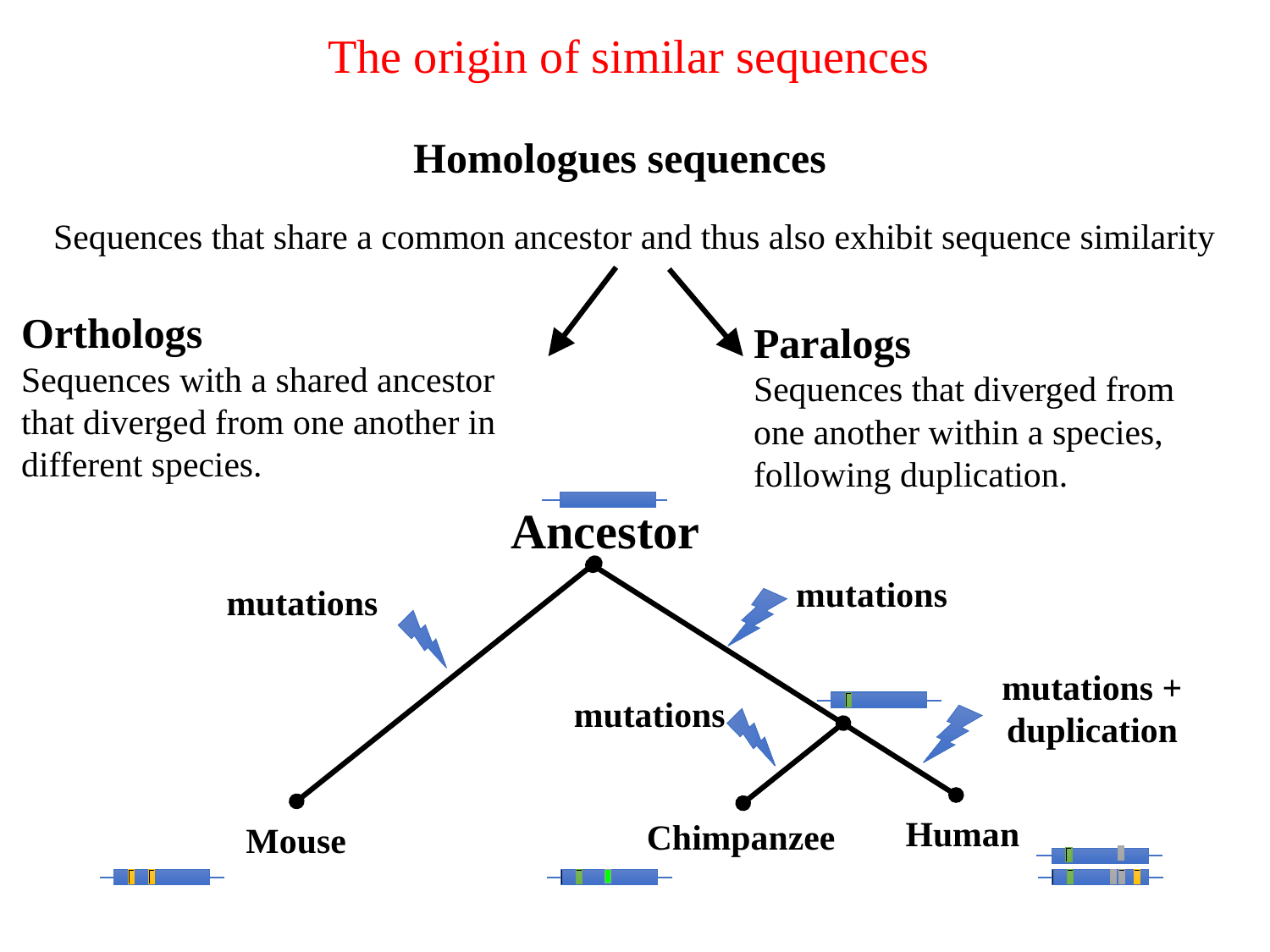

The origin of similar sequences
Homologues sequences
Sequences that share a common ancestor and thus also exhibit sequence similarity
Orthologs
Sequences with a shared ancestor that diverged from one another in different species.
Paralogs
Sequences that diverged from one another within a species, following duplication.
Ancestor
mutations
mutations
mutations +
duplication
mutations
Human
Chimpanzee
Mouse

## Slide 4
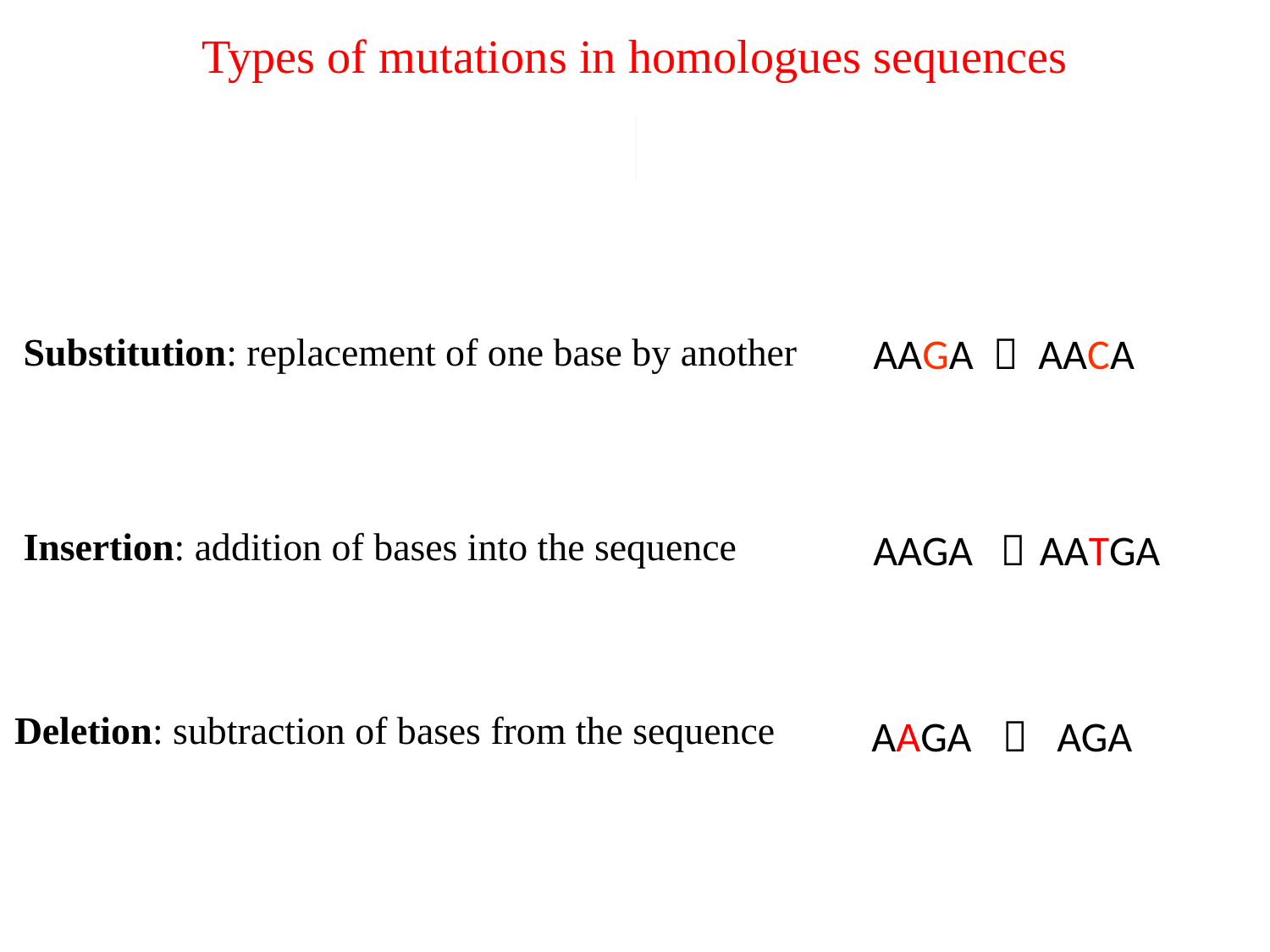

Types of mutations in homologues sequences
AAGA

AACA
 Substitution: replacement of one base by another

AAGA
AATGA
 Insertion: addition of bases into the sequence
 Deletion: subtraction of bases from the sequence
AAGA

AGA

## Slide 5
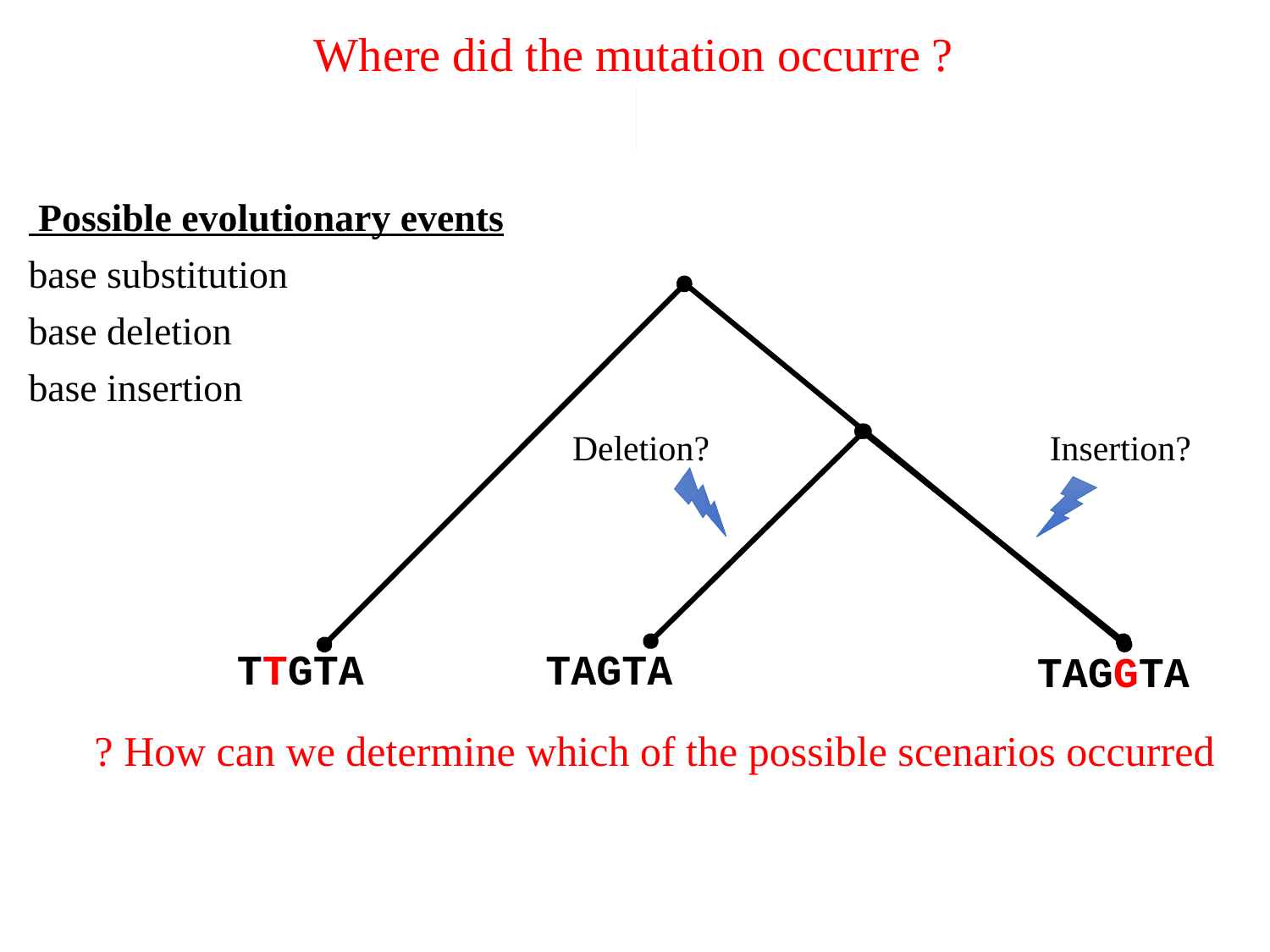

Where did the mutation occurre ?
Possible evolutionary events
base substitution
base deletion
base insertion
Deletion?
Insertion?
TAGGTA
TTGTA
TAGTA
How can we determine which of the possible scenarios occurred ?

## Slide 6
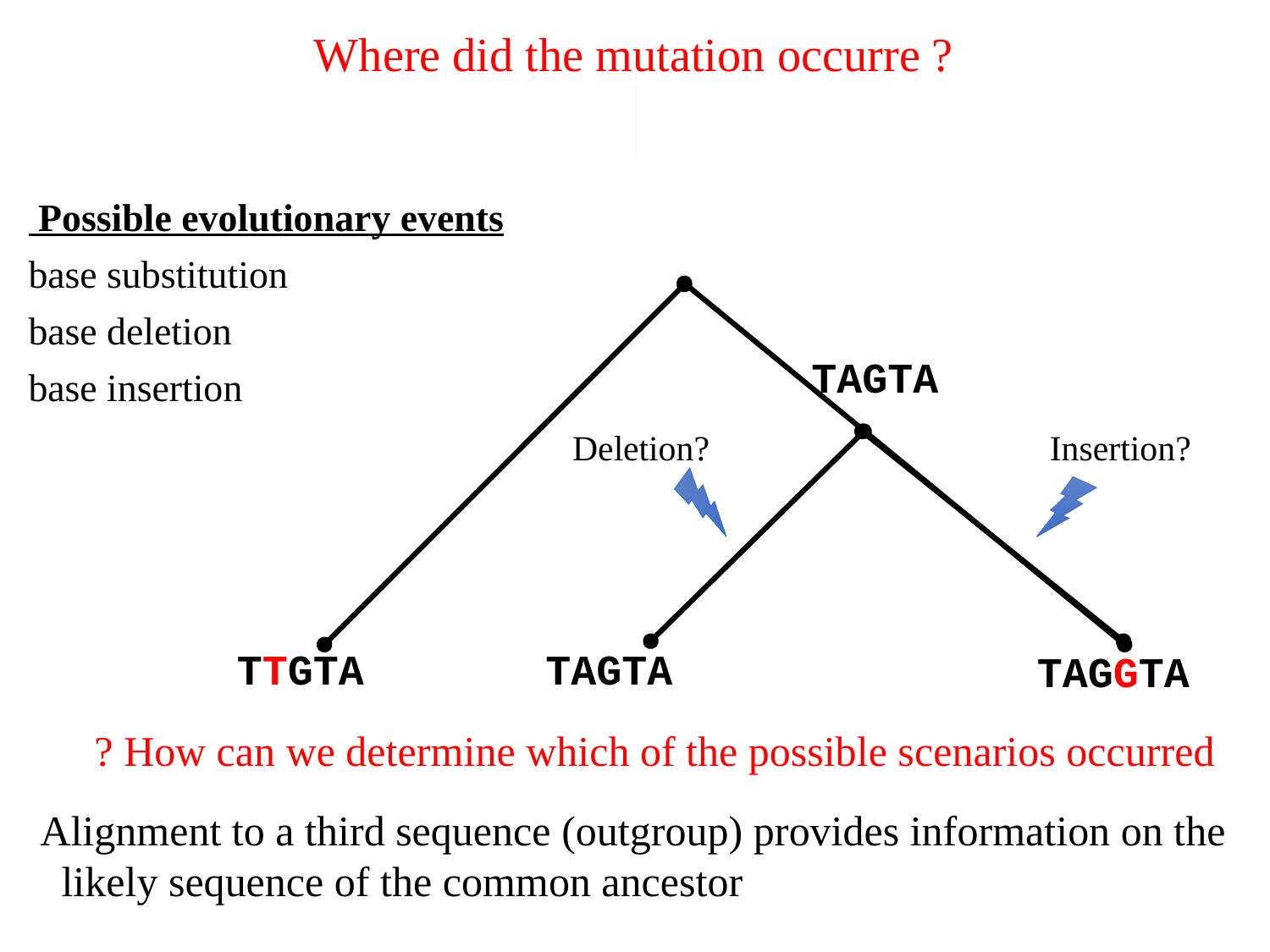

Where did the mutation occurre ?
Possible evolutionary events
base substitution
base deletion
base insertion
TAGTA
Deletion?
Insertion?
TAGGTA
TTGTA
TAGTA
How can we determine which of the possible scenarios occurred ?
Alignment to a third sequence (outgroup) provides information on the likely sequence of the common ancestor

## Slide 7
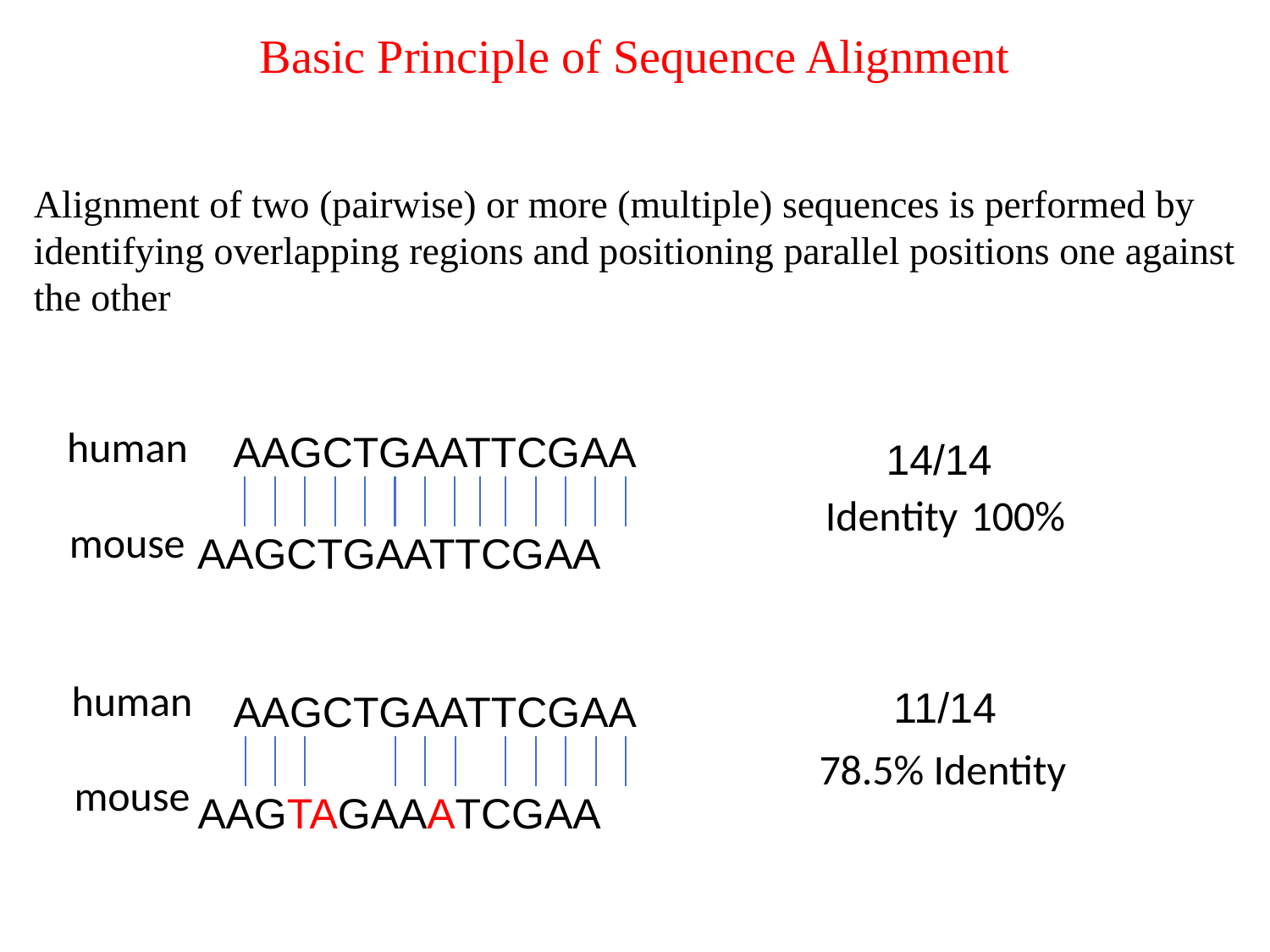

Basic Principle of Sequence Alignment
Alignment of two (pairwise) or more (multiple) sequences is performed by identifying overlapping regions and positioning parallel positions one against the other
human
AAGCTGAATTCGAA
AAGCTGAATTCGAA
14/14
Identity-100%
mouse
human
11/14
AAGCTGAATTCGAA
AAGTAGAAATCGAA
78.5% Identity-
mouse

## Slide 8
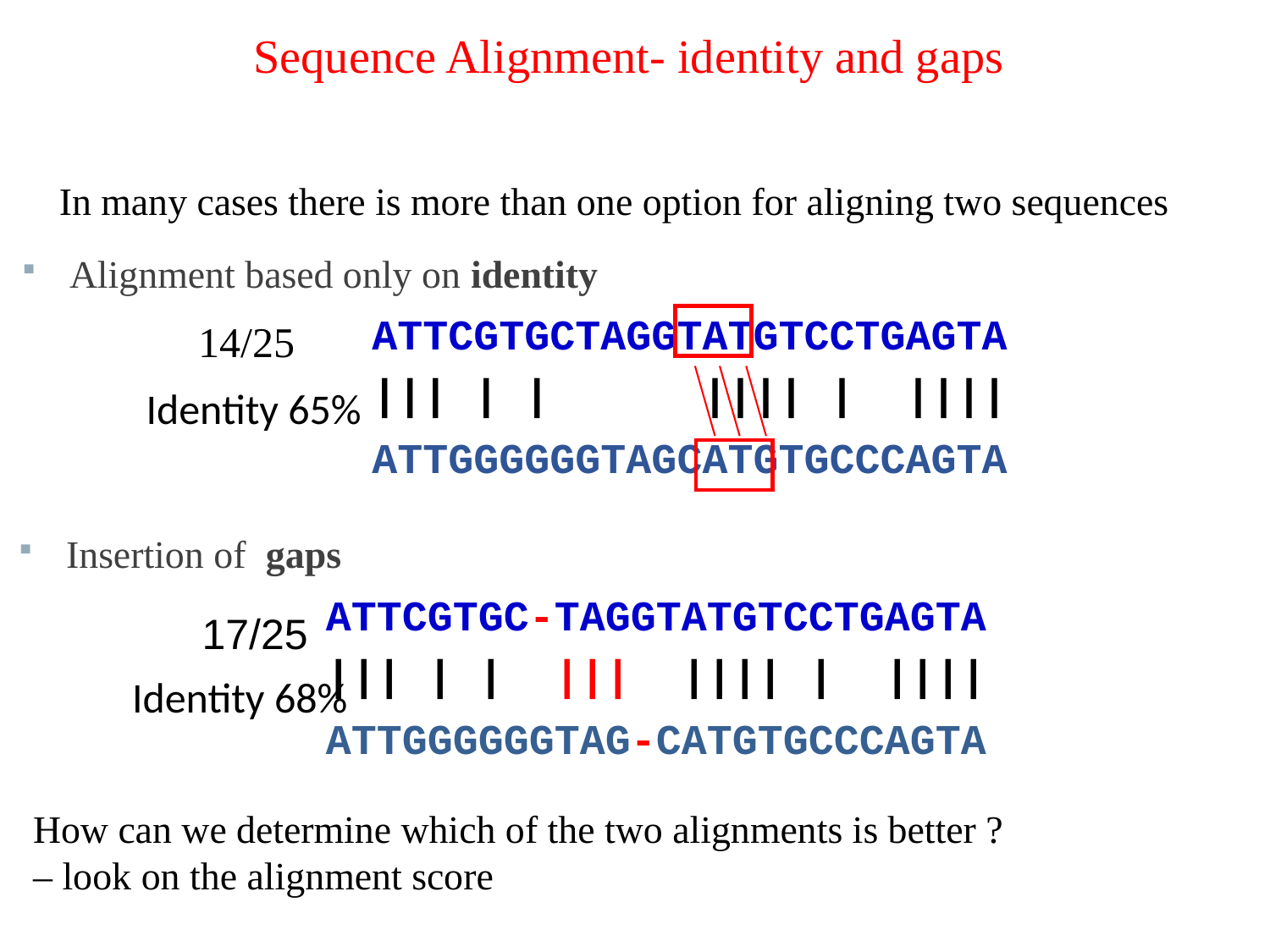

Sequence Alignment- identity and gaps
In many cases there is more than one option for aligning two sequences
Alignment based only on identity
ATTCGTGCTAGGTATGTCCTGAGTA
||| | | |||| | ||||
ATTGGGGGGTAGCATGTGCCCAGTA
14/25
Identity 65%-
Insertion of gaps
ATTCGTGC-TAGGTATGTCCTGAGTA
||| | | ||| |||| | ||||
ATTGGGGGGTAG-CATGTGCCCAGTA
17/25
Identity 68%-
How can we determine which of the two alignments is better ? – look on the alignment score

## Slide 9
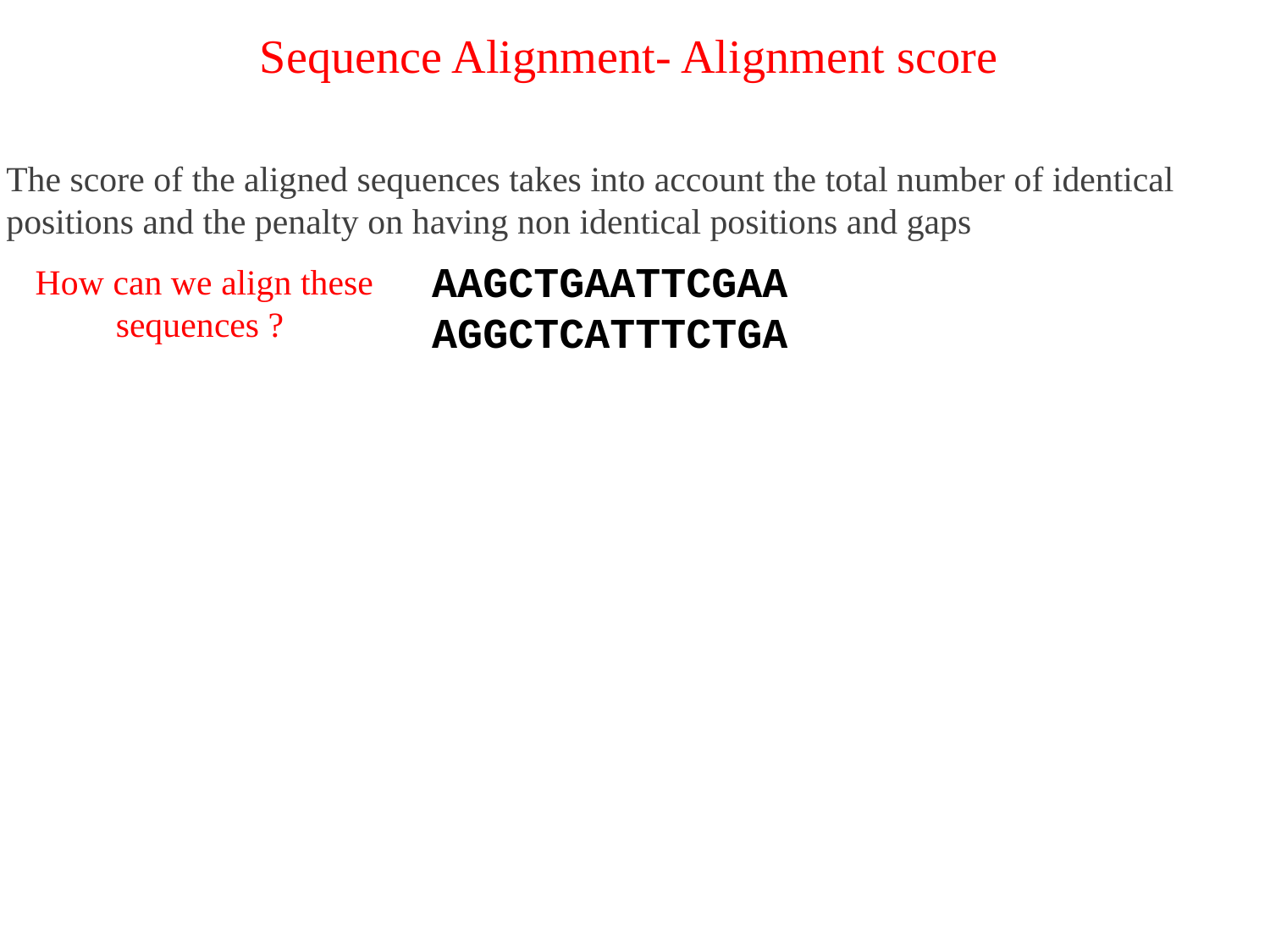

Sequence Alignment- Alignment score
The score of the aligned sequences takes into account the total number of identical positions and the penalty on having non identical positions and gaps
AAGCTGAATTCGAA
AGGCTCATTTCTGA
How can we align these sequences ?

## Slide 10
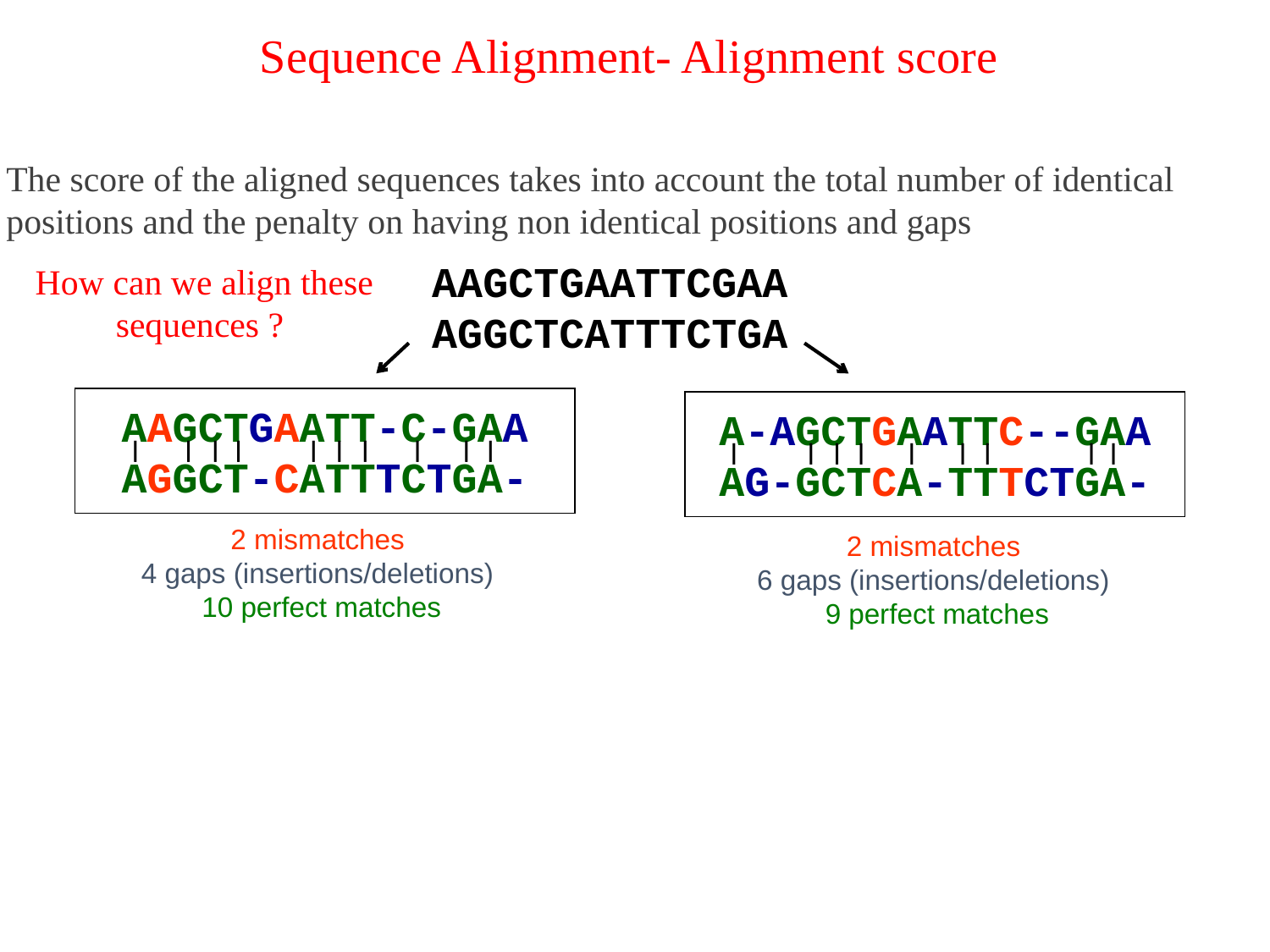

Sequence Alignment- Alignment score
The score of the aligned sequences takes into account the total number of identical positions and the penalty on having non identical positions and gaps
AAGCTGAATTCGAA
AGGCTCATTTCTGA
How can we align these sequences ?
AAGCTGAATT-C-GAA
AGGCT-CATTTCTGA-
ן
ן
ן
ן
ן
ן
ן
ן
ן
ן
A-AGCTGAATTC--GAA
AG-GCTCA-TTTCTGA-
ן
ן
ן
ן
ן
ן
ן
ן
ן
2 mismatches
4 gaps (insertions/deletions)
10 perfect matches
2 mismatches
6 gaps (insertions/deletions)
9 perfect matches

## Slide 11
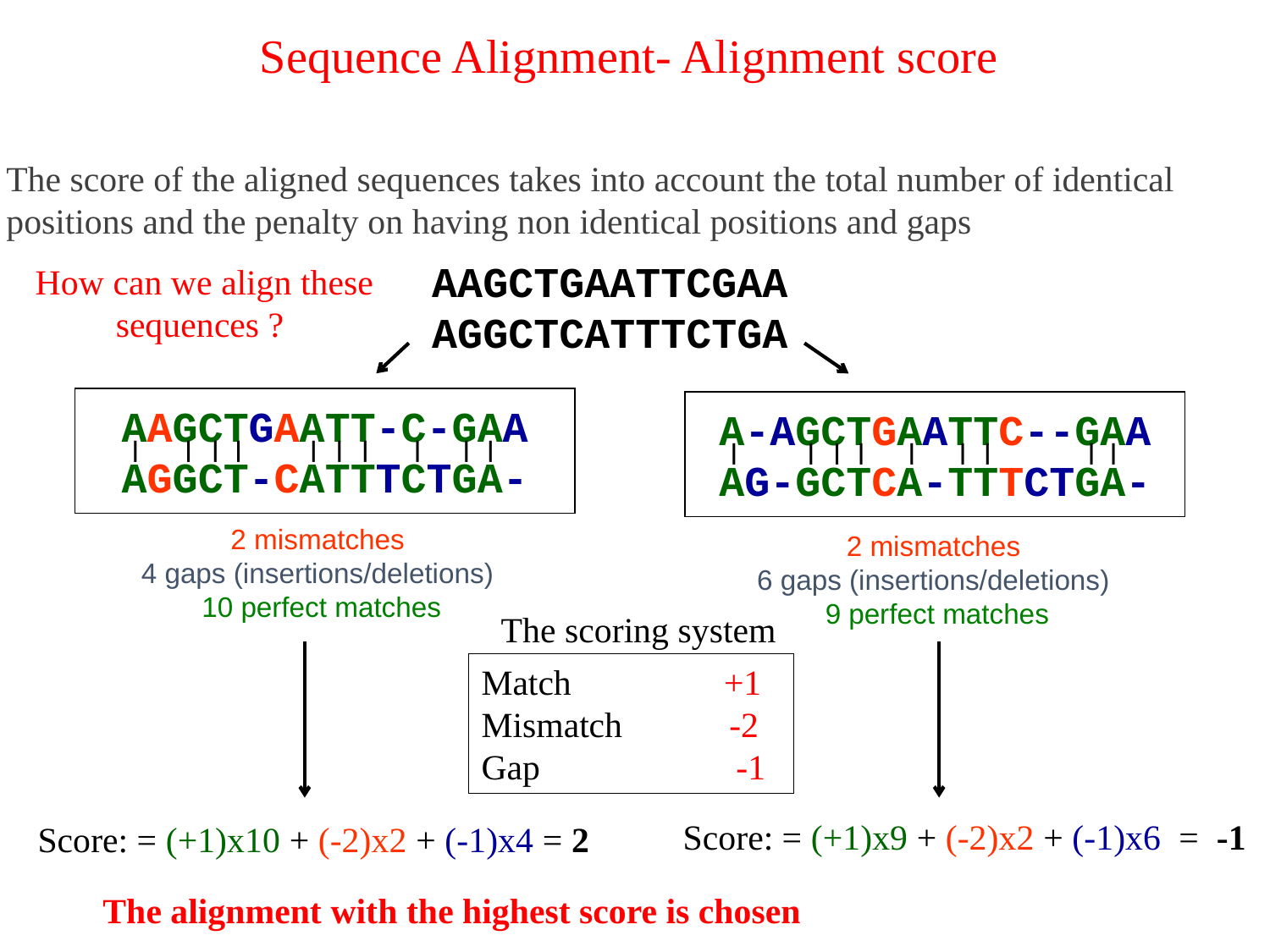

Sequence Alignment- Alignment score
The score of the aligned sequences takes into account the total number of identical positions and the penalty on having non identical positions and gaps
AAGCTGAATTCGAA
AGGCTCATTTCTGA
How can we align these sequences ?
AAGCTGAATT-C-GAA
AGGCT-CATTTCTGA-
ן
ן
ן
ן
ן
ן
ן
ן
ן
ן
A-AGCTGAATTC--GAA
AG-GCTCA-TTTCTGA-
ן
ן
ן
ן
ן
ן
ן
ן
ן
2 mismatches
4 gaps (insertions/deletions)
10 perfect matches
2 mismatches
6 gaps (insertions/deletions)
9 perfect matches
The scoring system
Match	 +1
Mismatch -2
Gap -1
Score: = (+1)x9 + (-2)x2 + (-1)x6 = -1
Score: = (+1)x10 + (-2)x2 + (-1)x4 = 2
The alignment with the highest score is chosen

## Slide 12
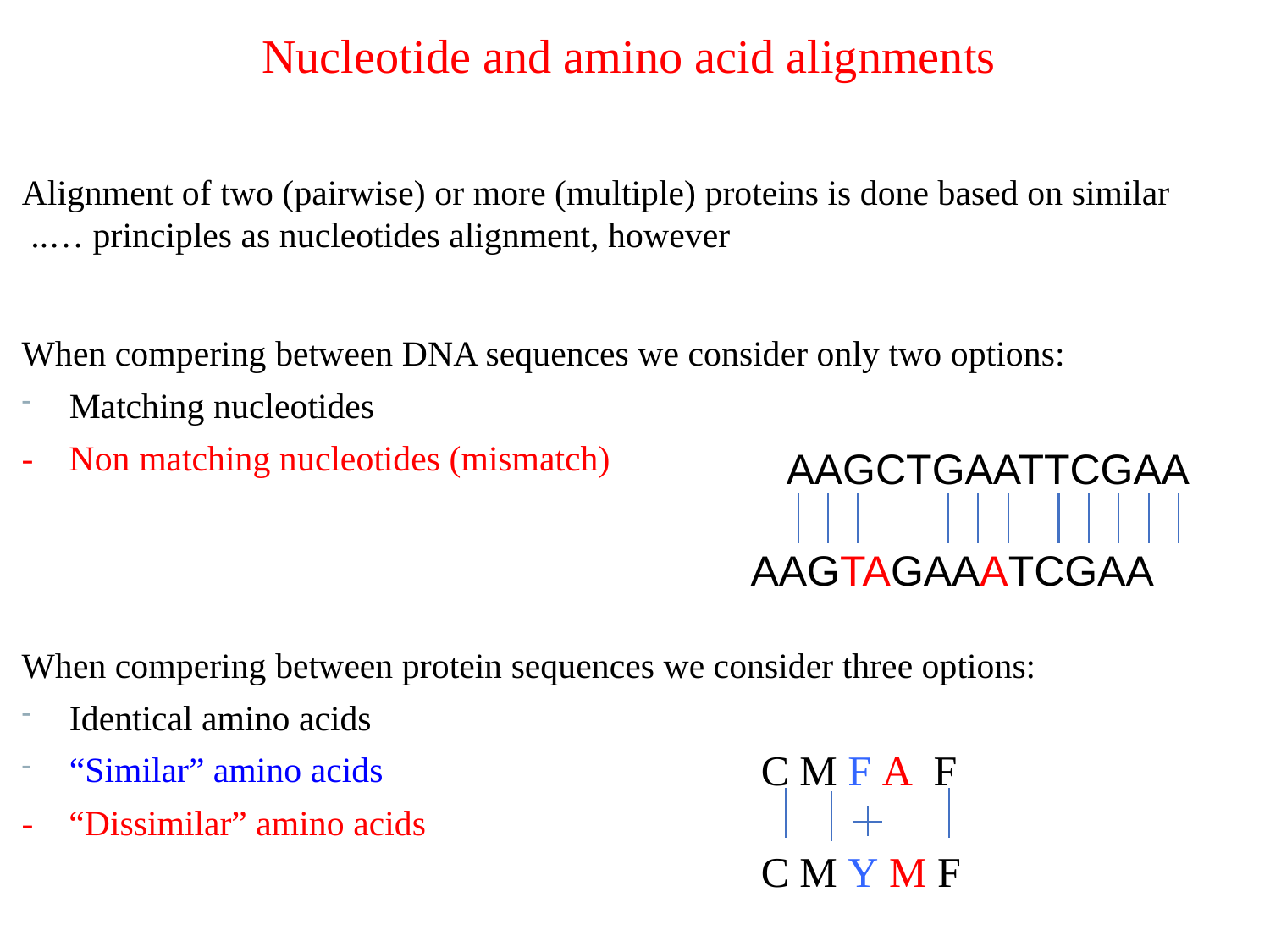

Nucleotide and amino acid alignments
Alignment of two (pairwise) or more (multiple) proteins is done based on similar principles as nucleotides alignment, however …..
When compering between DNA sequences we consider only two options:
Matching nucleotides
- Non matching nucleotides (mismatch)
AAGCTGAATTCGAA
AAGTAGAAATCGAA
When compering between protein sequences we consider three options:
Identical amino acids
“Similar” amino acids
- “Dissimilar” amino acids
 C M F A F
 C M Y M F

## Slide 13
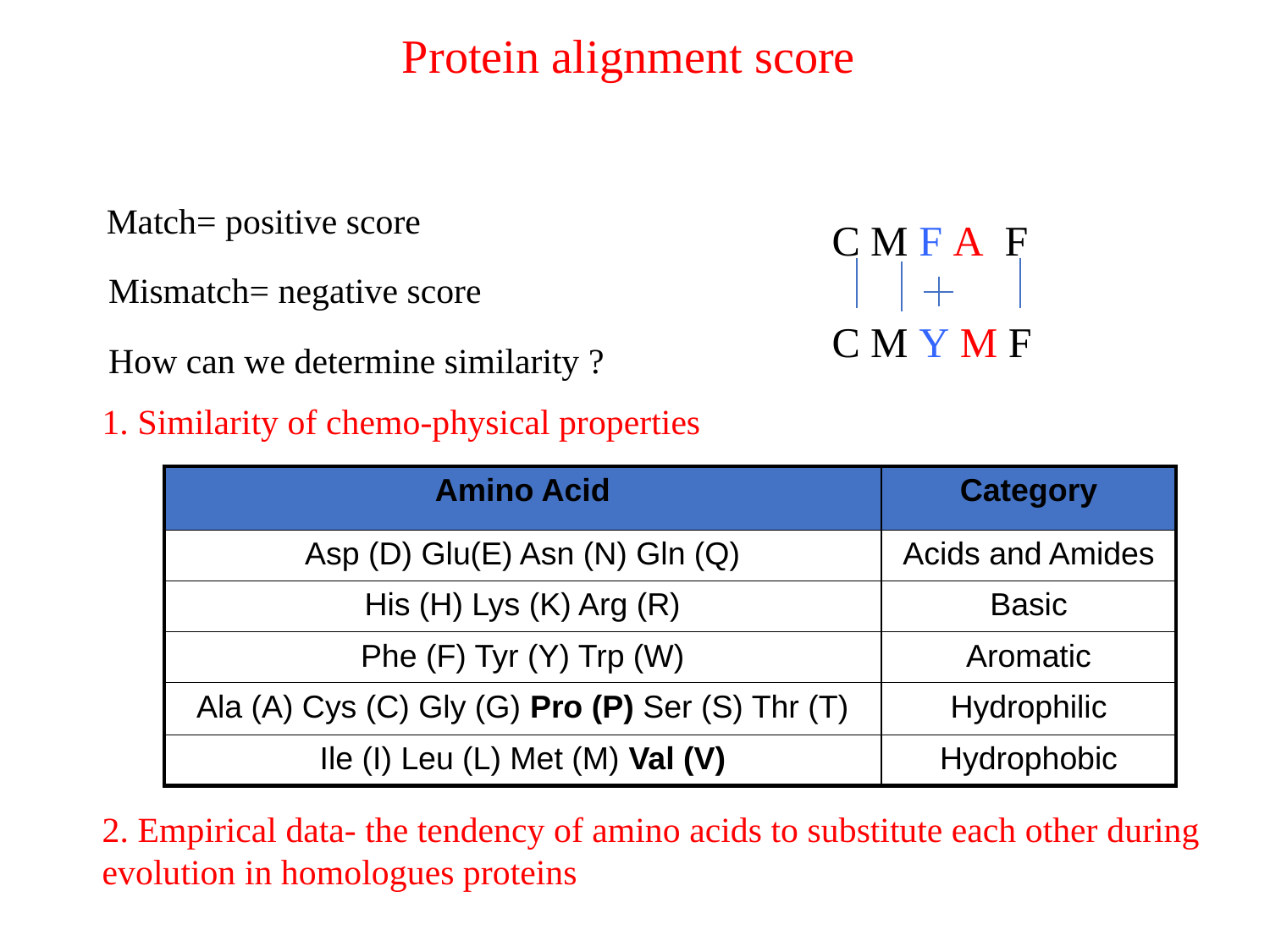

Protein alignment score
Match= positive score
 C M F A F
 C M Y M F
Mismatch= negative score
How can we determine similarity ?
1. Similarity of chemo-physical properties
| Amino Acid | Category |
| --- | --- |
| Asp (D) Glu(E) Asn (N) Gln (Q) | Acids and Amides |
| His (H) Lys (K) Arg (R) | Basic |
| Phe (F) Tyr (Y) Trp (W) | Aromatic |
| Ala (A) Cys (C) Gly (G) Pro (P) Ser (S) Thr (T) | Hydrophilic |
| Ile (I) Leu (L) Met (M) Val (V) | Hydrophobic |
2. Empirical data- the tendency of amino acids to substitute each other during evolution in homologues proteins

## Slide 14
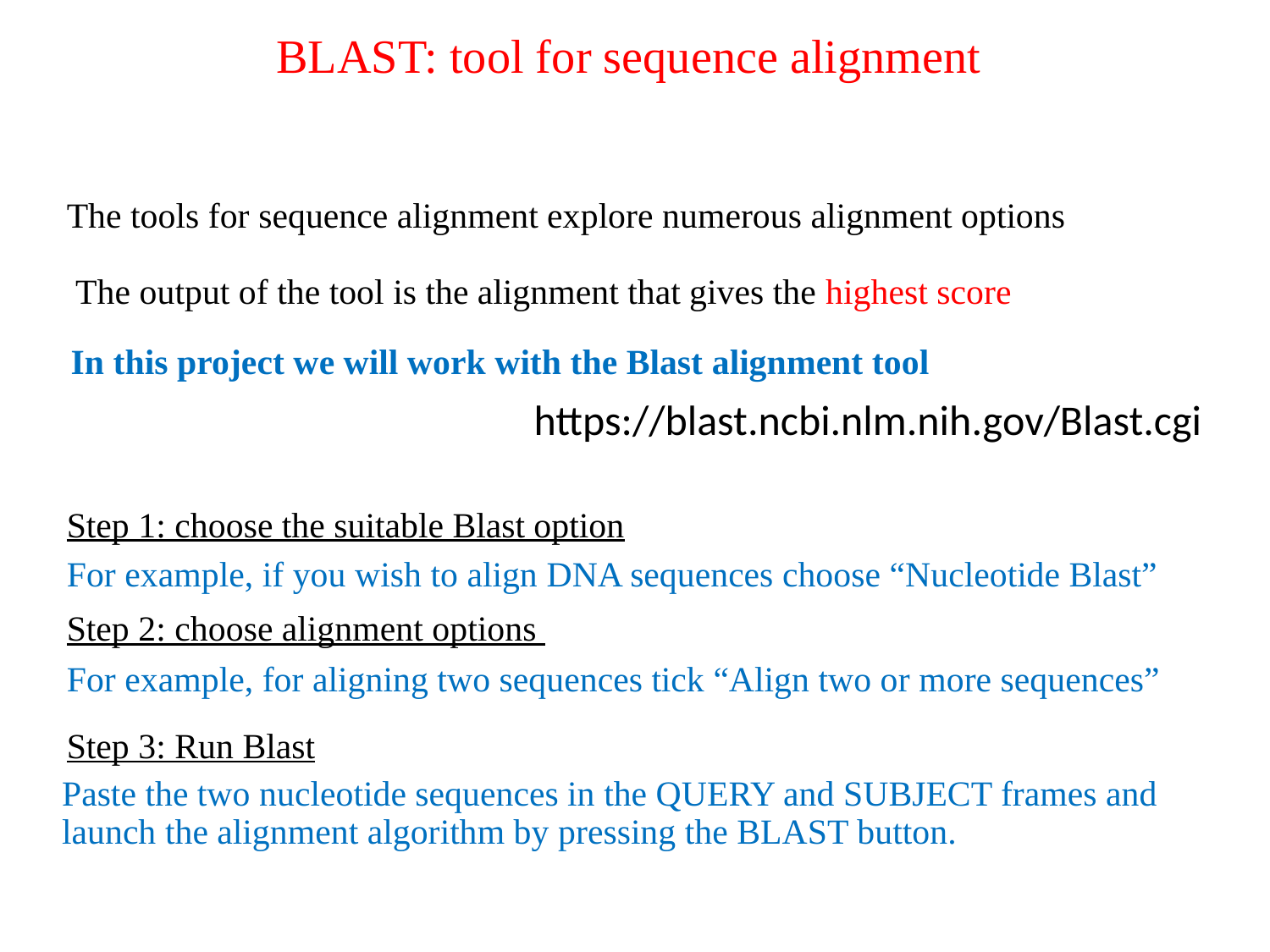

BLAST: tool for sequence alignment
The tools for sequence alignment explore numerous alignment options
The output of the tool is the alignment that gives the highest score
In this project we will work with the Blast alignment tool
https://blast.ncbi.nlm.nih.gov/Blast.cgi
Step 1: choose the suitable Blast option
For example, if you wish to align DNA sequences choose “Nucleotide Blast”
Step 2: choose alignment options
For example, for aligning two sequences tick “Align two or more sequences”
Step 3: Run Blast
Paste the two nucleotide sequences in the QUERY and SUBJECT frames and
launch the alignment algorithm by pressing the BLAST button.

## Slide 15
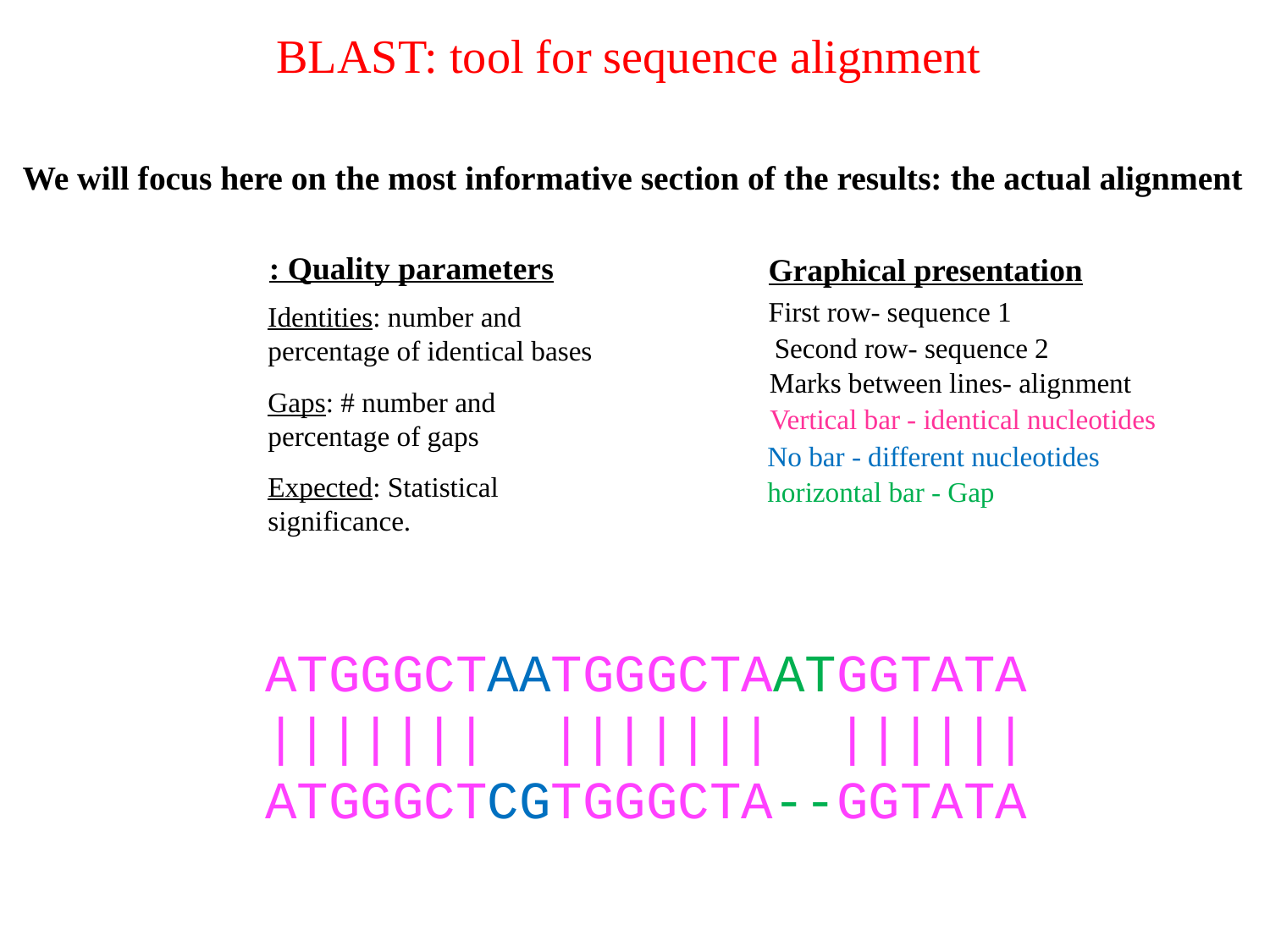

BLAST: tool for sequence alignment
We will focus here on the most informative section of the results: the actual alignment
Quality parameters :
Graphical presentation
First row- sequence 1
Identities: number and percentage of identical bases
Second row- sequence 2
Marks between lines- alignment
Gaps: # number and percentage of gaps
Vertical bar - identical nucleotides
No bar - different nucleotides
Expected: Statistical significance.
 horizontal bar - Gap
ATGGGCTAATGGGCTAATGGTATA
||||||| ||||||| ||||||
ATGGGCTCGTGGGCTA--GGTATA
